# Leakage and the reproducibility crisis in machine-learning-based science

**DOI:** 10.1016/j.patter.2023.100804

**Published:** 2023-08-04

**Authors:** Sayash Kapoor, Arvind Narayanan

**Affiliations:** 1 Department of Computer Science and Center for Information Technology Policy, Princeton University, Princeton, NJ 08540, USA

**Keywords:** reproducibility, machine learning, leakage

## Abstract

Machine-learning (ML) methods have gained prominence in the quantitative sciences. However, there are many known methodological pitfalls, including data leakage, in ML-based science. We systematically investigate reproducibility issues in ML-based science. Through a survey of literature in fields that have adopted ML methods, we find 17 fields where leakage has been found, collectively affecting 294 papers and, in some cases, leading to wildly overoptimistic conclusions. Based on our survey, we introduce a detailed taxonomy of eight types of leakage, ranging from textbook errors to open research problems. We propose that researchers test for each type of leakage by filling out model info sheets, which we introduce. Finally, we conduct a reproducibility study of civil war prediction, where complex ML models are believed to vastly outperform traditional statistical models such as logistic regression (LR). When the errors are corrected, complex ML models do not perform substantively better than decades-old LR models.

## Highlights

• Data leakage is a flaw in machine learning that leads to overoptimistic results
• Our survey of prior reviews shows leakage affects 294 papers across 17 scientific fields
• We provide a taxonomy of leakage and introduce model info sheets to mitigate it
• We show how leakage can lead to overoptimism with a case study on civil war prediction

## The bigger picture

Machine learning (ML) is widely used across dozens of scientific fields. However, a common issue called “data leakage” can lead to errors in data analysis. We surveyed a variety of research that uses ML and found that data leakage affects at least 294 studies across 17 fields, leading to overoptimistic findings. We classified these errors into eight different types. We propose a solution: model info sheets that can be used to identify and prevent each of these eight types of leakage. We also tested the reproducibility of ML in a specific field: predicting civil wars, where complex ML models were thought to outperform traditional statistical models. Interestingly, when we corrected for data leakage, the supposed superiority of ML models disappeared: they did not perform any better than older methods. Our work serves as a cautionary note against taking results in ML-based science at face value.

## Teaser

Kapoor and Narayanan show that leakage is a widespread failure mode in machine-learning (ML)-based science. Based on a survey of past reviews, they find that it affects at least 294 papers across 17 disciplines. They provide a taxonomy of eight types of leakage and propose model info sheets to mitigate it. They show that leakage can lead to severe overoptimism through a case study of civil war prediction. Several papers claimed that ML models drastically outperform older regression models. This is no longer the case when leakage is fixed.

## Introduction

There has been a marked shift toward the paradigm of predictive modeling across quantitative science fields. This shift has been facilitated by the widespread use of machine learning (ML) methods. However, pitfalls in using ML methods have led to exaggerated claims about their performance. Such errors can lead to a feedback loop of overoptimism about the paradigm of prediction, especially because non-replicable publications tend to be cited more often than replicable ones.^1^ It is therefore important to examine the reproducibility of findings in communities adopting ML methods.

*Scope.* We focus on reproducibility issues in ML-based science, which involves *making a scientific claim using the performance of the ML model as evidence*. There is a better-known reproducibility crisis in research that uses traditional statistical methods.^2^ We also situate our work in contrast with other ML domains, such as methods research (creating and improving widely applicable ML methods), ethics research (studying the ethical implications of ML methods), engineering applications (building or improving a product or service), and modeling contests (improving predictive performance on a fixed dataset created by an independent third party). Investigating the validity of claims in all these areas is important, and there is ongoing work to address reproducibility issues in these domains.^3^^,^^4^^,^^5^^,^^6^

We define a research finding as reproducible if the code and data used to obtain the finding are available and the data are correctly analyzed.^4^^,^^7^^,^^8^ This is a broader definition than computational reproducibility, when the results in a paper can be replicated using the exact code and dataset provided by the authors (see supplemental experimental procedures, section S1).

*Leakage.* Data leakage is a spurious relationship between the independent variables and the target variable that arises as an artifact of the data collection, sampling, or pre-processing strategy. Because the spurious relationship will not be present in the distribution about which scientific claims are made, leakage usually leads to inflated estimates of model performance.

Data leakage has long been recognized as a leading cause of errors in ML applications.^9^ In formative work on leakage, Kaufman et al.^10^ provide an overview of different types of error and give several recommendations for mitigating these errors. Since this paper was published, the ML community has investigated leakage in several engineering applications and modeling competitions.^11^^,^^12^^,^^13^^,^^14^^,^^15^ However, leakage occurring in ML-based science has not been comprehensively investigated. As a result, mitigations for data leakage in scientific applications of ML remain understudied.

**Figure 1:**
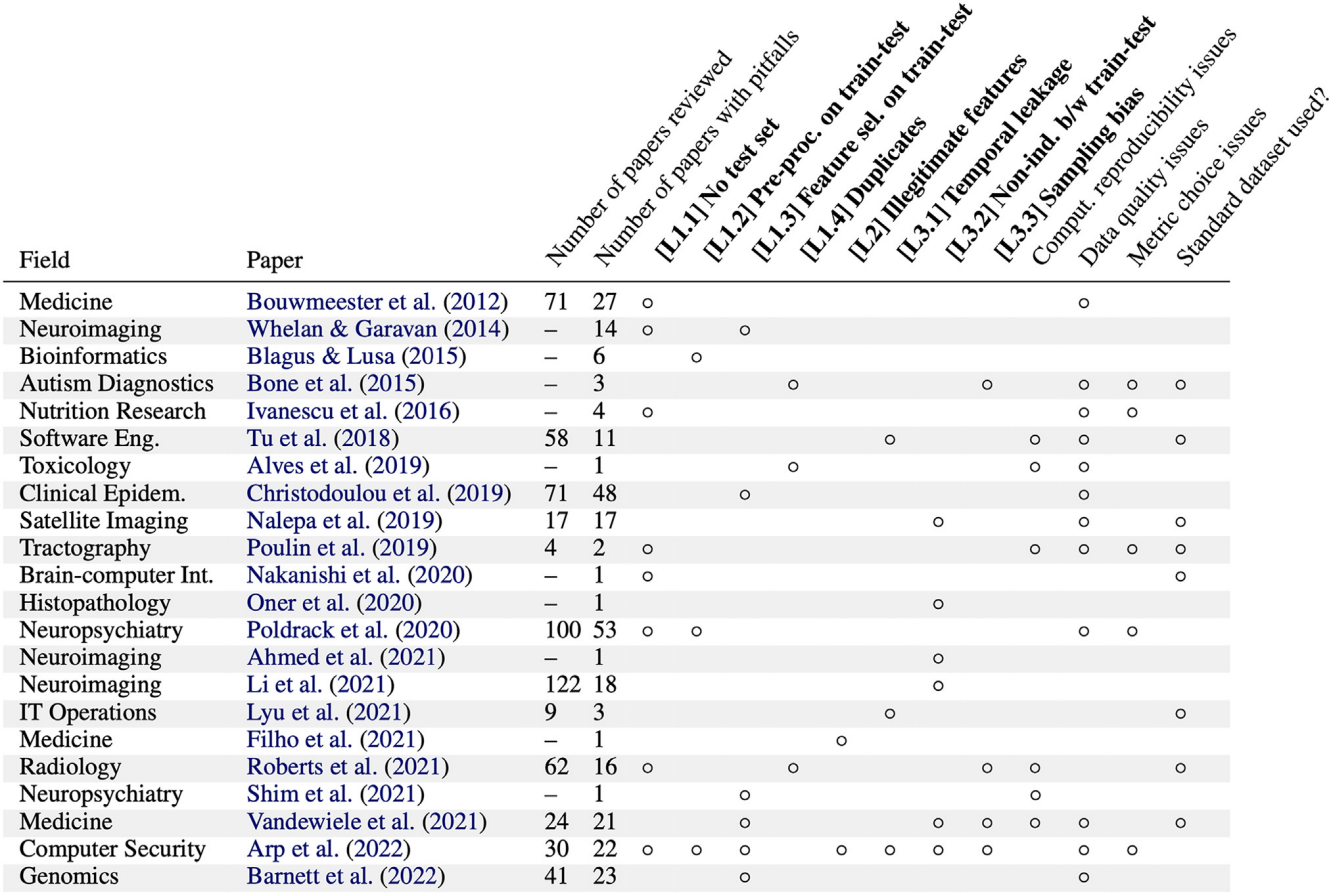
Survey of 22 papers that identify pitfalls in the adoption of ML methods across 17 fields, collectively affecting 294 papers In each field, papers adopting ML methods suffer from data leakage. The column headings for types of data leakage, shown in bold, are based on our taxonomy of data leakage. We also highlight other issues that are reported in the papers: (1) computational reproducibility (the lack of availability of code, data, and computing environment to reproduce the exact results reported in the paper); (2) data quality (e.g., small size or large amounts of missing data); (3) metric choice (using incorrect metrics for the task at hand, e.g., using accuracy for measuring model performance in the presence of heavy class imbalance); and (4) standard dataset use, where issues are found despite the use of standard datasets in a field.^16^^,^^17^^,^^18^^,^^19^^,^^20^^,^^21^^,^^22^^,^^23^^,^^24^^,^^25^^,^^26^^,^^27^^,^^28^^,^^29^^,^^30^^,^^31^^,^^32^^,^^33^^,^^34^^,^^35^^,^^36^^,^^37^

In this paper, we systematically investigate reproducibility issues in ML-based science as a result of data leakage. Our main contributions are as follows:

1. **A survey and taxonomy of reproducibility issues caused by leakage.** We provide evidence for a growing reproducibility crisis in ML-based science. Through a survey of literature in research communities that adopted ML methods, we find 22 papers across 17 fields where leakage has been found, collectively affecting 294 papers (Figure 1). We highlight that data leakage mitigation strategies developed for other ML applications, such as modeling contests and engineering applications, often do not translate to ML-based science. Based on our survey, we present a fine-grained taxonomy of eight types of leakage that range from textbook errors to open research problems.

2. **Model info sheets to detect and prevent leakage.** Current standards for reporting model performance in ML-based science often fall short in addressing issues caused by leakage. Specifically, checklists and model cards are one way to provide standard best practices for reporting details about ML models.^38^^,^^39^^,^^40^ However, current efforts do not address issues arising because of leakage. Further, most checklists currently in use are not developed for ML-based science in general but rather for specific scientific or research communities.^4^^,^^38^ As a result, best practices for model reporting in ML-based science are underspecified.

We introduce model info sheets to detect and prevent leakage in ML-based science. They are inspired by the model cards in Mitchell et al.^40^ Filling out a model info sheet requires the researcher to provide precise arguments to justify that models used for making scientific claims do not suffer from leakage, by answering 21 questions based on our taxonomy of leakage.

3. **An empirical case study of leakage in civil war prediction.** For an in-depth look at the impact of reproducibility errors, we undertake a reproducibility study in civil war prediction, a subfield of political science where ML models are believed to vastly outperform older statistical models such as logistic regression (LR). We perform a systematic review to find papers on civil war prediction and find that all papers in our review claiming the superior performance of complex ML models compared with KR models fail to reproduce because of data leakage.

Each of these papers was published in top political science journals. Leakage affects complex ML models, as well as simpler LR models. But when the errors caused by leakage are corrected, ML models no longer perform substantively better than decades-old LR models.

## Results

### Evidence of a reproducibility crisis

Many scientific fields have adopted ML methods and the paradigm of predictive modeling.^41^^,^^42^^,^^43^^,^^44^^,^^45^^,^^46^ We find at least three main uses of ML models in scientific literature. First, models that are better at prediction are thought to enable an improved understanding of scientific phenomena.^47^ Second, especially when used in medical fields, models with higher predictive accuracy can aid in research and development of better diagnostic tools.^48^ Finally, ML-based methods have also been used to investigate the inherent predictability of phenomena, especially for predicting social outcomes.^49^ The increased adoption of ML methods in science motivates our investigation of reproducibility issues in ML-based science.

#### Data leakage causes irreproducible results

Researchers in many communities have already documented reproducibility failures in ML-based science within their fields. Here we conduct a cross-disciplinary analysis by building on these individual reviews. This enables us to highlight the scale and scope of the crisis, identify common patterns, and make progress toward a solution.

When searching for past literature that documents reproducibility failures in ML-based science, we found that different fields often use different terms to describe pitfalls and errors. This makes it difficult to conduct a systematic search to find papers with errors. Therefore, we do not present our results as a systematic meta-review of leakage from a coherent sample of papers but rather as a lower bound of reproducibility issues in ML-based science. In addition, most reviews look only at the *content* of the papers and not the code and data provided with the papers to check for errors. This leads to under-counting the number of affected papers, because the code might have errors that are not apparent from reading the papers.

We find 22 papers from 17 fields that outline errors in ML-based science in their field, collectively affecting 294 papers. A prominent finding that emerges is that data leakage is a pitfall in every single case. Our findings present a worrying trend for the reproducibility of ML-based science.

Note that leakage is one of many causes of irreproducible results. Other factors, such as the lack of available code and data, can also lead to irreproducibility, and there are several studies investigating these shortcomings.^50^^,^^51^ We discuss our choice of terminology in detail in the supplemental experimental procedures (section S1).

The results from our survey are presented in Figure 1. Columns in bold represent different types of leakage. The last four columns represent other common trends in the papers we study. For systematic reviews, we report the number of papers reviewed. Each paper in our survey highlights issues with leakage, with six papers highlighting the presence of multiple types of leakage in their field.

#### Data leakage mitigations for other ML applications do not apply to scientific research

Most previous research and writing on data leakage has focused on mitigating data leakage in engineering settings or predictive modeling competitions.^10^^,^^11^^,^^12^ However, the taxonomy of data leakage outlined in this body of work does not address all types of leakage that we identify in our survey. In particular, we find that leakage can result from a difference between the distribution of the test set and the distribution of scientific interest. Robustness to distribution shift is an area of ongoing research in ML methods and is as such an open problem.^52^ In addition, these settings are very different from scientific research, and mitigations for data leakage in modeling competitions, as well as engineering applications of ML, often do not translate into strategies for mitigating data leakage in ML-based science.

##### Leakage in modeling competitions

In predictive modeling competitions, dataset creation and model evaluation are left to impartial third parties who have the expertise and incentives to avoid errors. Within this framework, none of the participants have access to the held-out evaluation set before the competition ends. In contrast, in most ML-based science, the researcher has access to the entire dataset while creating the ML models. Leakage often occurs because of the researcher having access to the entire dataset during the modeling process.

##### Leakage in engineering applications

Leakage in real-world applications has led to exaggerated performance estimates, even in consequential settings such as child maltreatment prediction.^53^ One of the most common recommendations for detecting and mitigating leakage is to deploy the ML model at a limited scale in production. This advice is applicable only to engineering applications of ML, where the end goal is not to gain insights about a particular process but rather to serve as a component in a product. Often, a rough idea of model performance is enough to decide whether a model is good enough to be deployed in a product. Contrarily, ML-based science involves making a scientific claim using the performance of the ML model as evidence. In addition, engineering applications of ML often operate in a rapidly changing context and have access to large datasets, so small differences in performances are often not as important, whereas scientific claims are sensitive to small performance differences between ML models.

#### Why do we call it a reproducibility crisis?

We say that ML-based science is suffering from a reproducibility crisis for two related reasons. First, our results show that reproducibility failures in ML-based science are systemic. In nearly every scientific field that has carried out a systematic study of reproducibility issues, papers are plagued by common pitfalls. In many systematic reviews, a majority of the papers reviewed suffer from these pitfalls. Similar problems are likely to arise in many fields that are adopting ML methods. Second, despite the urgency of addressing reproducibility failures, there are no systemic solutions that have been deployed for these failures. Scientific communities are discovering the same failure modes across disciplines but have yet to converge on best practices for avoiding reproducibility failures.

Calling attention to and addressing these widespread failures is vital to maintaining public confidence in ML-based science. At the same time, the use of ML methods is still in its infancy in many scientific fields. Addressing reproducibility failures pre-emptively in such fields can correct a lot of scientific research that would otherwise be flawed.

### Toward a solution: A taxonomy of data leakage

We now provide our taxonomy of data leakage errors in ML-based science. Such a taxonomy can enable a better understanding of why leakage occurs and inform potential solutions. Our taxonomy is comprehensive and addresses data leakage arising during the data collection, pre-processing, modeling, and evaluation steps. In particular, our taxonomy addresses all cases of data leakage that we found in our survey (Figure 1). Some of the categories in our taxonomy, e.g., sampling bias [L3.3], were not considered types of leakage in prior work, but they have the same cause as other categories of leakage: spurious correlations between the outcome variables and the features. They also have the same effect: they lead to overestimates of model performance.

**[L1] Lack of clean separation of training and test dataset.** If the training dataset is not separated from the test dataset during all pre-processing, modeling, and evaluation steps, the model has access to information in the test set before its performance is evaluated. Because the model has access to information from the test set at training time, the model learns relationships between the predictors and the outcome that would not be available in additional data drawn from the distribution of interest. The performance of the model on these data therefore does not reflect how well the model would perform on a new test set drawn from the same distribution of data. This can happen in several ways, such as:

**[L1.1] No test set.** Using the same dataset for training and testing the model is a textbook example of overfitting, which leads to overoptimistic performance estimates.^54^

**[L1.2] Pre-processing on training and test set.** Using the entire dataset for any pre-processing steps, such as imputation or over/under sampling, results in leakage. For instance, using oversampling before splitting the data into training and test sets leads to an imperfect separation between the training and test sets because data generated using oversampling from the training set will also be present in the test set.

**[L1.3] Feature selection on training and test set.** Feature selection on the entire dataset results in using information about which feature performs well on the test set to make a decision about which features should be included in the model.

**[L1.4] Duplicates in datasets.** If a dataset with duplicates is used for the purposes of training and evaluating an ML model, the same data could exist in the training set and the test set.

**[L2] Model uses features that are not legitimate.** If the model has access to features that should not be legitimately available for use in the modeling exercise, this could result in leakage. One instance when this can happen is if a feature is a proxy for the outcome variable.^10^ For example, Filho et al.^55^ find that a recent study included the use of anti-hypertensive drugs as a feature for predicting hypertension. Such a feature could lead to leakage because the model would not have access to this information when predicting the health outcome for a new patient. Further, if the fact that a patient uses anti-hypertensive drugs is already known at prediction time, the prediction of hypertension becomes a trivial task.

The judgment of whether the use of a given feature is legitimate for a modeling task requires domain knowledge and can be highly problem specific. As a result, we do not provide sub-categories for this sort of leakage. Instead, we suggest that researchers clearly specify which features are suitable for a modeling task and justify their choice using domain expertise.

**[L3] Test set is not drawn from the distribution of scientific interest.** The distribution of data on which the performance of an ML model is evaluated differs from the distribution of data about which the scientific claims are made. The performance of the model on the test set does not correspond to its performance on data drawn from the distribution of scientific interest.

**[L3.1] Temporal leakage.** When an ML model is used to make predictions about a future outcome of interest, the test set should not contain any data from a date before the training set. If the test set contains data from before the training set, the model is built using data “from the future” that it should not have access to during training and can cause leakage.

**[L3.2] Nonindependence between training and test samples.** Nonindependence between training and test samples constitutes leakage, unless the scientific claim is about a distribution that has the same dependence structure. In the extreme (but unfortunately common) case, training and test samples come from the same people or units. For example, Oner et al.^56^ find that a recent study on histopathology uses different observations of the same patient in the training and test sets. In this case, the scientific claim is being made about the ability to predict gene mutations in new patients; however, it is evaluated on data from old patients (i.e., data from patients in the training set), leading to a mismatch between the test set distribution and the scientific claim. Similarly, for predicting protein function, the family of the protein can lead to dependencies if proteins from the same family are split across the training and test sets.^57^ The train-test split should account for the dependencies in the data to ensure correct performance evaluation. Methods such as “block cross-validation” can partition the dataset strategically so that the performance evaluation does not suffer from data leakage and overoptimism.^58^^,^^59^ Handling nonindependence between the training and test sets in general (i.e., without any assumptions about independence in the data) is a hard problem, because we might not know the underlying dependency structure of the task in many cases.^60^

**[L3.3] Sampling bias in test distribution.** Sampling bias in the choice of test dataset can lead to data leakage. One example of sampling bias is spatial bias, which refers to choosing the test data from a geographic location but making claims about model performance in other geographic locations. Another example is selection bias, which entails choosing a non-representative subset of the dataset for evaluation. For example, Bone et al.^61^ highlight that in a study on predicting autism using ML models, excluding the data corresponding to borderline cases of autism leads to leakage because the test set is no longer representative of the general population about which claims are made. In addition, borderline cases of autism are often the trickiest to diagnose, so excluding them from the evaluation set is likely to lead to overoptimistic results. Cases of leakage caused by sampling bias can often be subtle. For example, Zech et al.^62^ find that models for pneumonia prediction trained on images from one hospital do not generalize to images from another hospital because of subtle differences in how images are generated in each hospital.

A model may have leakage when the distribution about which the scientific claim is made does not match the distribution from which the evaluation set is drawn. ML models may also suffer from a related but distinct limitation: the lack of generalization when we try to apply a result about one population to another similar but distinct population. Several issues with the generalization of ML models operating under a distribution shift have been highlighted in ML methods research, such as fragility toward adversarial examples,^63^ image distortion and texture,^64^ and overinterpretation.^65^ Robustness to distribution shift is an ongoing area of work in ML methods research. Even slight shifts in the target distribution can cause performance estimates to change drastically.^66^ Despite ongoing work to create ML methods that are robust to distribution shift, best practices to deal with distribution shift currently include testing the ML models on the data from the distribution we want to make claims about.^52^ In ML-based science, where the aim is to create generalizable knowledge, we should take results that claim to generalize to a different population from the one models were evaluated on with caution.

#### Other issues identified in our survey

##### Computational reproducibility issues

Computational reproducibility of a finding refers to sharing the complete code and data needed to reproduce the findings reported in a paper exactly. This is important to enable external researchers to reproduce results and verify their correctness. Five papers in our survey outlined the lack of computational reproducibility in their field.

##### Data quality issues

Access to good-quality data is essential for creating ML models.^67^^,^^68^ Issues with the quality of the dataset could affect the results of ML-based science. Ten papers in our survey highlighted data quality issues such as not addressing missing values in the data, the small size of datasets compared with the number of predictors, and the outcome variable being a poor proxy for the phenomenon being studied.

##### Metric choice issues

A mismatch between the metric used to evaluate performance and the scientific problem of interest leads to issues with performance claims. For example, using accuracy as the evaluation metric with a heavily imbalanced dataset leads to overoptimistic results, because the model can get a high accuracy score by always predicting the majority class. Four papers in our survey highlighted metric choice issues.

##### Use of standard datasets

Reproducibility issues arose despite the use of standard, widely used datasets, often because of the lack of standard modeling and evaluation procedures such as fixing the train-test split and evaluation metric for the dataset. Seven papers in our survey highlighted that issues arose despite the use of standard datasets.

### Model info sheets for detecting and preventing leakage

Our taxonomy of data leakage highlights several failure modes that are prevalent in ML-based science. To detect cases of leakage, we provide a template for a model info sheet to accompany scientific claims using predictive modeling as a supplemental document (supplemental experimental procedures, section S4). The template consists of 21 questions that elicit precise arguments needed to justify the absence of leakage.

#### Prior work on model cards and reporting standards

Our proposal is inspired by prior work on model cards and checklists, which we now review. Mitchell et al.^40^ introduced model cards for reporting details about ML models, with a focus on precisely reporting the intended use cases of ML models. They also addressed fairness and transparency concerns: they require that the performance of ML models on different groups of users (e.g., on the basis of race, gender, and age) is reported and documented transparently. These model cards complement the datasheets introduced by Gebru et al.^69^ to document details about datasets in a standard format.

The use of checklists has also been impactful in improving reporting practices in the few fields that have adopted them.^70^ Although checklists and model cards provide concrete best practices for reporting standards,^38^^,^^39^^,^^40^^,^^71^ current efforts do not address pitfalls arising because of leakage. Further, even though several scientific fields, especially those related to medicine, have adopted checklists to improve reporting standards, most checklists are developed for specific scientific or research communities instead of ML-based science in general.

#### Scientific arguments to surface and prevent leakage

When ML models are used to make scientific claims, it is not enough to simply separate the training and test sets and report performance metrics on the test set. Unlike research in ML methods, where a model’s performance on a hypothetical task (i.e., one that is not linked to a specific scientific claim) is still of interest to the researcher in some cases,^72^ in ML-based science, claims about a model’s performance need to be connected to scientific claims using explicit arguments. The burden of proof for ensuring the correctness of these arguments is on the researcher making the scientific claims.^73^

In our model info sheet, we ask researchers to answer 21 questions. These questions help them present three arguments that are essential for determining that scientific results that use ML methods do not suffer from data leakage. Note that most ML-based science papers do not present any of the three arguments, although they sometimes partially address the first argument (clean train-test separation) by reporting out-of-sample prediction performance. The arguments below are based on our taxonomy of data leakage issues and inform the main sections of the model info sheet.

**[L1] Clean train-test separation.** The researcher needs to argue why the test set does not interact with training data during any of the pre-processing, modeling, or evaluation steps to ensure a clean train-test separation.

**[L2] Each feature in the model is legitimate.** The researcher needs to argue why each feature used in their model is legitimate, i.e., a claim made using each feature is of scientific interest. Note that some models might use hundreds of features. In such cases, it is even more important to reason about the correctness of the features used, because the incorrect use of a single feature in the model can cause leakage. That said, the same argument for why a feature is legitimate can often apply to a whole set of features. For example, for a study using individuals’ location history as a feature vector, the use of the entire vector can be justified together. Note that we do not ask for the researcher to list each feature used in their model; rather, we ask that the justification provided for the legitimacy of the features used in their model should cover every feature used in their model.

**[L3] Test set is drawn from the distribution of scientific interest.** If the distribution about which the scientific claims are made is different from the one on which the model is tested, then any claims about the performance of an ML model on the evaluation step fall short. The researcher needs to justify that the test set is drawn from the distribution of scientific interest and there is no selection or sampling bias in the data collection process. This step can help clarify the distribution regarding which scientific claims are being made and detect temporal leakage.

#### Model info sheets and our theory of change

Model info sheets can influence research practices in two ways: first, researchers who introduce a scientific model alongside a paper can use model info sheets to detect and prevent leakage in their models. These info sheets can be included as supplementary materials with their paper for transparently reporting details about their models. In scientific fields where the use of ML methods is not yet widespread, using transparent reporting practices at an early stage could enable easier adoption and more trust in ML methods. This would also help assuage reviewer concerns about reproducibility.

Second, journal submission guidelines could encourage or require authors to fill out model info sheets if a paper does not transparently report how the model was created. In this case, model info sheets can be used to start a conversation between authors and reviewers about the details of the models introduced in a paper. Current peer-review practices often do not require the authors to disclose any code or data during the review process.^74^ Even if the code and data are available to reviewers, reproducing results and spotting errors in code is a time-consuming process that often cannot be carried out under current peer-review practices. Model info sheets offer a middle ground: they could enable a closer scrutiny of methods without making the process onerous for reviewers.

#### Limitations of model info sheets

Although model info sheets can enable the detection of all types of leakage we identify in our survey, they suffer from limitations owing to the lack of computational reproducibility of results in scientific research, incorrect claims made in model info sheets, and the lack of expertise of authors and reviewers.

First, the claims made in model info sheets cannot be verified in the absence of computational reproducibility. That is, unless the code, data, and computing environment required to reproduce the results in a paper are made available, there is no way to ascertain whether model info sheets are filled out correctly. Ensuring the computational reproducibility of results therefore remains an important goal for improving scientific research standards.

Second, incorrect claims made in model info sheets might provide false assurances to reviewers about the correctness of the claims made in a paper. However, by requiring authors to precisely state details about their modeling process, model info sheets enable incorrect claims to be challenged more directly than in status quo, where details about the modeling process are often left undisclosed.

Filling out and evaluating model info sheets requires some expertise in ML. In fields where both authors and reviewers lack any ML expertise, subtle cases of leakage might slip under the radar despite the use of model info sheets. In such cases, we hope that model info sheets released publicly along with papers will enable discourse within scientific communities on the shortcomings of scientific models.

Finally, we acknowledge that our understanding of leakage may evolve, and model info sheets may need to evolve with it. To that end, we have versioned model info sheets, and plan to update them as we continue to better understand leakage in ML-based science.

### A case study of civil war prediction

To understand the impact of data leakage, we undertake a reproducibility study in a field where ML models are believed to vastly outperform older statistical models such as LR for predictive modeling: civil war prediction.

**Figure 2:**
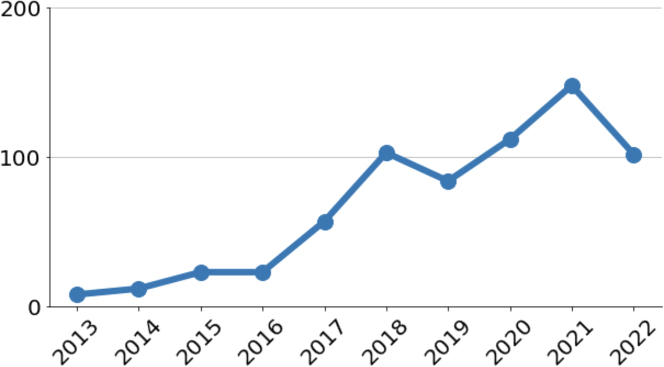
The sharp increase in civil war papers that use ML methods in the last few years The number of political science papers containing the terms “civil war” and “machine learning” in the dimensions database of academic research.^104^

Over the last few years, this field has switched to predictive modeling using complex ML models such as Random Forests and Adaboost instead of LR (see Figure 2), with several papers claiming near-perfect performance of these models for civil war prediction.^75^^,^^76^^,^^77^^,^^78^ The goal of these papers is to predict civil war in a region and time period using features such as GDP, poverty rates, whether it is a democracy, etc. This is in contrast with the field’s earlier focus on understanding and explaining past conflicts. Table S6 gives an overview of the training data used for the papers we considered. For a detailed overview of the recent turn to predictive modeling in this field, see Bara.^79^

Although the literature we reviewed in our survey highlighted the pitfalls in adopting ML methods (Figure 1), we go further than most previous research to investigate whether the claims made in the reviewed studies survive once the errors are corrected.

#### Systematic search of predictive modeling literature in civil war research

We conducted a systematic search to find relevant literature (detailed in supplemental experimental procedures, section S2.1). This yielded 124 papers. We narrowed this list to the 12 papers that focused on predicting civil war, evaluated performance using a train-test split, and shared the complete code and data. For these 12, we attempted to identify errors and reproducibility issues from the text and by reviewing the code provided with the papers. When we identified errors, we re-analyzed the data with the errors corrected.

##### Finding 1: Data leakage causes irreproducible results

**Figure 3:**
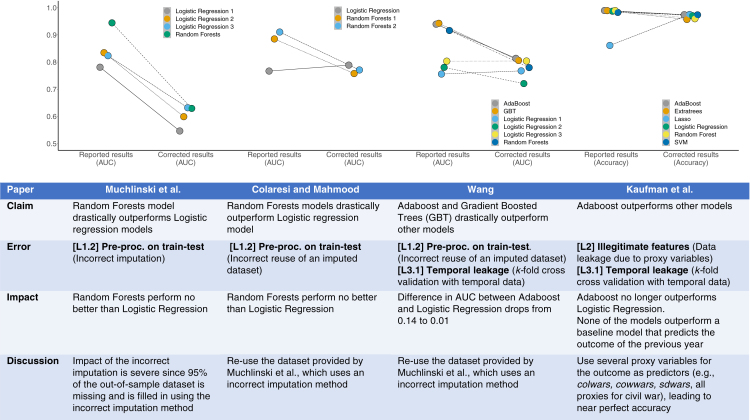
A comparison of reported and corrected results in civil war prediction papers published in top political science journals The main findings of each of these papers are invalid due to various forms of data leakage: Muchlinski et al.^75^ impute the training and test data together, Colaresi and Mahmood^76^ and Wang^77^ incorrectly reuse an imputed dataset, and Kaufman et al.^78^ use proxies for the target variable that cause data leakage. When we correct these errors, complex ML models (such as Adaboost and Random Forests) do not perform substantively better than decades-old logistic regression models for civil war prediction in each case. Each column in the table outlines the impact of leakage on the results of a paper. The figure above each column shows the difference in performance that results from fixing leakage.

We present our results in Figure 3. We found errors in 4 of the 12 papers—exactly the 4 papers that claimed superior performance of complex ML models over baseline LR models for predicting civil war. Each paper suffered from different forms of leakage. All 4 papers were published in top-10 journals in the fields of political science and international relations.^80^ When the errors are corrected, complex ML models perform no better than baseline LR models in each case except Wang,^77^ where the difference between the area under the curve (AUC) of the complex ML models and LR models drops from 0.14 to 0.01. This is despite the fact that the LR models were not trained to optimize predictive accuracy: they were conceived as explanatory models to understand past conflicts instead of predicting future ones.^47^^,^^81^^,^^82^

We test our model info sheets on the four civil war prediction papers with errors and find that they would detect each type of leakage we identified in these papers (supplemental experimental procedures, section S3). Note that leakage affects both simple and complex models for civil war prediction. However, because of higher model capacity, complex ML models tend to over-fit to spurious correlations more easily in this case (supplemental experimental procedures, section S2.2).

Beyond reproducibility, our results show that complex ML models are not substantively better at civil war prediction than decades-old LR models. This is consistent with similar sobering findings in other tasks involving predicting social outcomes, such as children’s life outcomes^49^ and recidivism.^83^ Although prior work has found that some fields will benefit from the use of ML methods,^84^ our findings suggest the need for tempering the optimism about predictive modeling in the field of civil war prediction and question the use of ML models in this field. We provide a detailed overview of our methodology for correcting the errors and show that our results hold under several robustness checks in the supplemental experimental procedures, section S2.

##### Finding 2: No significance testing or uncertainty quantification

We found that 9 of the 12 papers for which complete code and data were available included no significance tests or uncertainty quantification for classifier performance comparison (Table S6). Especially when sample sizes are small, significance testing and uncertainty quantification are important steps toward reproducibility.^48^^,^^85^ As an illustration, we examine this issue in detail in the case of Blair and Sambanis^86^ because their test dataset has a particularly small number of instances of civil war onset (only 11). They propose a model of civil war onset that uses theoretically informed features and report that it outperforms other baseline models of civil war onset using the AUC metric on an out-of-sample dataset. We find that the performance of their model is not significantly better than other baseline models for civil war prediction (*Z* = 0.64, 1.09, 0.42, and 0.67; p = 0.26, 0.14, 0.34, and 0.25 for a one-tailed significance test comparing the smoothed AUC performance of the model proposed in the paper—the escalation model—with other baseline models reported in their paper—*quad*, *goldstein*, *cameo*, and *average*, respectively). We implement the comparison test for smoothed receiver operating characteristic curves detailed by Robin et al.^87^ Note that we do not correct for multiple comparisons; such a correction would further reduce the significance of the results. Further, all models have large confidence intervals for their out-of-sample performance. For instance, while the smoothed AUC performance reported by the authors is 0.85, the 95% confidence interval calculated using bootstrapped test set re-sampling is 0.66–0.95.

#### Lack of standard reporting practices for ML-based science

Our hypothesis for why leakage is prevalent is that current standards for reporting model performance in ML-based science often fall short in addressing leakage. Specifically, checklists and model cards are one way to provide standard best practices for reporting details about ML models.^38^^,^^39^^,^^40^ However, current efforts do not address issues arising because of leakage. Further, most checklists currently in use are not developed for ML-based science in general but rather for specific scientific or research communities.^4^^,^^38^ As a result, best practices for model reporting in ML-based science are underspecified.

## Discussion

### Beyond leakage: Perspectives on enhancing the reproducibility of ML-based science

We found a number of other reproducibility issues in our survey not limited to leakage. Here, we present five diagnoses for reproducibility failures in fields adopting ML methods. Each of our diagnoses is paired with a recommendation to address it.

**[D1] Lack of understanding of the limits to prediction.** Recent research for predicting social outcomes has shown that even with complex models and large datasets, there are strong limits to predictive performance.^49^^,^^83^ However, results such as the better-than-human performance of ML models in perception tasks such as image classification^88^^,^^89^ give the impression of ML models surpassing human performance across tasks, which can confuse researchers about the performance they should realistically expect from ML models.

**[R1] Understand and communicate limits to prediction.** A research agenda that investigates the efficacy of ML models in tasks across scientific fields would increase our understanding of the limits to prediction. This can alleviate the overoptimism that arises from confusing progress in one task (e.g., image classification) with another (e.g., predicting social outcomes). If we can identify upper bounds on the predictive accuracy of tasks (i.e., lower bound of the Bayes Error Rate for a task), then once the achievable accuracy has been reached, we can avoid a futile effort to increase it further and can apply increased skepticism toward results that claim to violate known bounds.

**[D2] Hype, overoptimism, and publication biases.** The hype about commercial AI applications can spill over into ML-based science, leading to overoptimism about their performance. Non-replicable findings are cited more than replicable ones,^1^ which can result in feedback loops of overoptimism in ML-based science. Besides, publication biases that have been documented in several scientific fields^90^^,^^91^ can also affect ML-based science.^92^^,^^93^

**[R2] Treat results from ML-based science as tentative.** When overoptimism is prevalent in a field, it is important to engage with results emerging from the field critically. Until reproducibility issues in ML-based science are widely addressed and resolved, results from this body of work should be treated with caution. Researchers, journal editors, and policymakers who use scientific research to inform real-world policy decisions should look beyond headline performance numbers when assessing papers.

**[D3] Inadequate expertise.** The rapid adoption of ML methods in a scientific field can lead to errors. These can be caused by the lack of expertise of domain experts in using ML methods and vice versa.

**[R3] Interdisciplinary collaborations and communication of best practices.** Literature in the ML community should address the different failure modes that arise during the modeling process. Researchers with expertise in ML methods should clearly communicate best practices in deploying ML for scientific research.^94^ Having an interdisciplinary team consisting of researchers with domain expertise and ML expertise can avoid errors.

**[D4] Lack of standardization.** Several applied ML fields, such as engineering applications and modeling contests, have adopted practices such as standardized train-test splits, evaluation metrics, and modeling tasks to ensure the validity of the modeling and evaluation process.^95^^,^^96^ However, these have not yet been adopted widely in ML-based science. This leads to subtle errors in the modeling process that can be hard to detect.

**[R4] Adopt the common task framework when possible.** The common task framework allows us to compare the performance of competing ML models using an agreed-upon training dataset and evaluation metrics, a secret holdout dataset, and a public leaderboard.^97^^,^^98^ Dataset creation and model evaluation are left to impartial third parties who have the expertise and incentives to avoid errors. However, one undesirable outcome that has been observed in communities that have adopted the common task framework is a singular focus on optimizing a particular accuracy metric to the exclusion of other scientific and normatively desirable properties of models.^67^^,^^85^^,^^99^

**[D5] Lack of computational reproducibility.** The lack of computational reproducibility hinders verification of results by independent researchers. Although computational reproducibility does not mean that the code is error free, it can make the process of finding errors easier, because researchers attempting to reproduce results do not have to spend time getting the code to run.

**[R5] Ensure computational reproducibility.** Platforms such as CodeOcean,^100^ a cloud computing platform that replicates the exact computational environment used to create the original results, can be used to ensure the long-term reproducibility of results. We follow several academic journals and researchers in recommending that future research in fields using ML methods should use similar methods to ensure computational reproducibility.^74^^,^^101^

### Conclusions

The attractiveness of adopting ML methods in scientific research is in part due to the widespread availability of off-the-shelf tools to create models without expertise in ML methods.^102^ However, this laissez-faire approach leads to common pitfalls spreading to all scientific fields that use ML. So far, each research community has independently rediscovered these pitfalls. Without fundamental changes to research and reporting practices, we risk losing public trust because of the severity and prevalence of the reproducibility crisis across disciplines. Our paper is a call for interdisciplinary efforts to address the crisis by developing and driving the adoption of best practices for ML-based science. Model info sheets for detecting and preventing leakage are a first step in that direction.

## Experimental procedures

### Resource availability

#### Lead contact

Further information and requests for resources should be directed to and will be fulfilled by the lead contact, Sayash Kapoor (sayashk@princeton.edu).

#### Materials availability

This study did not generate new materials.

## Data Availability

The code and data required to reproduce our case study on civil war prediction have been uploaded to a CodeOcean capsule (CodeOcean: https://doi.org/10.24433/CO.4899453.v1).^103^ The supplemental experimental procedures (section S2) contains a detailed description of our methods and results from additional robustness checks.

## Supplemental information

Document S1. Supplemental experimental procedures, Figures S1–S3, and Tables S1–S6

Document S2. Article plus supplemental information

## References

[1] Serra-Garcia M., Gneezy U. (2021). Nonreplicable publications are cited more than replicable ones. Sci. Adv..

[2] (2015). Open Science Collaboration Estimating the reproducibility of psychological science. Science.

[3] Hullman J., Kapoor S., Nanayakkara P., Gelman A., Narayanan A. (2022). The worst of both worlds: A comparative analysis of errors in learning from data in psychology and machine learning. arXiv.

[4] Pineau, J.; Vincent-Lamarre, P.; Sinha, K.; Larivière, V.; Beygelzimer, A.; d’Alché-Buc, F.; Fox, E.; Larochelle, H. Improving Reproducibility in Machine Learning Research (A Report from the NeurIPS 2019 Reproducibility Program).Preprint at arXiv:https://doi.org/10.48550/arXiv.2003.122062003.12206 [cs, stat] 2020, arXiv: 2003.12206.

[5] Erik Gundersen O. (2021). The fundamental principles of reproducibility. Philosophical Transactions of the Royal Society.

[6] Bell S.J., Kampman O.P. (2021).

[7] Hofman J.M., Goldstein D.G., Sen S., Poursabzi-Sangdeh F., Allen J., Dong L.L., Fried B., Gaur H., Hoq A., Mbazor E. (2021). Expanding the scope of reproducibility research through data analysis replications. Organ. Behav. Hum. Decis. Process..

[8] Leek J.T., Peng R.D. (2015). Opinion: Reproducible research can still be wrong: Adopting a prevention approach. Proc. Natl. Acad. Sci. USA.

[9] Nisbet R., Elder J., Miner G. (2009).

[10] Kaufman S., Rosset S., Perlich C., Stitelman O. (2012). Leakage in data mining: Formulation, detection, and avoidance. ACM Trans. Knowl. Discov. Data.

[11] Fraser C. (2016).

[12] Ghani R., Walsh J., Wang J. (2020). http://www.rayidghani.com/2020/01/24/top-10-ways-your-machine-learning-models-may-have-leakage/).

[13] Becker D. (2018).

[14] Brownlee J. (2016).

[15] Collins-Thompson, K. Data Leakage - Module 4: Supervised Machine Learning - Part 2, en.

[16] Bouwmeester W., Zuithoff N.P.A., Mallett S., Geerlings M.I., Vergouwe Y., Steyerberg E.W., Altman D.G., Moons K.G.M. (2012). Reporting and Methods in Clinical Prediction Research: A Systematic Review. PLOS Med..

[17] Whelan R., Garavan H. (2014). When Optimism Hurts: Inflated Predictions in Psychiatric Neuroimaging. Biol. Psychiatry.

[18] Blagus R., Lusa L. (2015). Joint Use of Over- and under-Sampling Techniques and Cross-Validation for the Development and Assessment of Prediction Models. BMC Bioinformatics.

[19] Bone D., Goodwin M.S., Black M.P., Lee C.-C., Audhkhasi K., Narayanan S. (2015). Applying Machine Learning to Facilitate Autism Diagnostics: Pitfalls and Promises. J. Autism Dev. Disord..

[20] Ivanescu A.E., Li P., George B., Brown A.W., Keith S.W., Raju D., Allison D.B. (2016). The Importance of Prediction Model Validation and Assessment in Obesity and Nutrition Research. Int. J. Obes..

[21] Tu F., Zhu J., Zheng Q., Zhou M. (2018). Proceedings of the 2018 26th ACM Joint Meeting on European Software Engineering Conference and Symposium on the Foundations of Software Engineering.

[22] Alves V.M., Borba J., Capuzzi S.J., Muratov E., Andrade C.H., Rusyn I., Tropsha A. (2019). Oy Vey! A Comment on “Machine Learning of Toxicological Big Data Enables Read-Across Structure Activity Relationships Outperforming Animal Test Reproducibility. Toxicol. Sci..

[23] Christodoulou E., Ma J., Collins G.S., Steyerberg E.W., Verbakel J.Y., Van Calster B. (2019). A Systematic Review Shows No Performance Benefit of Machine Learning over Logistic Regression for Clinical Prediction Models. J. Clin. Epidemiol..

[24] Nalepa J., Myller M., Kawulok M. (2019). Validating Hyperspectral Image Segmentation. IEEE Geosci. Remote Sens. Lett..

[25] Poulin P., Jörgens D., Jodoin P.-M., Descoteaux M. (2019). Tractography and Machine Learning: Current State and Open Challenges. Magn. Reson. Imaging.

[26] Nakanishi M., Xu M., Wang Y., Chiang K.-J., Han J., Jung T.-P. (2020). Questionable Classification Accuracy Reported in “Designing a Sum of Squared Correlations Framework for Enhancing SSVEP-Based BCIs. IEEE Trans. Neural Syst. Rehabil. Eng..

[27] Oner M.U., Cheng Y.-C., Lee H.K., Sung W.-K. (2020).

[28] Poldrack R.A., Huckins G., Varoquaux G. (2020). Establishment of Best Practices for Evidence for Prediction A Review. JAMA Psychiatry.

[29] Ahmed H., Wilbur R.B., Bharadwaj H., Siskind J.M. (2021). Confounds in the Data—Comments on “Decoding Brain Representations by Multimodal Learning of Neural Activity and Visual Features”. IEEE Trans. Pattern Anal. Mach. Intell..

[30] Li R., Johansen J.S., Ahmed H., Ilyevsky T.V., Wilbur R.B., Bharadwaj H.M., Siskind J.M. (2021). The Perils and Pitfalls of Block Design for EEG Classification Experiments. IEEE Trans. Pattern Anal. Mach. Intell..

[31] Lyu Y., Li H., Sayagh M., (Jack) Jiang Z.M., Hassan A.E. (2021). An Empirical Study of the Impact of Data Splitting Decisions on the Performance of AIOps Solutions. ACM Trans. Software Eng. Method..

[32] Filho A.C., Batista A.F.D.M., dos Santos H.G. (2021). Data Leakage in Health Outcomes Prediction With Machine Learning. Comment on “Prediction of Incident Hypertension Within the Next Year: Prospective Study Using Statewide Electronic Health Records and Machine Learning. J. Med. Internet Res..

[33] Roberts M., Driggs D., Thorpe M., Gilbey J., Yeung M., Ursprung S., Aviles-Rivero A.I., Etmann C., McCague C., Beer L., Weir-McCall J.R., Teng Z., Gkrania-Klotsas E., Rudd J.H.F., Sala E., Schönlieb C.-B. (2021). Common Pitfalls and Recommendations for Using Machine Learning to Detect and Prognosticate for COVID-19 Using Chest Radiographs and CT Scans. Nat. Mach. Intell..

[34] Shim M., Lee S.-H., Hwang H.-J. (2021). Inflated Prediction Accuracy of Neuropsychiatric Biomarkers Caused by Data Leakage in Feature Selection. Sci. Rep..

[35] Vandewiele G., Dehaene I., Kovács G., Sterckx L., Janssens O., Ongenae F., De Backere F., De Turck F., Roelens K., Decruyenaere J., Van Hoecke S., Demeester T. (2021). Overly Optimistic Prediction Results on Imbalanced Data: A Case Study of Flaws and Benefits When Applying over-Sampling. Artif. Intell. Med..

[36] Arp D., Quiring E., Pendlebury F., Warnecke A., Pierazzi F., Wressnegger C., Cavallaro L., Rieck K. (2022).

[37] Barnett E., Onete D., Salekin A., Faraone S.V. (2022). Genomic Machine Learning Meta-Regression: Insights on Associations of Study Features with Reported Model Performance; techreport. medRxiv.

[38] Mongan J., Moy L., Kahn C.E. (2020). Checklist for Artificial Intelligence in Medical Imaging (CLAIM): A Guide for Authors and Reviewers. Radiology: Artif. Intell..

[39] Collins G.S., Reitsma J.B., Altman D.G., Moons K.G.M. (2015). Transparent reporting of a multivariable prediction model for individual prognosis or diagnosis (TRIPOD): the TRIPOD Statement. BMC Med..

[40] Mitchell M., Wu S., Zaldivar A., Barnes P., Vasserman L., Hutchinson B., Spitzer E., Raji I.D., Gebru T. (2019).

[41] Athey S., Imbens G.W. (2019). Machine Learning Methods That Economists Should Know About. Annu. Rev. Econom..

[42] Schrider D.R., Kern A.D. (2018). Supervised Machine Learning for Population Genetics: A New Paradigm. Trends Genet..

[43] Valletta J.J., Torney C., Kings M., Thornton A., Madden J. (2017). Applications of machine learning in animal behaviour studies. Anim. Behav..

[44] Iniesta R., Stahl D., McGuffin P. (2016). Machine learning, statistical learning and the future of biological research in psychiatry. Psychol. Med..

[45] Tonidandel S., King E.B., Cortina J.M. (2018). Big Data Methods: Leveraging Modern Data Analytic Techniques to Build Organizational Science. Organ. Res. Methods.

[46] Yarkoni T., Westfall J. (2017). Choosing Prediction Over Explanation in Psychology: Lessons From Machine Learning. Perspect. Psychol. Sci..

[47] Hofman J.M., Watts D.J., Athey S., Garip F., Griffiths T.L., Kleinberg J., Margetts H., Mullainathan S., Salganik M.J., Vazire S. (2021). Integrating explanation and prediction in computational social science. Nature.

[48] McDermott M.B.A., Wang S., Marinsek N., Ranganath R., Foschini L., Ghassemi M. (2021). Reproducibility in machine learning for health research: Still a ways to go. Sci. Transl. Med..

[49] Salganik M.J. (2020). Measuring the predictability of life outcomes with a scientific mass collaboration. Proc. Natl. Acad. Sci. USA.

[50] Stodden V., Seiler J., Ma Z. (2018). An empirical analysis of journal policy effectiveness for computational reproducibility. Proc. Natl. Acad. Sci. USA.

[51] Seibold H., Czerny S., Decke S., Dieterle R., Eder T., Fohr S., Hahn N., Hartmann R., Heindl C., Kopper P. (2021). A computational reproducibility study of PLOS ONE articles featuring longitudinal data analyses. PLoS One.

[52] Geirhos R., Jacobsen J.-H., Michaelis C., Zemel R., Brendel W., Bethge M., Wichmann F.A. (2020). Shortcut learning in deep neural networks. Nat. Mach. Intell..

[53] Chouldechova A., Benavides-Prado D., Fialko O., Vaithianathan R. (2018). In Proceedings of the 1st Conference on Fairness, Accountability and Transparency.

[54] Kuhn M., Johnson K. (2013).

[55] Filho A.C., Batista A.F.D.M., Santos H.G.d. (2021). Data Leakage in Health Outcomes Prediction With Machine Learning. Comment on “Prediction of Incident Hypertension Within the Next Year: Prospective Study Using Statewide Electronic Health Records and Machine Learning”. J. Med. Internet Res..

[56] Oner M.U., Cheng Y.-C., Lee H.K., Sung W.-K. (2020).

[57] Whalen S., Schreiber J., Noble W.S., Pollard K.S. (2022). Navigating the pitfalls of applying machine learning in genomics. Nat. Rev. Genet..

[58] Roberts D.R., Bahn V., Ciuti S., Boyce M.S., Elith J., Guillera-Arroita G., Hauenstein S., Lahoz-Monfort J.J., Schröder B., Thuiller W. (2017). Cross-validation strategies for data with temporal, spatial, hierarchical, or phylogenetic structure. Ecography.

[59] Valavi R., Elith J., Lahoz-Monfort J., Guillera-Arroita G. (2021).

[60] Malik M.M.A. (2020). Hierarchy of Limitations in Machine Learning. arXiv.

[61] Bone D., Goodwin M.S., Black M.P., Lee C.-C., Audhkhasi K., Narayanan S. (2015). Applying Machine Learning to Facilitate Autism Diagnostics: Pitfalls and Promises. J. Autism Dev. Disord..

[62] Zech J.R., Badgeley M.A., Liu M., Costa A.B., Titano J.J., Oermann E.K. (2018). Variable generalization performance of a deep learning model to detect pneumonia in chest radiographs: A cross-sectional study. PLoS Med..

[63] Szegedy C., Zaremba W., Sutskever I., Bruna J., Erhan D., Goodfellow I., Fergus R. (2014). Intriguing properties of neural networks. Proceedings of the International Conference on Learning Representations.

[64] Geirhos R., Rubisch P., Michaelis C., Bethge M., Wichmann F.A., Brendel W. (2018). Proceedings of the International Conference on Learning Representations.

[65] Carter B., Jain S., Mueller J., Gifford D. (2021). Overinterpretation reveals image classification model pathologies. Adv. Neural Inf. Process. Syst..

[66] Recht B., Roelofs R., Schmidt L., Shankar V. (2019). In Proceedings of the 36th International Conference on Machine Learning.

[67] Paullada, A.; Raji, I. D.; Bender, E. M.; Denton, E.; Hanna, A. Data and its (dis) contents: A survey of dataset development and use in machine learning research.Preprint at arXiv https://doi.org/10.1016/j.patter.2021.100336preprint arXiv:2012.05345 2020. 10.1016/j.patter.2021.100336 PMC8600147 34820643

[68] Scheuerman M.K., Hanna A., Denton E. (2021). Do Datasets Have Politics? Disciplinary Values in Computer Vision Dataset Development. Proc. ACM Hum. Comput. Interact..

[69] Gebru T., Morgenstern J., Vecchione B., Vaughan J.W., Wallach H., Iii H.D., Crawford K., Crawford K. (2021). Datasheets for datasets. Commun. ACM.

[70] Han S., Olonisakin T.F., Pribis J.P., Zupetic J., Yoon J.H., Holleran K.M., Jeong K., Shaikh N., Rubio D.M., Lee J.S. (2017). A checklist is associated with increased quality of reporting preclinical biomedical research: A systematic review. PLoS One.

[71] Garbin C., Marques O. (2022). Assessing Methods and Tools to Improve Reporting, Increase Transparency, and Reduce Failures in Machine Learning Applications in Health Care. Radiol. Artif. Intell..

[72] Raji D., Denton E., Bender E.M., Hanna A., Paullada A. (2021). AI and the Everything in the Whole Wide World Benchmark. Proceedings of the Neural Information Processing Systems Track on Datasets and Benchmarks.

[73] Lundberg I., Johnson R., Stewart B.M. (2021). What Is Your Estimand? Defining the Target Quantity Connects Statistical Evidence to Theory. Am. Socio. Rev..

[74] Liu D.M., Salganik M.J. (2019). Successes and Struggles with Computational Reproducibility: Lessons from the Fragile Families Challenge. Socius..

[75] Muchlinski D., Siroky D., He J., Kocher M. (2016). Comparing Random Forest with Logistic Regression for Predicting Class-Imbalanced CivilWar Onset Data. Polit. Anal..

[76] Colaresi M., Mahmood Z. (2017). Do the robot: Lessons from machine learning to improve conflict forecasting. J. Peace Res..

[77] Wang, Y. Comparing Random Forest with Logistic Regression for Predicting Class-Imbalanced Civil War Onset Data: A Comment. Political Analysis 2019, 27, Publisher: Cambridge University Press, 107–110.

[78] Kaufman A.R., Kraft P., Sen M. (2019).

[79] Bara C. (2020).

[80] (2020). http://archive.today/oUs4K.

[81] Ward M.D., Greenhill B.D., Bakke K.M. (2010). The perils of policy by p-value: Predicting civil conflicts. J. Peace Res..

[82] Breiman L. (2001). Statistical Modeling: The Two Cultures (with comments and a rejoinder by the author). Stat. Sci..

[83] Dressel J., Farid H. (2018). The accuracy, fairness, and limits of predicting recidivism. Sci. Adv..

[84] Olson R.S., Cava W.L., Mustahsan Z., Varik A., Moore J.H. (2018). Data-driven advice for applying machine learning to bioinformatics problems. Pacific Symposium on Biocomputing. Pac. Symp. Biocomput..

[85] Gorman K., Bedrick S. (2019). In Proceedings of the 57th Annual Meeting of the Association for Computational Linguistics.

[86] Blair R.A., Sambanis N. (2020). Forecasting Civil Wars: Theory and Structure in an Age of “Big Data” and Machine Learning. J. Conflict Resolut..

[87] Robin X., Turck N., Hainard A., Tiberti N., Lisacek F., Sanchez J.-C., Müller M. (2011). pROC: an open-source package for R and S+ to analyze and compare ROC curves. BMC Bioinf..

[88] He K., Zhang X., Ren S., Sun J. (2015). Delving Deep into Rectifiers: Surpassing Human-Level Performance on ImageNet Classification. CoRR.

[89] Szeliski R. (2021). https://szeliski.org/Book.

[90] Shi L., Lin L. (2019). The trim-and-fill method for publication bias: practical guidelines and recommendations based on a large database of meta-analyses. Medicine.

[91] Gurevitch J., Koricheva J., Nakagawa S., Stewart G. (2018). Meta-analysis and the science of research synthesis. Nature.

[92] Hofman J.M., Sharma A., Watts D.J. (2017). Prediction and explanation in social systems. Science.

[93] Islam R., Henderson P., Gomrokchi M., Precup D. (2017).

[94] Lones, M. A. How to avoid machine learning pitfalls: a guide for academic researchers.Preprint at arXiv:2108.02497 [cs] 2021,https://doi.org/10.48550/arXiv.2108.02497 arXiv: 2108.02497.

[95] Russakovsky O., Deng J., Su H., Krause J., Satheesh S., Ma S., Huang Z., Karpathy A., Khosla A., Bernstein M. (2015). ImageNet Large Scale Visual Recognition Challenge. Int. J. Comput. Vis..

[96] Koh P.W. (2021). In Proceedings of the 38th International Conference on Machine Learning.

[97] Rocca R., Yarkoni T. (2021). Putting Psychology to the Test: Rethinking Model Evaluation Through Benchmarking and Prediction. Advances in Methods and Practices in Psychological Science.

[98] Donoho D. (2017). 50 Years of Data Science. J. Comput. Graph Stat..

[99] Marie B., Fujita A., Rubino R. (2021). Proceedings of the 59th Annual Meeting of the Association for Computational Linguistics and the 11th International Joint Conference on Natural Language Processing.

[100] Clyburne-Sherin A., Fei X., Green S.A. (2019). Computational reproducibility via containers in social psychology. Meta-Psychology.

[101] (2018). Easing the burden of code review. Nat. Methods.

[102] Hutson M. (2019). No coding required: Companies make it easier than ever for scientists to use artificial intelligence. Science.

[103] Kapoor S., Narayanan A. (2021). https://www.codeocean.com/,%20version%20v1.

[104] Hook D.W., Porter S.J., Herzog C. (2018). Dimensions: Building Context for Search and Evaluation. Frontiers in Research Metrics and Analytics.

