# Supplementary material for: Leakage and the reproducibility crisis in machine-learning-based science
Source: Patterns (N Y). 2023 Aug 4;4(9):100804. doi: 10.1016/j.patter.2023.100804 (PMC10499856; doi:10.1016/j.patter.2023.100804)
Supplement: Document S1. Supplemental experimental procedures, Figures S1–S3, and Tables S1–S6 [file mmc1.pdf]

Patterns, Volume *4*

Supplemental information

Leakage and the reproducibility crisis in machine-learning-based science

Sayash Kapoor and Arvind Narayanan

Supplementary Information: Leakage and the Reproducibility Crisis in ML-based Science

Supplemental Experimental Procedures.

Overview of the Appendix. In Section S1, we justify our choice of the word reproducibility. In Section S2, we provide a detailed description of the methods we used to select papers for our review of civil war prediction and fix reproducibility issues in the papers with errors. In Section S3, we show how model info sheets address each type of leakage identified in our survey. In Section S4, we include a template for the model info sheets.

We include a list of all 124 papers that we considered for our literature review on civil war prediction as supplementary documents with this submission.

S1 Why do we call these reproducibility issues?

We acknowledge that there isn't consensus about the term reproducibility, and there have been a number of recent attempts to define the term and create consensus[1]. One possible definition is computational reproducibility—when the results in a paper can be replicated using the exact code and dataset provided by the authors[2]. We argue that this definition is too narrow because even cases of outright bugs in the code would not be considered irreproducible under this definition. Therefore we advocate for a standard where bugs and other errors in data analysis that change or challenge a paper's findings constitute irreproducibility.

The goal of predictive modeling is to estimate (and improve) the accuracy of predictions that one might make in a real-world scenario. This is true regardless of the specific research question one wishes to study by building a predictive model. In practice one sets up the data analysis to mimic this real-world scenario as closely as possible. There are limits to how well we can do this and consequently, there is always methodological debate on some issues, but there are also some clear rules. If an analysis choice can be shown to lead to incorrect estimates of predictive accuracy, there is usually consensus in the ML community that it is an error. For example, violating the train-test split (or the learn-predict separation) is an error because the test set is intended to provide an accurate estimate of 'out-of-sample' performance—model performance on a dataset that was not used for training[3]. Thus, to define what is an error, we look to this consensus in the ML community (e.g. in textbooks) and offer our own arguments when necessary.

S2 Materials and Methods: Reproducibility issues in civil war prediction

Different researchers might have different aims when comparing the performance on civil war prediction — determining the absolute performance, or comparing the relative performance of different models of civil war prediction. Whether the aim is to determine the relative or absolute performance of models of civil war prediction, data leakage causes a deeper issue in the findings of each of the 4 papers with errors that leads to inaccurate estimates of both relative and absolute out-of-sample performance.

In correcting the papers with errors[4–7], our aim is to report out-of-sample performance of the various models of civil war prediction after correcting the data leakage, while keeping all other factors as close to the original implementation as possible. Fixing the errors allows a more accurate estimate of out-of-sample performance.

At the same time, we caution that just because our corrected results offer a more accurate estimate of out-of-sample performance doesn't mean that we endorse all other methodological choices made in the papers. For example, to correct the results reported by Muchlinski et al.[4], we use imputation on an out-of-sample dataset that has 95% missing values. While an imputation model created only using the training data avoids data leakage, it does not mean that using a dataset with 95% missing values to measure out-of-sample performance is desirable.

S2.1 Paper selection for review

To find relevant papers on civil war prediction for our review, we used the search results from a dataset of academic literature[8] for papers with the terms *'civil' AND 'war' AND ('prediction' OR 'predicting' OR 'forecast')* in their title or abstract, as well as papers that were cited in a recent review of the field[9]. To keep the number of papers tractable, we limited ourselves to those that were published in the last 5 years, specifically, papers published between 1st January 2016 and 14th May 2021. This yielded 124 papers. We narrowed this list to the 15 papers that were focused on predicting civil war and evaluated performance using a train-test split. Of the 15 papers that meet our inclusion criteria, 12 share the complete code and data. For these 12, we attempted to identify errors and reproducibility issues from the text and through reviewing the code provided with the papers. When we identified errors, we re-analyzed the data with the errors corrected. We now address the reproducibility issues we found in each paper in detail.

S2.2 Muchlinski et al.[4]

Imputation is commonly used to fill in missing values in datasets[10]. Imputing the training and test datasets together refers to using data from the training as well as the test datasets to create an imputation model that fills in all missing values in the dataset. This is an erroneous imputation method for the predictive modeling paradigm, since it can lead to data leakage, which results in incorrect, over-optimistic performance claims. This pitfall is well known in the predictive modeling community — discussed in ML textbooks[3], blogs[11] and popular online forums[12].

Muchlinski et al.[4] claim that a Random Forests model vastly outperforms logistic regression models in terms of out-of-sample performance using the AUC metric[13]. However, since they impute the training and test datasets together, their results suffer from data leakage. The impact of leakage is especially severe because of the level of missingness in their out-of-sample test dataset: over 95% of the values are missing (which is not reported in the paper), and 70 of the 90 variables used in their model are missing for *all* instances in the out-of-sample test set.[1] When their imputation method is corrected, their Random Forests model performs no better than the logistic regression models that they compared against.

We focus on reproducing the out-of-sample results reported by Muchlinski et al.[4]. Table S1 provides the comparisons between the results reported in Muchlinski et al.[4], our reproductions of their reported (incorrect) results, as well as the corrected version of their results. Muchlinski et al.[4] received two critiques of the methods used in their paper[6,14]. [2]. In response, they published a reply with clarifications and revised code addressing both critiques[16]. We use the revised version of their code. We find that the error in their imputation methods exists in the revised code as well as the original code, and was not identified by the previous critiques. Muchlinski et al.[4] re-use the dataset from Hegre and Sambanis[17] when training their models, and provide a separate out-of-sample test set for evaluation. To address missing values, they use a Random Forests based imputation method in R called *rfImpute*. However, the training and test sets are imputed together, which leads to a data leakage. This results in overoptimistic performance claims. Below, we detail the steps we take to correct their results, provide a visualization of the data leakage, and provide a simulation showcasing how the data leakage can result in overoptimistic claims of performance.

Correcting the data imputation. To correct this error, we use the *mice* package in R which uses multiple imputation for imputing missing data. This is because the *mice* package allows us to specify which rows in the dataset are a part of the test set and it does not use those rows for creating the imputation

[1]While leakage is particularly serious in predictive modeling, a dataset with 95% of values missing is problematic even for explanatory modeling.

[2]Hofman et al.[15] also outline the shortcomings in the initial code released by Muchlinski et al.[4].

model, whereas *rfImpute* — the original method used to impute the missing data in the original results by Muchlinski et al.[4] — does not have this feature. The authors imputed the training set together with the out-of-sample test set using *rfImpute*, which led to data leakage. Table S1 provides the comparisons between the results reported in Muchlinski et al.[4], our reproductions of their reported (incorrect) results, as well as the corrected version of their results.

Using multiple imputation fills in missing values without regarding the underlying variable's original distribution. For example, using multiple imputation fills in different missing values for the variable representing the percentage of rough terrain in a country in different years[18], whereas this particular variable (percentage of rough terrain) is constant over time. However, when multiple imputation is used with a train-test split, there is still no leakage between the training and test sets, since the imputation model only uses data from the training set to fill in missing values in the test set.

Why can't we use *rfImpute* in the corrected results? Instead of using the *mice* package, another way to impute the data correctly, i.e., without data leakage, would be to run the imputation using *rfImpute* on the training and test data separately — creating two separate imputation models — one for the training data and one for the test data. We could not use this imputation method because 70 of the 90 variables used in Muchlinski et al.[4]'s model as features do not have *any* values in the out-of-sample test data provided — i.e. they are missing for *all* observations in the out-of-sample dataset — and *rfImpute* requires at least some values for each variable to not be missing. In other words, the *mice* package allows us to train an imputation model on the training set and use it to fill in missing values in the test set.

Subtle differences between explanatory and predictive modeling. In the explanatory modeling paradigm, the aim is to draw inferences from data, as opposed to optimizing and evaluating out-of-sample predictive performance. In this case, data imputation would be considered a part of the data pre-processing step, even though it is still important to keep in mind the various assumptions being made in this process Schafer[19]. Contrarily, in the predictive modeling paradigm, the imputation is a part of the modeling step[3] because the aim of the modeling exercise is to validate performance on an out-of-sample test set, which the model does not have access to during the training. In this case, imputing the training and test datasets together leads to leaking information from the test set to the training set and thus the performance evaluation on the purportedly "out-of-sample" test set would be an over-estimate.

What is the precise mechanism by which the leakage occurs in Muchlinski et al.[4]? When Muchlinski et al.[4] impute the missing values in the out-of-sample test set, the imputation model has access to the entire training data as well as the labels of the target variables in the test data — they also include the target variable in the list of variables which the imputation model treats as independent variables when carrying out the imputation. The model therefore uses correlations between the target variable and independent variables in the training dataset and uses them to fill in the missing values in the test dataset — i.e. the model uses the labels of the target variables in the test data and correlations from the training data to fill in missing values. This leads to the test dataset having similar correlations between the target and independent variables as the ones present in the training data. Further, the missing data is filled in in such a way that it favors ML models such as Random Forests over logistic regression models, as we show in the visualization below.

Visualizing the leakage. We can visually observe an instance of data leakage in Figure S1. We focus on the distribution of the feature *agexp*, which represents the proportion of agricultural exports in the GDP of a country. We choose this feature because in the Muchlinski et al. paper, this feature had the highest gini index for the random forests model — which means that it was an important feature for the model. While we only visualize one feature here, similar results hold across multiple features used in the model. Below, we reconstruct the process by which the data leakage was generated — following the exact steps Muchlinski et al.[4] used to create and evaluate the dataset:

- Figure S1a represents the distribution of the *agexp* variable for war and peace data points in the original dataset by Hegre and Sambanis[17], ignoring missing values.

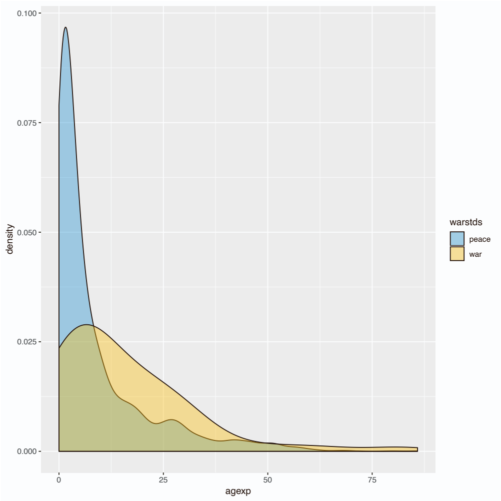

(a) Distribution of the *agexp* variable for peace and war data points for the original Hegre et al. dataset, ignoring missing values

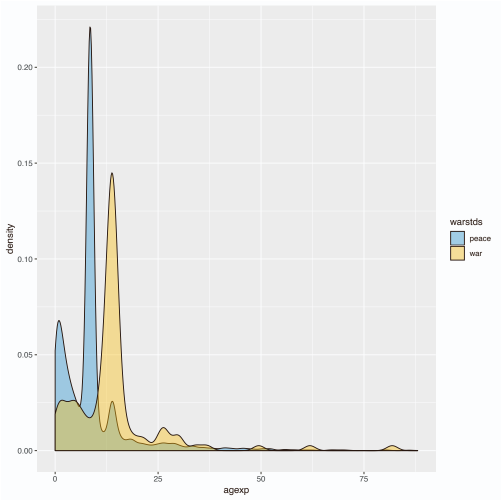

(b) Distribution of the *agexp* variable for peace and war data points for the imputed Hegre et al. dataset used by Muchlinski et al. for training

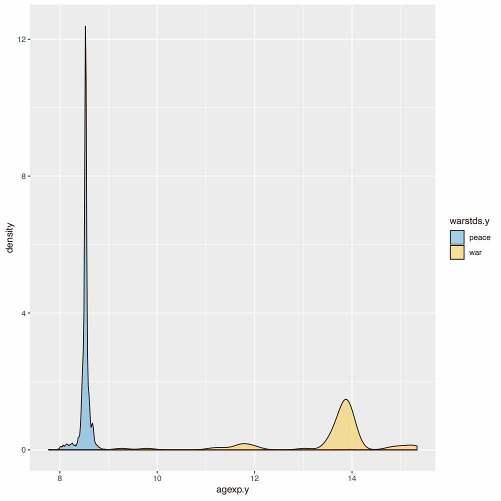

(c) Distribution of the *agexp* variable for peace and war data points only for the data points that were added during imputation (i.e. the data points that were missing in the original dataset)

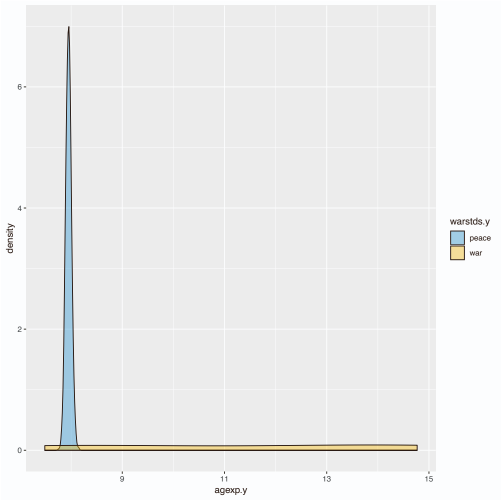

(d) Distribution of the *agexp* variable for peace and war data points for the out-of-sample test set

Figure S1: Distribution of the *agexp* variable for peace and war data points for different imputation steps in Muchlinski et al.[4]. Note that the distribution of *peace* instances in the test set (D) has a peak that is close to the distribution in the imputed training set (B, C) — which allows the random forests model to learn the small range of values where *peace* data points are concentrated. While we report results for the *agexp* variable, similar trends appear across independent variables in the dataset.

- Figure S1b shows the same distribution after including the imputed values of *agexp*. In particular, we see two peaks in the dataset for war and peace data points alike, one due to war instances and one due to peace instances.
- If we look only at the data points that were imputed using the *rfImpute* method (Figure S1c), we see that the distribution of the imputed data points for war and peace are completely separated, in contrast to the original distribution where there was a significant overlap between the distributions.
- Finally, Figure S1d shows the effect of imputing this already-imputed dataset with the out-of-sample test set — we see that the out-of-sample dataset only has the peak for peace datapoints, whereas the distribution for war is almost uniform.

Further, the random forests model can learn the peak for the *agexp* variable in the *peace* instances from the training dataset after imputation, since the peak for the training and test sets is similar. It can distinguish between war and peace datapoints much more easily compared to a logistic regression model that only uses one parameter per feature — logistic regression models are monotonic functions of the independent variables and therefore cannot learn that a variable only lies within a small range for a given label. This highlights the reason behind Random Forests outperforming logistic regression in this setting — imputing the training and test datasets together leads to variable values being artifically concentrated within a very small range for both the training and test datasets — and further, being neatly separated across *war* and *peace* instances. The impact of the imputation becomes even clearer when we consider that the out-of-sample test dataset provided by Muchlinski et al.[4] has over 95% of the data missing, and 70 out of 90 variables are missing for all instances in the out-of-sample dataset.

A simulation showcasing the impact of missingness on performance estimates in the presence of leakage. We can observe a visual example of how data leakage affects performance evaluation in Figure S2. We describe the simulation below:

- there are two variables — the target variable *onset* and the independent variable *gdp*.
- *onset* is a binary variable. *gdp* is drawn from a normal distribution and depends on *onset* as follows:

$$gdp = N(0, 1) + onset.$$

- We generate 1000 samples with *onset=0* and 1000 samples with *onset=1* to create the dataset.
- We randomly split the data into training (50%) and test (50%) sets, and create a random forests model that is trained on the training set and evaluated on the test set.
- To observe the impact of imputing the training and test sets together, we randomly delete a certain percentage of values of *gdp*, and impute it using the imputation method used in Muchlinski et al.[4].
- We vary the proportion of missing values from 0% to 95% in increments of 5% and plot the accuracy of the random forests classifier on the test set.
- We run the entire process 100 times and report the mean and 95% CI of the accuracy in Figure S2; the 95% CI is too small to be seen in the Figure.

We find that imputing the training and test sets together leads to an increasing improvement in the purportedly "out-of-sample" accuracy of the model. Estimates of model performance in this case are artificially high. This example also highlights the impact of the high percentage of missing values — since the out-of-sample test set used by Muchlinski et al.[4] contains over 95% missing values, the impact of imputing the training and test sets together is very high.

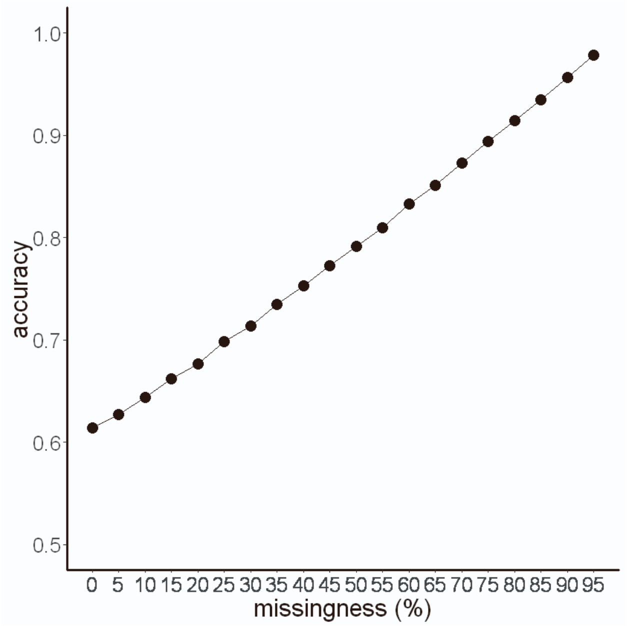

Figure S2: Results of a simulation that showcase how imputing the training and test sets together leads to overoptimistic estimates of model performance. The 95% Confidence Intervals are too small to be seen.

Algorithm	Reported	Reported results (reproduced)	Corrected results
Fearon and Laitin	0.69	0.78	0.54
Collier and Hoeffler	0.90	0.83	0.57
Hegre and Sambanis	0.83	0.82	0.68
Muchlinski et al.	0.94	0.95	0.64

Table S1: Original and corrected results in Muchlinski et al.[4]. While there are differences between the reported results and our reproduction of the reported results, especially for the Fearon and Laitin as well as the Collier and Hoeffler models, the relative order of the model performance for both results is the same.

S2.3 Colaresi and Mahmood[5]

Colaresi and Mahmood[5] report that ML models vastly outperform logistic regression for predicting civil war onset. However, they re-use the imputed version of the dataset in Hegre and Sambanis[17] which is provided by Muchlinski et al.[4]. They use the imputed dataset both for training and testing via a train-test split; they do not use the out-of-sample test set provided by Muchlinski et al. This means that the results in Colaresi and Mahmood[5] are subject to exactly the same pitfall as in Muchlinski et al.[4], albeit with a slightly different dataset. Correcting the imputation method dramatically reduces the performance of the ML models proposed.

We focus on reproducing the final round of results reported in the paper Colaresi and Mahmood[5], which consists of a comparison of 3 models of civil war onset — the Random Forests model proposed in Muchlinski et al.[4], the Random Forests model proposed in Colaresi and Mahmood[5] as well as the logistic regression model proposed in Fearon and Laitin[20]. Their dataset has 17.4% values missing, and the test set has 19% values missing. The proportion of missing values in individual variables can be even higher — for example, the *agexp*, which represents the proportion of agricultural exports in the GDP of a country, is missing for 54.3% of the rows in the test set. In our corrected results, we use the original dataset from Hegre and Sambanis[17] and impute the training and test data separately using the *rfImpute* function. The test set consists of data from the years after 1988. One of the independent variables, *milper*, is missing for all instances in the test set of Colaresi and Mahmood[5] so we exclude this variable from our models. Table S2 provides the comparisons between the results reported in Colaresi and Mahmood[5], our reproductions of their

Algorithm	Reported	Reported results (reproduced)	Corrected results
Fearon and Laitin	0.77	0.77	0.79
Muchlinski et al.	0.89	0.89	0.73
Colaresi and Mahmood	0.91	0.91	0.75

Table S2: Original results from Colaresi and Mahmood[5] and our corrected results.

reported (incorrect) results, as well as the corrected version of their results.

Colaresi and Mahmood[5] and Wang[6] reuse the dataset released by Muchlinski et al.[4]. This is the imputed version of the dataset released by Hegre and Sambanis[17]. However, for 777 rows in the imputed dataset released by Muchlinski et al.[4], the original dataset by Hegre and Sambanis[17] has a missing target variable (i.e. the variable representing civil war onset is missing) whereas the imputed version of the dataset (i.e. the dataset released by Muchlinski et al.[4]) has a value of *peace* for the target variable representing civil war onset. Since Muchlinski et al.[4] do not share the code that they use for imputing the Hegre and Sambanis[17] dataset, it is unclear how the missing values in the target variable were imputed in the dataset, especially since the imputation method they use — *rfImpute* — requires non-missing values in the target variable. Still, the number of instances of civil war onset (i.e. instances where the variable representing civil war onset has the value *war*) in the Hegre and Sambanis[17] dataset as well as the Muchlinski et al.[4] dataset are the same.

S2.4 Wang[6]

Similar to Colaresi and Mahmood[5], Wang[6] report that ML models vastly outperform logistic regression for predicting civil war onset. However, they too re-use the imputed version of the dataset in Hegre and Sambanis[17] provided by Muchlinski et al.[4]. They use the imputed dataset both for training and testing via k-fold cross-validation; they do not use the out-of-sample test set provided by Muchlinski et al. Correcting the imputation method dramatically reduces the performance of the ML models proposed.

We focus on reproducing the results of the nested cross-validation implementation reported by Wang[6]. Wang[6] reuses the imputed dataset provided by Muchlinski et al.[4], instead of using the original dataset provided by Hegre and Sambanis[17] and imputing the training and test sets separately. The dataset has 17.4% values missing. The proportion of missing values in individual variables can be even higher — for example, the *agexp*, which represents the proportion of agricultural exports in the GDP of a country, is missing for 49.8% of the rows in the data set. In our corrected results, we use the original dataset from Hegre and Sambanis[17] and impute the training and test data separately using the *rfImpute* function within each cross validation fold. This ensures that there is no data leakage between the training and test sets in each fold. Table S3 provides the comparisons between the results reported in Wang[6], our reproductions of their reported (incorrect) results, as well as the corrected version of their results.

We also conduct an additional robustness analysis in which we use a separate out-of-sample test set instead of $k-$fold cross validation, since using $k-$fold cross validation with temporal data can also lead to leakage across the train-test split. To maintain comparability between the original and corrected results by testing on the same instances of civil war, we continue to use $k-$fold cross-validation in the corrected results in Figure 2. We report the results after making this change in Table S3. We use the same train-test split as Colaresi and Mahmood[5] — $year < 1988$ as training data and the rest as test data — for the out-of-sample test set. The test set consists of data from the years after 1988. One of the independent variables, *milper*, is missing for all instances in the test set of Colaresi and Mahmood[5] so we exclude this variable from our models.

Note that the imputation method that should be used depends on the exact model deployment scenario, and should mimic it as closely as possible for accurate performance estimates. For example, in some model deployment settings samples for prediction come in one at a time and in some cases they come in batches. In the former setting, imputing the entire test set together may result in overoptimistic performance evaluations as well, since the deployed model doesn't have access to a batch of samples. Our results may thus offer an upper bound on the performance of civil war prediction models in the case of Colaresi and Mahmood[5] and Wang[6].

Algorithm	Reported	Reported (reproduced)	k-fold CV (corrected)	Out-of-sample (corrected)
Fearon and Laitin	0.76	0.76	0.77	0.78
Collier and Hoeffler	0.78	0.78	0.72	0.77
Hegre and Sambanis	0.80	0.80	0.81	0.80
Muchlinski et al.	0.92	0.92	0.78	0.73
AdaBoost	0.94*	0.94	0.82	0.77
GBT	0.94*	0.94	0.81	0.75

Table S3: Original and corrected results in the Wang[6]. We find that using an out-of-sample test set further favors logistic regression models over ML models. The metric for all results is AUC. *These results were not reported using nested cross-validation in Wang[6]. In our reproduction of these reported results, we use nested cross-validation, which ensures that we do not get over-estimates of performance.

S2.5 Kaufman, Kraft, and Sen[7]

We focus on reproducing the results on civil war prediction in Kaufman, Kraft, and Sen[7]. There are several issues in the paper's results. We outline each issue below and provide a comparison of various scenarios in Table S4 that highlight the precise cause of the performance difference between the original and corrected results, and visualize the robustness of our corrected results. We find that even though there are several issues in Kaufman, Kraft, and Sen[7], the main difference in performance between the original results they report and our corrected results is due to data leakage.

Data leakage due to proxy variables. The dataset used by Kaufman, Kraft, and Sen[7] has several variables that, if used as independent variables in models of civil war prediction, could cause data leakage, since they are proxies of the outcome variable. Table S5 lists the variables in the Fearon and Laitin[20] dataset that cause leakage. The first 4 rows outline variables that could be affected by civil wars, as outlined in Fearon and Laitin[20]. Therefore, following Fearon and Laitin[20], we use lagged versions of these variables in our correction. The other variables in Table S5 are either direct proxies of outcomes of interest or are missing for all instances for civil war.

Parameter selection for the Lasso model. Kaufman, Kraft, and Sen[7] use an incorrect parameter selection technique when creating their Lasso model that leads to the model always predicting *peace* (i.e. all coefficients of the variables in the model are always zero). We correct this using a standard technique for parameter selection. Instead of choosing model parameters such that the model always predicts *peace*, we use the *cv.glmnet* function in R to choose a suitable value for model parameters based on the training data.

Using $k-$fold cross-validation with temporal data. $k-$fold cross-validation shuffles the dataset before it is divided into training and test datasets. When the dataset contains temporal data, the training dataset could contain data from a later date than the test dataset because of being shuffled. To maintain comparability between the original and corrected results by testing on the same instances of civil war, we continue to use $k-$fold cross-validation in the corrected results in Figure 2. To evaluate out-of-sample performance without using cross-validation, we use a separate train-test split instead of $k-$fold cross-validation and report the difference in results for this scenario in the row *Corrected (out-of-sample)* in Table S4. We find that there is no substantial difference between the results when using the out-of-sample test set and $k-$fold cross-validation — in each case, none of the models outperforms a baseline that predicts the outcome of the previous year. We use the same train-test split as Colaresi and Mahmood[5] — $year < 1988$ as training data and the rest as test data.

Replacing missing values with zeros. Kaufman, Kraft, and Sen[7] replace missing values in their dataset with zeros, instead of imputing the missing data or removing the rows with missing values. This is a methodologically unsound way of dealing with missing data: for example, the models would not be able to discern whether a variable has a value of zero because of missing data or because it was the true value of the variable for that instance. This risks getting underestimates of performance, as opposed to overoptimistic

performance claims. As a robustness check, we impute the training and test data separately in each cross-validation fold using the *rfImpute* function in R and report the results in the *Corrected (imputation)* row of Table S4. We find that the choice of imputation method does not cause a difference in performance, perhaps because only 0.6% of the values of variables are missing in the dataset.

Choice of cut-offs for calculating accuracy. Instead of calculating model cutoffs based on the best cutoff in the training set, Kaufman et al. use the distribution of model scores to decide the cutoffs for calculating accuracy. We include robustness results when we change the cutoff selection procedure to choosing the best cutoffs for the training set in the *Corrected (cutoff choice)* row of Table S5. We find that the choice of cutoff does not impact the main claim — the performance of the best model is still worse than a baseline that predicts the outcome of the previous year.

Weak Baseline. Kaufman, Kraft, and Sen[7] compare their results against a baseline model that always predicts *peace*. We find that a baseline that predicts *war* if the outcome of the target variable was civil war in the previous year and predicts *peace* otherwise is a stronger baseline (Accuracy: 97.5% vs. 86.1%; χ^2=633.7, $p = 7.836e\text{-}140$ using McNemar's test as detailed in Dietterich[21]), and report results against this stronger baseline in Table S4.

Confusion about the target variable. Kaufman, Kraft, and Sen[7] use ongoing civil war instead of civil war onset as the target variable in their models. While their abstract mentions that the prediction task they attempt is civil war onset prediction, they switch to using the term *civil war incidence* in later sections, without formally defining this term. To attempt to determine what they mean by this term, we looked at the papers they cite; one of them has the term *civil war incidence* in the title Collier and Hoeffler[22], and defines civil war incidence as 'observations [that] experienced a start of a civil war'. At the same time, in the introduction, they state that they are 'predicting whether civil war occurs in a country in a given year' — which refers to ongoing civil war instead of civil war onset. This might confuse a reader about the specific prediction task they undertake.

Scenario	ADT	RF	SVM	ERF	Lasso	LR	Baseline	Stronger Baseline
Reported	0.990	0.989	0.983	0.990	0.862	0.987	0.861	0.000
Reported (reproduction)	0.990	0.990	0.983	0.989	0.861	0.987	0.861	0.000
Corrected	0.974	0.959	0.974	0.957	0.975	0.972	0.861	0.975
Corrected (out-of-sample)	0.966	0.936	0.962	0.927	0.966	0.963	0.796	0.966
Corrected (imputation)	0.974	0.959	0.974	0.957	0.975	0.975	0.861	0.975
Corrected (cutoff choice)	0.974	0.972	0.966	0.967	0.975	0.971	0.861	0.975

Table S4: Results for the various scenarios in Kaufman, Kraft, and Sen[7]. We report results up to 3 significant figures in this table because the small difference in performance between AdaBoost and logistic regression that is ascribed signifance in Kaufman, Kraft, and Sen[7] can only be observed in the third decimal point. The first 2 values of 'Stronger Baseline' are reported as 0 because this baseline was not included in the results of Kaufman, Kraft, and Sen[7].

S2.6 Blair and Sambanis[23]

Blair and Sambanis[23] state that their *escalation* model outperforms other models across a variety of settings. However, they do not test the performance evaluations to see if the difference is statistically significant. We find that there is no significant difference between the smoothed AUC values of the *escalation* model's performance and other models they compare it to when we use a test for significance. Further, we provide a visualization of the 95% confidence intervals of specificities and sensitivities in the smoothed ROC curve they report for their model (*escalation*) as well as for a baseline model (*cameo*) — and find that the 95% confidence intervals are large (see Figure S3).

Variable name	Reason for leakage	Variable definition in data documentation
pop	affected by target variable	population; in 1000s
lpop	affected by target variable	log of population
polity2	affected by target variable	revised polity score
gdpen	affected by target variable	gdp/pop based on pwt5.6; wdi2001;cow energy data
onset	codes civil war onset	1 for civil war onset
ethonset	codes civil war onset	1 if onset = 1 and ethwar ~= 0
durest	NA if onset = 0	estimated war duration
aim	NA if onset = 0	1 = rebels aim at center; 3 = aim at exit or autonomy; 2 = mixed or ambig.
ended	NA if onset = 0	war ends = 1; 0 = ongoing
ethwar	NA if onset = 0	0 = not ethnic; 1 = ambig/mixed; 2 = ethnic
emponset	codes civil war onset	onset coded for data with empires
sdwars	codes ongoing civil war	Number of Sambanis/Doyle civ wars in progress
sdonset	codes civil war onset	onset of Sambanis/Doyle war
colwars	codes ongoing civil war	Number of Collier/Hoeffler wars in progress
colonset	codes civil war onset	onset of Collier/Hoeffler war
cowwars	codes ongoing civil war	Number of COW civ wars in progress
cowonset	codes civil war onset	onset of COW civ war

Table S5: This table highlights the variables included as independent variables in Kaufman, Kraft, and Sen[7] which cause a data leakage. In the original use of the dataset, Fearon and Laitin[20] include lagged versions of the first 4 variables in the list as independent variables in their model to avoid leakage. Following their use of lagged versions of these variables, we do the same in our correction to avoid leakage. The other variables are proxies for the outcomes of interest and hence we remove them from the models to avoid data leakage.

Uncertainty quantification, p-values and Z-values for tests of statistical significance.

- We report p-values and Z values for a one-tailed significance test comparing the smoothed AUC performance of the *escalation* model with other baseline models reported in their paper — *quad, goldstein, cameo* and *average* respectively. Note that we do not correct for multiple comparisons; such a correction would further reduce the significance of the results. We implement the comparison test for smoothed ROC curves detailed in Robin et al.[24].
  - 1 month forecasts: $Z = 0.64, 1.09, 0.42, 0.67$; $p = 0.26, 0.14, 0.34, 0.25$
  - 6 months forecasts: $Z = 0.41, 0.08, 0.70, 0.69$; $p = 0.34, 0.47, 0.24, 0.25$
- The 95% confidence intervals for the 1 month models are:
  - *escalation*: 0.66-0.95
  - *quad*: 0.63-0.95
  - *goldstein*: 0.62-0.93
  - *cameo*: 0.65-0.95
  - *average*: 0.65-0.95
- The 95% confidence intervals for the 6 month models are:
  - *escalation*: 0.64-0.93
  - *quad*: 0.60-0.90
  - *goldstein*: 0.68-0.93
  - *cameo*: 0.58-0.92
  - *average*: 0.60-0.92

While a small p-value is used to reject the null hypothesis (in this case — that the out-of-sample performance does not differ between the models being compared), a singular focus on a test for statistical significance at a pre-defined threshold can be harmful (see, for example Imbens[25]). Blair and Sambanis do report performance evaluations for a variety of different model specifications. However, the purpose of such

robustness checks is to determine whether model performance sensitive to the parameter choices; it is unclear whether it helps deal with issues arising from sampling variance. At any rate, Blair and Sambanis's results turn out to be highly sensitive to another modeling choice: the fact that they compute the AUC metric on the smoothed ROC curve instead of the empirical curve that their model produces. Smoothing refers to a transformation of the ROC curve to make the predicted probabilities for the war and peace instances normally distributed instead of using the empirical ROC curve (see Robin et al.[24]). This issue was pointed out by Beger, Morgan, and Ward[26] and completely changes their original results; Blair and Sambanis[27] discuss it in their rebuttal.

S2.7 Overview of papers in Table S6

Table S6 provides the list of 12 papers included in our review, showing information about whether they report confidence intervals, conduct tests of statistical significance when comparing classifier performance, which metrics they report, the number of rows and the number of positive instances (i.e. instances of war/conflict) in the test set, and whether their main claim relies on out-of-sample evaluation of classifier performance. We detail information about the numbers we report in Table S6 below.

- **Hegre et al.[28]**: We report the number of rows and number of positive instances of civil war incidence for the dates between 2001 and 2013 in the UCDP dataset, i.e. all years for which out-of-sample estimates are provided. We report the out-of-sample AUC performance difference for the Major conflict setting. Out-of-sample evaluation results are not included in the main text of the paper, hence we report that the paper's main claim does not rely on out-of-sample evaluations.

- **Muchlinski et al.[4]**: We report the number of rows and number of positive instances of civil war onset for the dates after 2000 in the out-of-sample dataset provided by Muchlinski et al. We report the out-of-sample AUC performance difference between the Random Forests and the best logistic regression setting. Out-of-sample evaluation results are used to justify the performance improvement of using Random Forests models, hence we report that the paper's main claim relies on out-of-sample evaluations.

- **Chiba and Gleditsch[29]**: We report the total number of instances and the number of positive instances of governmental onsets in the years 2013-14 (the test set dates). We report the difference between the territorial onset AUC's reported in the paper. Note that while Chiba and Gleditsch[29] do report small number of data points that are used in one of their settings, they do not address how to estimate variance or perform tests of statistical significance. Out-of-sample evaluation results are not used as the main evidence of better performance in the main text of the paper, hence we report that the paper's main claim does not rely on out-of-sample evaluations.

- **Colaresi and Mahmood[5]**: We report the number of rows and onsets of civil war after the year 1988 (the test set dates). We report the out-of-sample AUC difference between the two random forests models compared in the paper. Out-of-sample evaluation results are used to justify the performance improvement of using an iterative method for model improvement, hence we report that the paper's main claim relies on out-of-sample evaluations.

- **Hirose, Imai, and Lyall[30]**: We report the number of locations included in the out-of-sample results. Since the paper does not attempt binary classification, we do not report the number of positive instances in this case. We report the out-of-sample performance gain of adding relative ISAF support to the baseline model in the IED attack setting of the paper. Out-of-sample evaluation results are used as important evidence of better model performance in the main text of the paper, hence we report that the paper's main claim relies on out-of-sample evaluations.

- **Schutte[31]**: We report the number of rows in the entire dataset, since the paper uses k-fold cross validation and therefore all instances are used for testing. Since the paper does not attempt binary classification, we do not report the number of positive instances in this case. We report the out-of-sample normalized MAE difference between the population model and the best performing model compared in the paper. Out-of-sample evaluation results are used as important evidence of better

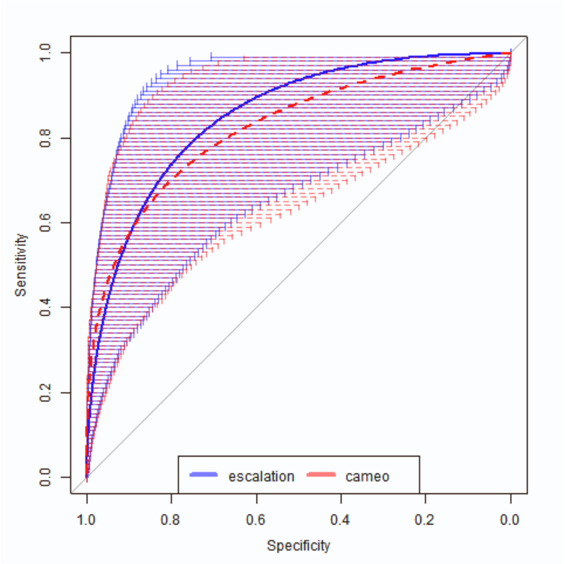

(a) Visualizing the 95% confidence intervals of the specificities for the 1 month forecast in the smoothed ROC curve reported in Blair and Sambanis[23].

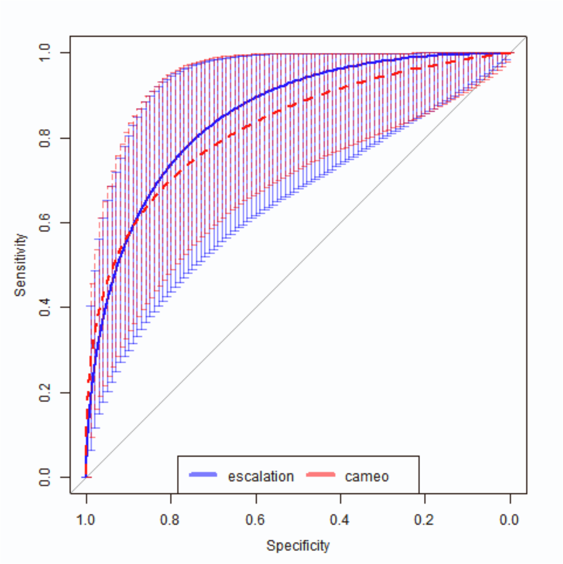

(b) Visualizing the 95% confidence intervals of the sensitivities for the 1 month forecast in the smoothed ROC curve reported in Blair and Sambanis[23].

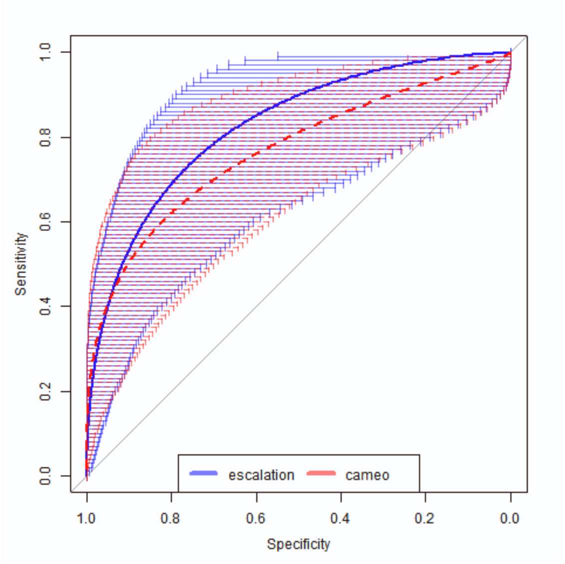

(c) Visualizing the 95% confidence intervals of the specificities for the 6 month forecast in the smoothed ROC curve reported in Blair and Sambanis[23].

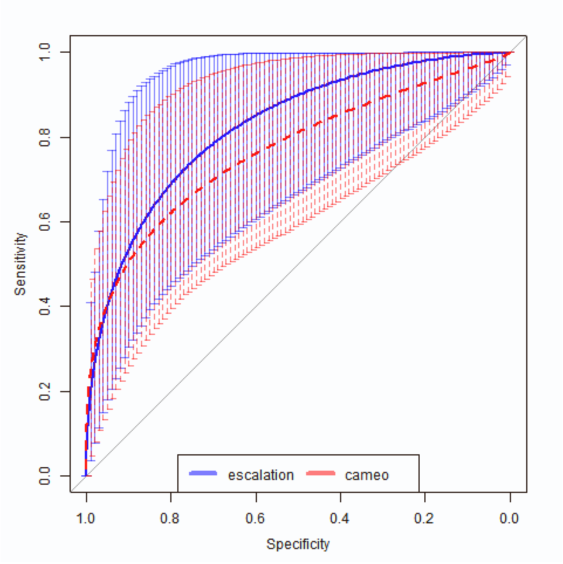

(d) Visualizing the 95% confidence intervals of the sensitivities for the 6 month forecast in the smoothed ROC curve reported in Blair and Sambanis[23].

Figure S3: The wide confidence intervals for sensitivities and specificities reported in Blair and Sambanis. Here, we visualize the *escalation* and *cameo* models for the 1 month and 6 month forecast in the base specification (reported in Figure 1 of their paper).

model performance in the main text of the paper, hence we report that the paper's main claim relies on out-of-sample evaluations.

- **Hegre, Hultman, and Nygård[32]**: We report the number of rows and number of positive instances of civil war incidence for the dates between 2001 and 2013 in the UCDP dataset, i.e. all years for which out-of-sample estimates are provided. We report the out-of-sample AUC performance difference for

the Major conflict setting. Out-of-sample evaluation results are not used as the primary evidence of better model performance in the main text of the paper, hence we report that the paper's main claim does not rely on out-of-sample evaluations.

- **Hegre et al.[33]**: We report the number instances with state based conflict in the ViEWS Monthly Outcomes at PRIO-Grid Level data between 2015 and 2017 — the years for which the out-of-sample results are reported in the paper. We report the out-of-sample AUC performance difference for the state-based conflict setting. Out-of-sample evaluation results are used as the primary evidence of better model performance in the main text of the paper, hence we report that the paper's main claim relies on out-of-sample evaluations.
- **Kaufman, Kraft, and Sen[7]**: We report the total number of rows and all instances of civil war incidence in the dataset used by Kaufman et al., since they use k-fold cross validation and therefore all instances are used for testing. We report the out-of-sample accuracy difference between the Adaboost and logistic regression settings. Out-of-sample evaluation results are used as the primary evidence of better model performance in the main text of the paper, hence we report that the paper's main claim relies on out-of-sample evaluations.
- **Wang[6]**: We report the total number of rows and onsets of civil war used in the dataset used by Wang since they use k-fold cross validation and therefore all instances are used for testing. We report the out-of-sample AUC performance difference between the Adaboost and logistic regression models. Out-of-sample evaluation results are used as the primary evidence of better model performance in the main text of the paper, hence we report that the paper's main claim relies on out-of-sample evaluations.
- **Blair and Sambanis[23]**: We report the number of rows and onsets of civil war after the year 2007 (the test set dates). We report the out-of-sample AUC performance difference between the escalation and cameo models for the one-month base setting. Out-of-sample evaluation results are used as the primary evidence of better model performance in the main text of the paper, hence we report that the paper's main claim relies on out-of-sample evaluations.
- **Hegre, Nygård, and Landsverk[34]**: We report the number of rows and number of positive instances for civil war onset the dates between 2001 and 2018, i.e. all years for which out-of-sample estimates are provided. We don't report the out-of-sample performance difference because the paper does not perform comparisons between models. Out-of-sample evaluation results are used as the primary evidence of model performance in the main text of the paper, hence we report that the paper's main claim relies on out-of-sample evaluations.

S3 Model info sheets can detect and prevent leakage in ML-based science

We include a template for model info sheets in the next section (Section S4). Here, we detail how model info sheets would address each type of leakage that we found in our survey, as well as the types of leakage we found in our case study of civil war prediction.

- **L1.1 No test set.** Model info sheets require an explanation of how the train and test set is split during all steps in the modeling process (Q9-17 of model info sheets).
- **L1.2 Pre-processing on training and test set.** Details of how the train and test set are separated during the preprocessing selection step need to be included in the model info sheet (Q12-13). In our civil war prediction case study, this would address leakage due to incorrect imputation[4–6].
- **L1.3 Feature selection on training and test set.** Details of how the train and test set are separated during the feature selection step need to be included in the model info sheet (Q14-15).
- **L1.4 Duplicates in datasets.** Model info sheets require details of whether there are duplicates in the dataset, and if so, how they are handled (Q10).

Paper	CI?	Stat. sig test?	Metric(s)	Num. rows in test set	Num. positive test set instances	Main Claim OOS?	OOS performance delta
Hegre et al.[28]	No	No	AUC, Brier score	2197	321	No	0.006
Muchlinski et al.[4]	No	No	AUC, F1 score	896	19	Yes	0.04
Chiba and Gleditsch[29]	No	No	AUC, Brier score	4176	15	No	0.03
Colaresi and Mahmood[5]	No	No	AUC, Precision, Recall	1778	29	Yes	0.02
Hirose, Imai, and Lyall[30]	No	*	MAE, RMSE	14,606	—	Yes	0.16
Schutte[31]	No	No	MAE	3744	—	Yes	0.09
Hegre, Hultman, and Nygård[32]	No	No	AUC	2197	321	No	0.02
Hegre et al.[33]	No	No	AUC, Brier score, AUPR, Accuracy, F1 score, cost-based threshold	384,372	1848	Yes	0.01
Kaufman, Kraft, and Sen[7]	No	No	Accuracy	6610	918	Yes	0.03
Wang[6]	Yes	No	AUC, Precision, Recall	6363	116	Yes	0.12
Blair and Sambanis[23]	No	No	AUC, Precision, Recall	15,744	11	Yes	0.03
Hegre, Nygård, and Landsverk[34]	Yes	No	AUC, AUPR, TPR/FPR	3042	79	Yes	—

Table S6: A list of papers for which code and dataset were available, showing information about whether they report confidence intervals, conduct tests of statistical significance when comparing classifier performance, which metrics they report, the number of rows and the number of positive instances (i.e. instances of war or conflict or onset thereof) in the test set, and whether their main claim relies on out-of-sample evaluation of classifier performance. AUC = Area Under ROC, MAE = Mean Absolute Error, RMSE = Root Mean Squared Error, AUPR = Area Under Precision-Recall Curve, TPR = True Positive Rate, FPR = False Positive Rate, OOS performance delta = the performance difference for the most salient performance comparison reported in the paper (details in Section S2.7). *Hirose et al. state that the out-of-sample performance is significantly better in the Supplement of their paper, but we could not find the figure they cite as evidence of this claim in their Supplement.

- **L2 Model uses features that are not legitimate.** For each feature used in the model, researchers need to argue why the feature is legitimate to be used for the modeling task at hand (Q21). This addresses the leakage due to the use of proxy variables in Kaufman, Kraft, and Sen[7].
- **L3.1 Temporal leakage.** In case the claim is about predicting future outcomes of interest based on ML methods, researchers need to provide an explanation for why the time windows used in the training and test set are separate, and why data in the test set is always a later timestamp compared to the data in the training set (Q20). This addresses the temporal leakage in Wang, Kaufman, Kraft, and Sen[6,7].
- **L3.2 Dependencies in training and test data.** Researchers need to reason about the dependencies that may exist in their dataset and outline how dependencies across training and test sets are addressed (Q11).
- **L3.3 Sampling bias in test distribution.** Researchers need to reason about the presence of selection bias in their dataset and outline how the rows included for data analysis were selected, and how the test set matches the distribution about which the scientific claims are made (Q18-19).

S4 Model Info Sheets Template

About model info sheets

Completing this model info sheet requires the researcher to provide precise arguments to justify that predictive models used for making scientific claims do not suffer from leakage. It is inspired by the model cards introduced by Mitchell et al.[1]

Model info sheets are intended to accompany the paper or report that introduces the model: for instance, as an appendix or supplemental material. For feedback or questions, contact: sayashk@princeton.edu

The model info sheet starts on the next page. After filling it out, save it starting from that page. To cite the paper that introduces the model info sheets, use the bibliography file available at reproducible.cs.princeton.edu/citation.bib

[1] Margaret Mitchell, Simone Wu, Andrew Zaldivar, Parker Barnes, Lucy Vasserman, Ben Hutchinson, Elena Spitzer, Inioluwa Deborah Raji, and Timnit Gebru. "Model cards for model reporting." In *Proceedings of the conference on fairness, accountability, and transparency*, 2019.

Model Info Sheet

Section 1: Information about paper or report

1) Author(s): Names of the authors of the paper or report

2) Title of the paper or report which introduces the model

3) DOI or permanent link to the paper or report (for example, link to arxiv.org webpage)

4) License: Under which license(s) are the data and/or model shared?

5) Email address of the corresponding author

Section 2: Scientific claim(s) of interest

6) Does your paper make a generalizable claim based on the ML model? If yes, what is the scientific claim? For example, “Our ML model can be used to diagnose Covid-19 using chest radiographs of adult patients”.

If there are multiple claims, list each claim in a new line, along with a claim number.

7) Is the scientific claim made about a distribution or population from which you can sample? If yes: (a) what is the population or distribution about which the scientific claim is being made? (b) What is the sample used for the study? For example, “(a) Population: adult patients with symptoms of Covid-19. (b) Sample: We use a random sample of adult patients who present at a U.S. based hospital between April 2020 and June 2020”.

If there are multiple scientific claims, list your answer for each claim in a new line, corresponding to their claim number in Q6.

Note: *A difference between the population and the set from which the sample is drawn could highlight potential generalizability failures, which are related to but distinct from leakage.*

8) Does the scientific claim only apply to certain subsets of the distribution mentioned in Q6? For example, "Our model works on chest radiographs of U.S.-based adult patients and might not generalize to radiographs taken in other places or using different machines."

If there are multiple claims, list your answer for each claim in a new line, corresponding to their claim number in Q6.

Section 3: Train-test split is maintained across all steps in creating the model

9) Train-test split type: How was the dataset split into train and test sets? (For example, cross-validation; separate train and test sets).

If your model does not have a separate test set, it could suffer from leakage due to overfitting

10) Are there duplicates in the dataset? If yes, explain how duplicates are handled to ensure the train-test split.

If duplicates from the training set are included in the test set, your model could suffer from leakage. The higher the percentage of duplicates in the test set, the more severe the leakage.

11) In case the dataset has dependencies (e.g., multiple rows of data from the same patient), describe how the dependencies were addressed (for example, using block-cross validation).

If dependencies across the train-test split are not addressed, your model could suffer from leakage. The higher the number of rows in the test set with dependencies, the more severe the leakage.

12) List all the pre-processing steps used in creating your model. For example, imputing missing data, normalizing feature values, selecting a subset of rows from the dataset for building the model.

13) How was the train-test split observed during each pre-processing step? If applicable, use a separate line for each step mentioned in Q12.

If the train-test split is not maintained during all pre-processing steps, your model could suffer from leakage.

14) List all the modeling steps used in creating your model. For example, feature selection, parameter tuning, model selection.

15) How was the train-test split observed during each modeling step? If applicable, use a separate line for each step mentioned in Q14.

If the train-test split is not maintained during all modeling steps, your model could suffer from leakage.

16) List all the evaluation steps used in evaluating model performance. For example, cross-validation, out-of-sample testing.

17) How was the train-test split observed during each evaluation step? If applicable, use a separate line for each step mentioned in Q16.

If the train-test split is not maintained during all evaluation steps, your model could suffer from leakage.

Section 4: Test set is drawn from the distribution of scientific interest.

18) Why is your test set representative of the population or distribution about which you are making your scientific claims?

If the test set distribution is different from the scientific claim of interest (listed in Q7), your model could suffer from leakage.

19) Explain the process for selecting the test set and why this does not introduce selection bias in the learning process.

Selection bias (for example, only choosing data from a given geographic location but expecting your model's performance to generalize to all locations) can lead to leakage.

20) In case your model is used to predict a future outcome of interest using past data, detail how data in the training set is always from a date earlier than the data in the test set.

In predictions about future outcomes of interest, using data from the future to predict in the training set the past in the test set is a form of leakage. Data in the training set should always have timestamps of an earlier time than those in the test set to avoid leakage.

Section 5: Each feature used in the model is legitimate for the task

21) List the features used in the model, alongside an argument for their legitimacy. A legitimate feature is one that would be available when the model is used in the real world and is not a proxy of the outcome being predicted. You can also include this list in an appendix and reference the relevant section of your Appendix here.

For example, “Patient age: We include this feature in our ML model for hypertension diagnosis since patient age is easily available in a clinical setting”.

An example of a feature that should not be included (for illustration only; you do not need to include these in your model info sheet): “Anti-hypertensive drugs: We do not include the use of anti-hypertensive drugs as a feature in our ML model for hypertension diagnosis since that information is only available after diagnosis and would not be available when a new patient presents with symptoms of hypertension.”

Note: *You do not need to list each feature used in your model here. However, you must provide an argument for the legitimacy of each feature included in your model to ensure that your model does not suffer from leakage due to illegitimate features. For example, “our model only uses data from the previous year as features. For instance, to predict civil war in 2017, we only use lagged features from the year 2016. Since these features are always available in advance of when we want to make predictions using our model, none of these features can lead to leakage.”*

References

[1] Engineering National Academies of Sciences. *Reproducibility and Replicability in Science*. en. May 2019. ISBN: 978-0-309-48616-3. DOI: 10.17226/25303. URL: https://www.nap.edu/catalog/25303/reproducibility-and-replicability-in-science (visited on 06/08/2021).

[2] David M. Liu and Matthew J. Salganik. "Successes and Struggles with Computational Reproducibility: Lessons from the Fragile Families Challenge". In: *Socius* 5 (2019), p. 2378023119849803. DOI: 10.1177/2378023119849803. eprint: https://doi.org/10.1177/2378023119849803. URL: https://doi.org/10.1177/2378023119849803.

[3] Max Kuhn and Kjell Johnson. *Applied Predictive Modeling*. en. New York: Springer-Verlag, 2013. ISBN: 978-1-4614-6848-6. DOI: 10.1007/978-1-4614-6849-3. URL: https://www.springer.com/gp/book/9781461468486 (visited on 05/17/2021).

[4] David Muchlinski et al. "Comparing Random Forest with Logistic Regression for Predicting Class-Imbalanced Civil War Onset Data". en. In: *Political Analysis* 24.1 (2016). Publisher: Cambridge University Press, pp. 87–103. ISSN: 1047-1987, 1476-4989. DOI: 10.1093/pan/mpv024. URL: https://www.cambridge.org/core/journals/political-analysis/article/abs/comparing-random-forest-with-logistic-regression-for-predicting-classimbalanced-civil-war-onset-data/109E1511378A38BB4B41F721E6017FB1 (visited on 05/16/2021).

[5] Michael Colaresi and Zuhaib Mahmood. "Do the robot: Lessons from machine learning to improve conflict forecasting". en. In: *Journal of Peace Research* 54.2 (Mar. 2017). Publisher: SAGE Publications Ltd, pp. 193–214. ISSN: 0022-3433. DOI: 10.1177/0022343316682065. URL: https://doi.org/10.1177/0022343316682065 (visited on 05/16/2021).

[6] Yu Wang. "Comparing Random Forest with Logistic Regression for Predicting Class-Imbalanced Civil War Onset Data: A Comment". en. In: *Political Analysis* 27.1 (Jan. 2019). Publisher: Cambridge University Press, pp. 107–110. ISSN: 1047-1987, 1476-4989. DOI: 10.1017/pan.2018.40. URL: https://www.cambridge.org/core/journals/political-analysis/article/comparing-random-forest-with-logistic-regression-for-predicting-classimbalanced-civil-war-onset-data-a-comment/B62CC1DA390C58435004D4C5D56DBF71 (visited on 05/16/2021).

[7] Aaron Russell Kaufman, Peter Kraft, and Maya Sen. "Improving Supreme Court Forecasting Using Boosted Decision Trees". en. In: *Political Analysis* 27.3 (July 2019). Publisher: Cambridge University Press, pp. 381–387. ISSN: 1047-1987, 1476-4989. DOI: 10.1017/pan.2018.59. URL: https://www.cambridge.org/core/journals/political-analysis/article/improving-supreme-court-forecasting-using-boosted-decision-trees/166AA006B8DA7C87F1B17291B0BB8B63 (visited on 05/16/2021).

[8] Daniel W. Hook, Simon J. Porter, and Christian Herzog. "Dimensions: Building Context for Search and Evaluation". English. In: *Frontiers in Research Metrics and Analytics* 3 (2018). Publisher: Frontiers. ISSN: 2504-0537. DOI: 10.3389/frma.2018.00023. URL: https://www.frontiersin.org/articles/10.3389/frma.2018.00023/full (visited on 05/13/2021).

[9] Corinne Bara. *Forecasting civil war and political violence*. en. Pages: 177-193 Publication Title: The Politics and Science of Prevision. Routledge, May 2020. ISBN: 978-1-00-302242-8. DOI: 10.4324/9781003022428-14. URL: https://www.taylorfrancis.com/https://www.taylorfrancis.com/chapters/oa-edit/10.4324/9781003022428-14/forecasting-civil-war-political-violence-corinne-bara (visited on 05/13/2021).

[10] A. Rogier T. Donders et al. "Review: A gentle introduction to imputation of missing values". en. In: *Journal of Clinical Epidemiology* 59.10 (Oct. 2006), pp. 1087–1091. ISSN: 0895-4356. DOI: 10.1016/j.jclinepi.2006.01.014. URL: https://www.sciencedirect.com/science/article/pii/S0895435606001971 (visited on 05/16/2021).

[11] Rayid Ghani, Joe Walsh, and Joan Wang. *Top 10 ways your Machine Learning models may have leakage (URL: http://www.rayidghani.com/2020/01/24/top-10-ways-your-machine-learning-models-may-have-leakage/)*. en-US. Jan. 2020. URL: http://www.rayidghani.com/2020/01/24/top-10-ways-your-machine-learning-models-may-have-leakage/ (visited on 05/16/2021).

[12] *Imputation before or after splitting into train and test?* URL: https://stats.stackexchange.com/questions/95083/imputation-before-or-after-splitting-into-train-and-test (visited on 05/16/2021).

[13] Tom Fawcett. "An introduction to ROC analysis". en. In: *Pattern Recognition Letters*. ROC Analysis in Pattern Recognition 27.8 (June 2006), pp. 861–874. ISSN: 0167-8655. DOI: 10.1016/j.patrec.2005.10.010. URL: https://www.sciencedirect.com/science/article/pii/S016786550500303X (visited on 05/16/2021).

[14] Marcel Neunhoeffer and Sebastian Sternberg. "How Cross-Validation Can Go Wrong and What to Do About It". en. In: *Political Analysis* 27.1 (Jan. 2019). Publisher: Cambridge University Press, pp. 101–106. ISSN: 1047-1987, 1476-4989. DOI: 10.1017/pan.2018.39. URL: https://www.cambridge.org/core/journals/political-analysis/article/how-crossvalidation-can-go-wrong-and-what-to-do-about-it/CA8C4B470E27C99892AB978CE0A3AE29 (visited on 05/31/2021).

[15] Jake M. Hofman et al. "Expanding the scope of reproducibility research through data analysis replications". en. In: *Organizational Behavior and Human Decision Processes* 164 (May 2021), pp. 192–202. ISSN: 0749-5978. DOI: 10.1016/j.obhdp.2020.11.003. URL: https://www.sciencedirect.com/science/article/pii/S0749597820304076 (visited on 07/13/2022).

[16] David Alan Muchlinski et al. "Seeing the Forest through the Trees". en. In: *Political Analysis* 27.1 (Jan. 2019). Publisher: Cambridge University Press, pp. 111–113. ISSN: 1047-1987, 1476-4989. DOI: 10.1017/pan.2018.45. URL: https://www.cambridge.org/core/journals/political-analysis/article/seeing-the-forest-through-the-trees/E717D15F10CC4F979EDC35C0CB9B55C1 (visited on 05/31/2021).

[17] Håvard Hegre and Nicholas Sambanis. "Sensitivity Analysis of Empirical Results on Civil War Onset:" en. In: *Journal of Conflict Resolution* (2006). Publisher: Sage PublicationsSage CA: Thousand Oaks, CA. DOI: 10.1177/0022002706289303. URL: https://journals.sagepub.com/doi/suppl/10.1177/0022002706289303 (visited on 06/02/2021).

[18] Andreas Beger. *@andybeega (Andreas Beger): This is great. One thing I'd add is that for the @DMuchlinski et al data...* http://archive.today/VV9nC. 2021. URL: http://archive.today/VV9nC (visited on 08/05/2021).

[19] Joseph L Schafer. "Multiple imputation: a primer". en. In: *Statistical Methods in Medical Research* 8.1 (Feb. 1999). Publisher: SAGE Publications Ltd STM, pp. 3–15. ISSN: 0962-2802. DOI: 10.1177/096228029900800102. URL: https://doi.org/10.1177/096228029900800102 (visited on 07/18/2021).

[20] James D. Fearon and David D. Laitin. "Ethnicity, Insurgency, and Civil War". In: *The American Political Science Review* 97.1 (2003). Publisher: [American Political Science Association, Cambridge University Press], pp. 75–90. ISSN: 0003-0554. URL: https://www.jstor.org/stable/3118222 (visited on 05/16/2021).

[21] Thomas G Dietterich. "Approximate statistical tests for comparing supervised classification learning algorithms". In: *Neural computation* 10.7 (1998), pp. 1895–1923.

[22] Paul Collier and Anke Hoeffler. "On the Incidence of Civil War in Africa". en. In: *Journal of Conflict Resolution* 46.1 (Feb. 2002). Publisher: SAGE Publications Inc, pp. 13–28. ISSN: 0022-0027. DOI: 10.1177/0022002702046001002. URL: https://doi.org/10.1177/0022002702046001002 (visited on 06/21/2021).

[23] Robert A. Blair and Nicholas Sambanis. "Forecasting Civil Wars: Theory and Structure in an Age of "Big Data" and Machine Learning". en. In: *Journal of Conflict Resolution* 64.10 (Nov. 2020). Publisher: SAGE Publications Inc, pp. 1885–1915. ISSN: 0022-0027. DOI: 10.1177/0022002720918923. URL: https://doi.org/10.1177/0022002720918923 (visited on 05/16/2021).

[24] Xavier Robin et al. "pROC: an open-source package for R and S+ to analyze and compare ROC curves". In: *BMC Bioinformatics* 12.1 (Mar. 2011), p. 77. ISSN: 1471-2105. DOI: 10.1186/1471-2105-12-77. URL: https://doi.org/10.1186/1471-2105-12-77 (visited on 07/24/2021).

[25] Guido W. Imbens. "Statistical Significance, p -Values, and the Reporting of Uncertainty". en. In: *Journal of Economic Perspectives* 35.3 (Aug. 2021), pp. 157–174. ISSN: 0895-3309. DOI: 10.1257/jep.35.3.157. URL: https://pubs.aeaweb.org/doi/10.1257/jep.35.3.157 (visited on 08/05/2021).

[26] Andreas Beger, Richard K. Morgan, and Michael D. Ward. "Reassessing the Role of Theory and Machine Learning in Forecasting Civil Conflict". en. In: *Journal of Conflict Resolution* (July 2021). Publisher: SAGE Publications Inc, p. 0022002720982358. ISSN: 0022-0027. DOI: 10.1177/0022002720982358. URL: https://doi.org/10.1177/0022002720982358 (visited on 07/26/2021).

[27] Robert A. Blair and Nicholas Sambanis. "Is Theory Useful for Conflict Prediction? A Response to Beger, Morgan, and Ward". en. In: *Journal of Conflict Resolution* (July 2021). Publisher: SAGE Publications Inc, p. 00220027211026748. ISSN: 0022-0027. DOI: 10.1177/00220027211026748. URL: https://doi.org/10.1177/00220027211026748 (visited on 07/26/2021).

[28] H\aavard Hegre et al. "Forecasting civil conflict along the shared socioeconomic pathways". en. In: *Environmental Research Letters* 11.5 (Apr. 2016). Publisher: IOP Publishing, p. 054002. ISSN: 1748-9326. DOI: 10.1088/1748-9326/11/5/054002. URL: https://doi.org/10.1088/1748-9326/11/5/054002 (visited on 06/26/2021).

[29] Daina Chiba and Kristian Skrede Gleditsch. "The shape of things to come? Expanding the inequality and grievance model for civil war forecasts with event data". en. In: *Journal of Peace Research* 54.2 (Mar. 2017). Publisher: SAGE Publications Ltd, pp. 275–297. ISSN: 0022-3433. DOI: 10.1177/0022343316684192. URL: https://doi.org/10.1177/0022343316684192 (visited on 06/26/2021).

[30] Kentaro Hirose, Kosuke Imai, and Jason Lyall. "Can civilian attitudes predict insurgent violence? Ideology and insurgent tactical choice in civil war". en. In: *Journal of Peace Research* 54.1 (Jan. 2017). Publisher: SAGE Publications Ltd, pp. 47–63. ISSN: 0022-3433. DOI: 10.1177/0022343316675909. URL: https://doi.org/10.1177/0022343316675909 (visited on 06/26/2021).

[31] Sebastian Schutte. "Regions at Risk: Predicting Conflict Zones in African Insurgencies*". en. In: *Political Science Research and Methods* 5.3 (July 2017). Publisher: Cambridge University Press, pp. 447–465. ISSN: 2049-8470, 2049-8489. DOI: 10.1017/psrm.2015.84. URL: https://www.cambridge.org/core/journals/political-science-research-and-methods/article/abs/regions-at-risk-predicting-conflict-zones-in-african-insurgencies/4DCDBA2BCC8B4E3D5057A2C37DDB2BD6 (visited on 06/26/2021).

[32] Håvard Hegre, Lisa Hultman, and Håvard Mokleiv Nygård. "Evaluating the Conflict-Reducing Effect of UN Peacekeeping Operations". In: *The Journal of Politics* 81.1 (Jan. 2019). Publisher: The University of Chicago Press, pp. 215–232. ISSN: 0022-3816. DOI: 10.1086/700203. URL: https://www.journals.uchicago.edu/doi/10.1086/700203 (visited on 06/26/2021).

[33] Håvard Hegre et al. "ViEWS: A political violence early-warning system:" en. In: *Journal of Peace Research* (Feb. 2019). Publisher: SAGE PublicationsSage UK: London, England. DOI: 10.1177/0022343319823860. URL: https://journals.sagepub.com/doi/suppl/10.1177/0022343319823860 (visited on 01/08/2021).

[34] Håvard Hegre, Håvard Mokleiv Nygård, and Peder Landsverk. "Can We Predict Armed Conflict? How the First 9 Years of Published Forecasts Stand Up to Reality". In: *International Studies Quarterly* sqaa094 (Jan. 2021). ISSN: 0020-8833. DOI: 10.1093/isq/sqaa094. URL: https://doi.org/10.1093/isq/sqaa094 (visited on 06/26/2021).
