# Supplementary material for: Leakage and the reproducibility crisis in machine-learning-based science
Source: Patterns (N Y). 2023 Aug 4;4(9):100804. doi: 10.1016/j.patter.2023.100804 (PMC10499856; doi:10.1016/j.patter.2023.100804)
Supplement: Document S2. Article plus supplemental information [file mmc2.pdf]

Article

Patterns

Leakage and the reproducibility crisis in machine-learning-based science

Highlights

- Data leakage is a flaw in machine learning that leads to overoptimistic results
- Our survey of prior reviews shows leakage affects 294 papers across 17 scientific fields
- We provide a taxonomy of leakage and introduce model info sheets to mitigate it
- We show how leakage can lead to overoptimism with a case study on civil war prediction

Authors

Sayash Kapoor, Arvind Narayanan

Correspondence

sayashk@princeton.edu

In brief

Kapoor and Narayanan show that leakage is a widespread failure mode in machine-learning (ML)-based science. Based on a survey of past reviews, they find that it affects at least 294 papers across 17 disciplines. They provide a taxonomy of eight types of leakage and propose model info sheets to mitigate it. They show that leakage can lead to severe overoptimism through a case study of civil war prediction. Several papers claimed that ML models drastically outperform older regression models. This is no longer the case when leakage is fixed.

Kapoor & Narayanan, 2023, Patterns *4*, 100804
September 8, 2023 © 2023 The Author(s).
https://doi.org/10.1016/j.patter.2023.100804

CellPress

Patterns

CellPress
OPEN ACCESS

Article

Leakage and the reproducibility crisis in machine-learning-based science

Sayash Kapoor[1,2,*] and Arvind Narayanan[1]
[1]Department of Computer Science and Center for Information Technology Policy, Princeton University, Princeton, NJ 08540, USA
[2]Lead contact
*Correspondence: sayashk@princeton.edu
https://doi.org/10.1016/j.patter.2023.100804

THE BIGGER PICTURE Machine learning (ML) is widely used across dozens of scientific fields. However, a common issue called "data leakage" can lead to errors in data analysis. We surveyed a variety of research that uses ML and found that data leakage affects at least 294 studies across 17 fields, leading to overoptimistic findings. We classified these errors into eight different types. We propose a solution: model info sheets that can be used to identify and prevent each of these eight types of leakage. We also tested the reproducibility of ML in a specific field: predicting civil wars, where complex ML models were thought to outperform traditional statistical models. Interestingly, when we corrected for data leakage, the supposed superiority of ML models disappeared: they did not perform any better than older methods. Our work serves as a cautionary note against taking results in ML-based science at face value.

1 2 **3** 4 5 **Development/Pre-production:** Data science output has been rolled out/validated across multiple domains/problems

SUMMARY

Machine-learning (ML) methods have gained prominence in the quantitative sciences. However, there are many known methodological pitfalls, including data leakage, in ML-based science. We systematically investigate reproducibility issues in ML-based science. Through a survey of literature in fields that have adopted ML methods, we find 17 fields where leakage has been found, collectively affecting 294 papers and, in some cases, leading to wildly overoptimistic conclusions. Based on our survey, we introduce a detailed taxonomy of eight types of leakage, ranging from textbook errors to open research problems. We propose that researchers test for each type of leakage by filling out model info sheets, which we introduce. Finally, we conduct a reproducibility study of civil war prediction, where complex ML models are believed to vastly outperform traditional statistical models such as logistic regression (LR). When the errors are corrected, complex ML models do not perform substantively better than decades-old LR models.

INTRODUCTION

There has been a marked shift toward the paradigm of predictive modeling across quantitative science fields. This shift has been facilitated by the widespread use of machine learning (ML) methods. However, pitfalls in using ML methods have led to exaggerated claims about their performance. Such errors can lead to a feedback loop of overoptimism about the paradigm of prediction, especially because non-replicable publications tend to be cited more often than replicable ones.[1] It is therefore important to examine the reproducibility of findings in communities adopting ML methods.

Scope. We focus on reproducibility issues in ML-based science, which involves *making a scientific claim using the performance of the ML model as evidence*. There is a better-known reproducibility crisis in research that uses traditional statistical methods.[2] We also situate our work in contrast with other ML domains, such as methods research (creating and improving widely applicable ML methods), ethics research (studying the ethical implications of ML methods), engineering applications (building or improving a product or service), and modeling contests (improving predictive performance on a fixed dataset created by an independent third party). Investigating the validity of claims in all these areas is

This is an open access article under the CC BY license (http://creativecommons.org/licenses/by/4.0/).

Field	Paper	Number of papers reviewed	Number of papers with pitfalls	**[L1.1] No test set**	**[L1.2] Pre-proc. on train-test**	**[L1.3] Feature sel. on train-test**	**[L1.4] Duplicates**	**[L2] Illegitimate features**	**[L3.1] Temporal leakage**	**[L3.2] Non-ind. b/w train-test**	**[L3.3] Sampling bias**	Comput. reproducibility issues	Data quality issues	Metric choice issues	Standard dataset used?
Medicine	Bouwmeester et al. (2012)	71	27	○									○		
Neuroimaging	Whelan & Garavan (2014)	–	14	○		○									
Bioinformatics	Blagus & Lusa (2015)	–	6		○										
Autism Diagnostics	Bone et al. (2015)	–	3				○				○		○	○	○
Nutrition Research	Ivanescu et al. (2016)	–	4	○									○	○	
Software Eng.	Tu et al. (2018)	58	11						○			○	○		○
Toxicology	Alves et al. (2019)	–	1				○					○	○		
Clinical Epidem.	Christodoulou et al. (2019)	71	48			○							○		
Satellite Imaging	Nalepa et al. (2019)	17	17							○			○		○
Tractography	Poulin et al. (2019)	4	2	○								○	○	○	○
Brain-computer Int.	Nakanishi et al. (2020)	–	1	○											○
Histopathology	Oner et al. (2020)	–	1							○					
Neuropsychiatry	Poldrack et al. (2020)	100	53	○	○								○	○	
Neuroimaging	Ahmed et al. (2021)	–	1							○					
Neuroimaging	Li et al. (2021)	122	18							○					
IT Operations	Lyu et al. (2021)	9	3						○						○
Medicine	Filho et al. (2021)	–	1					○							
Radiology	Roberts et al. (2021)	62	16	○			○				○	○			○
Neuropsychiatry	Shim et al. (2021)	–	1			○						○			
Medicine	Vandewiele et al. (2021)	24	21			○				○	○	○	○		○
Computer Security	Arp et al. (2022)	30	22	○	○	○		○	○	○	○		○	○	
Genomics	Barnett et al. (2022)	41	23			○							○		

Figure 1. Survey of 22 papers that identify pitfalls in the adoption of ML methods across 17 fields, collectively affecting 294 papers
In each field, papers adopting ML methods suffer from data leakage. The column headings for types of data leakage, shown in bold, are based on our taxonomy of data leakage. We also highlight other issues that are reported in the papers: (1) computational reproducibility (the lack of availability of code, data, and computing environment to reproduce the exact results reported in the paper); (2) data quality (e.g., small size or large amounts of missing data); (3) metric choice (using incorrect metrics for the task at hand, e.g., using accuracy for measuring model performance in the presence of heavy class imbalance); and (4) standard dataset use, where issues are found despite the use of standard datasets in a field.[16–37]

important, and there is ongoing work to address reproducibility issues in these domains.[3–6]

We define a research finding as reproducible if the code and data used to obtain the finding are available and the data are correctly analyzed.[4,7,8] This is a broader definition than computational reproducibility, when the results in a paper can be replicated using the exact code and dataset provided by the authors (see supplemental experimental procedures, section S1).

Leakage. Data leakage is a spurious relationship between the independent variables and the target variable that arises as an artifact of the data collection, sampling, or pre-processing strategy. Because the spurious relationship will not be present in the distribution about which scientific claims are made, leakage usually leads to inflated estimates of model performance.

Data leakage has long been recognized as a leading cause of errors in ML applications.[9] In formative work on leakage, Kaufman et al.[10] provide an overview of different types of error and give several recommendations for mitigating these errors. Since this paper was published, the ML community has investigated leakage in several engineering applications and modeling competitions.[11–15] However, leakage occurring in ML-based science has not been comprehensively investigated. As a result, mitigations for data leakage in scientific applications of ML remain understudied.

In this paper, we systematically investigate reproducibility issues in ML-based science as a result of data leakage. Our main contributions are as follows:

1. **A survey and taxonomy of reproducibility issues caused by leakage.** We provide evidence for a growing reproducibility crisis in ML-based science. Through a survey of literature in research communities that adopted ML methods, we find 22 papers across 17 fields where leakage has been found, collectively affecting 294 papers (Figure 1). We highlight that data leakage mitigation strategies developed for other ML applications, such as modeling contests and engineering applications, often do not translate to ML-based science. Based on our survey, we present a fine-grained taxonomy of eight types of leakage that range from textbook errors to open research problems.

2. **Model info sheets to detect and prevent leakage.** Current standards for reporting model performance in ML-based science often fall short in addressing issues caused by leakage. Specifically, checklists and model cards are one way to provide

standard best practices for reporting details about ML models.[38–40] However, current efforts do not address issues arising because of leakage. Further, most checklists currently in use are not developed for ML-based science in general but rather for specific scientific or research communities.[4,38] As a result, best practices for model reporting in ML-based science are underspecified.

We introduce model info sheets to detect and prevent leakage in ML-based science. They are inspired by the model cards in Mitchell et al.[40] Filling out a model info sheet requires the researcher to provide precise arguments to justify that models used for making scientific claims do not suffer from leakage, by answering 21 questions based on our taxonomy of leakage.

3. **An empirical case study of leakage in civil war prediction.** For an in-depth look at the impact of reproducibility errors, we undertake a reproducibility study in civil war prediction, a subfield of political science where ML models are believed to vastly outperform older statistical models such as logistic regression (LR). We perform a systematic review to find papers on civil war prediction and find that all papers in our review claiming the superior performance of complex ML models compared with KR models fail to reproduce because of data leakage.

Each of these papers was published in top political science journals. Leakage affects complex ML models, as well as simpler LR models. But when the errors caused by leakage are corrected, ML models no longer perform substantively better than decades-old LR models.

RESULTS

Evidence of a reproducibility crisis

Many scientific fields have adopted ML methods and the paradigm of predictive modeling.[41–46] We find at least three main uses of ML models in scientific literature. First, models that are better at prediction are thought to enable an improved understanding of scientific phenomena.[47] Second, especially when used in medical fields, models with higher predictive accuracy can aid in research and development of better diagnostic tools.[48] Finally, ML-based methods have also been used to investigate the inherent predictability of phenomena, especially for predicting social outcomes.[49] The increased adoption of ML methods in science motivates our investigation of reproducibility issues in ML-based science.

Data leakage causes irreproducible results

Researchers in many communities have already documented reproducibility failures in ML-based science within their fields. Here we conduct a cross-disciplinary analysis by building on these individual reviews. This enables us to highlight the scale and scope of the crisis, identify common patterns, and make progress toward a solution.

When searching for past literature that documents reproducibility failures in ML-based science, we found that different fields often use different terms to describe pitfalls and errors. This makes it difficult to conduct a systematic search to find papers with errors. Therefore, we do not present our results as a systematic meta-review of leakage from a coherent sample of papers but rather as a lower bound of reproducibility issues in ML-based science. In addition, most reviews look only at the *content* of the papers and not the code and data provided with the papers to check for errors. This leads to under-counting the number of affected papers, because the code might have errors that are not apparent from reading the papers.

We find 22 papers from 17 fields that outline errors in ML-based science in their field, collectively affecting 294 papers. A prominent finding that emerges is that data leakage is a pitfall in every single case. Our findings present a worrying trend for the reproducibility of ML-based science.

Note that leakage is one of many causes of irreproducible results. Other factors, such as the lack of available code and data, can also lead to irreproducibility, and there are several studies investigating these shortcomings.[50,51] We discuss our choice of terminology in detail in the supplemental experimental procedures (section S1).

The results from our survey are presented in Figure 1. Columns in bold represent different types of leakage. The last four columns represent other common trends in the papers we study. For systematic reviews, we report the number of papers reviewed. Each paper in our survey highlights issues with leakage, with six papers highlighting the presence of multiple types of leakage in their field.

Data leakage mitigations for other ML applications do not apply to scientific research

Most previous research and writing on data leakage has focused on mitigating data leakage in engineering settings or predictive modeling competitions.[10–12] However, the taxonomy of data leakage outlined in this body of work does not address all types of leakage that we identify in our survey. In particular, we find that leakage can result from a difference between the distribution of the test set and the distribution of scientific interest. Robustness to distribution shift is an area of ongoing research in ML methods and is as such an open problem.[52] In addition, these settings are very different from scientific research, and mitigations for data leakage in modeling competitions, as well as engineering applications of ML, often do not translate into strategies for mitigating data leakage in ML-based science.

Leakage in modeling competitions. In predictive modeling competitions, dataset creation and model evaluation are left to impartial third parties who have the expertise and incentives to avoid errors. Within this framework, none of the participants have access to the held-out evaluation set before the competition ends. In contrast, in most ML-based science, the researcher has access to the entire dataset while creating the ML models. Leakage often occurs because of the researcher having access to the entire dataset during the modeling process.

Leakage in engineering applications. Leakage in real-world applications has led to exaggerated performance estimates, even in consequential settings such as child maltreatment prediction.[53] One of the most common recommendations for detecting and mitigating leakage is to deploy the ML model at a limited scale in production. This advice is applicable only to engineering applications of ML, where the end goal is not to gain insights about a particular process but rather to serve as a component in a product. Often, a rough idea of model performance is enough to decide whether a model is good enough to be deployed in a product. Contrarily, ML-based science involves making a scientific claim using the performance of the ML model as

evidence. In addition, engineering applications of ML often operate in a rapidly changing context and have access to large datasets, so small differences in performances are often not as important, whereas scientific claims are sensitive to small performance differences between ML models.

Why do we call it a reproducibility crisis?

We say that ML-based science is suffering from a reproducibility crisis for two related reasons. First, our results show that reproducibility failures in ML-based science are systemic. In nearly every scientific field that has carried out a systematic study of reproducibility issues, papers are plagued by common pitfalls. In many systematic reviews, a majority of the papers reviewed suffer from these pitfalls. Similar problems are likely to arise in many fields that are adopting ML methods. Second, despite the urgency of addressing reproducibility failures, there are no systemic solutions that have been deployed for these failures. Scientific communities are discovering the same failure modes across disciplines but have yet to converge on best practices for avoiding reproducibility failures.

Calling attention to and addressing these widespread failures is vital to maintaining public confidence in ML-based science. At the same time, the use of ML methods is still in its infancy in many scientific fields. Addressing reproducibility failures pre-emptively in such fields can correct a lot of scientific research that would otherwise be flawed.

Toward a solution: A taxonomy of data leakage

We now provide our taxonomy of data leakage errors in ML-based science. Such a taxonomy can enable a better understanding of why leakage occurs and inform potential solutions. Our taxonomy is comprehensive and addresses data leakage arising during the data collection, pre-processing, modeling, and evaluation steps. In particular, our taxonomy addresses all cases of data leakage that we found in our survey (Figure 1). Some of the categories in our taxonomy, e.g., sampling bias [L3.3], were not considered types of leakage in prior work, but they have the same cause as other categories of leakage: spurious correlations between the outcome variables and the features. They also have the same effect: they lead to overestimates of model performance.

[L1] Lack of clean separation of training and test dataset. If the training dataset is not separated from the test dataset during all pre-processing, modeling, and evaluation steps, the model has access to information in the test set before its performance is evaluated. Because the model has access to information from the test set at training time, the model learns relationships between the predictors and the outcome that would not be available in additional data drawn from the distribution of interest. The performance of the model on these data therefore does not reflect how well the model would perform on a new test set drawn from the same distribution of data. This can happen in several ways, such as:

[L1.1] No test set. Using the same dataset for training and testing the model is a textbook example of overfitting, which leads to overoptimistic performance estimates.[54]

[L1.2] Pre-processing on training and test set. Using the entire dataset for any pre-processing steps, such as imputation or over/under sampling, results in leakage. For instance, using oversampling before splitting the data into training and test sets leads to an imperfect separation between the training and test sets because data generated using oversampling from the training set will also be present in the test set.

[L1.3] Feature selection on training and test set. Feature selection on the entire dataset results in using information about which feature performs well on the test set to make a decision about which features should be included in the model.

[L1.4] Duplicates in datasets. If a dataset with duplicates is used for the purposes of training and evaluating an ML model, the same data could exist in the training set and the test set.

[L2] Model uses features that are not legitimate. If the model has access to features that should not be legitimately available for use in the modeling exercise, this could result in leakage. One instance when this can happen is if a feature is a proxy for the outcome variable.[10] For example, Filho et al.[55] find that a recent study included the use of anti-hypertensive drugs as a feature for predicting hypertension. Such a feature could lead to leakage because the model would not have access to this information when predicting the health outcome for a new patient. Further, if the fact that a patient uses anti-hypertensive drugs is already known at prediction time, the prediction of hypertension becomes a trivial task.

The judgment of whether the use of a given feature is legitimate for a modeling task requires domain knowledge and can be highly problem specific. As a result, we do not provide subcategories for this sort of leakage. Instead, we suggest that researchers clearly specify which features are suitable for a modeling task and justify their choice using domain expertise.

[L3] Test set is not drawn from the distribution of scientific interest. The distribution of data on which the performance of an ML model is evaluated differs from the distribution of data about which the scientific claims are made. The performance of the model on the test set does not correspond to its performance on data drawn from the distribution of scientific interest.

[L3.1] Temporal leakage. When an ML model is used to make predictions about a future outcome of interest, the test set should not contain any data from a date before the training set. If the test set contains data from before the training set, the model is built using data "from the future" that it should not have access to during training and can cause leakage.

[L3.2] Nonindependence between training and test samples. Nonindependence between training and test samples constitutes leakage, unless the scientific claim is about a distribution that has the same dependence structure. In the extreme (but unfortunately common) case, training and test samples come from the same people or units. For example, Oner et al.[56] find that a recent study on histopathology uses different observations of the same patient in the training and test sets. In this case, the scientific claim is being made about the ability to predict gene mutations in new patients; however, it is evaluated on data from old patients (i.e., data from patients in the training set), leading to a mismatch between the test set distribution and the scientific claim. Similarly, for predicting protein function, the family of the protein can lead to dependencies if proteins from the same family are split across the training and test sets.[57] The train-test split should account for the dependencies in the data to ensure correct performance evaluation. Methods such as "block cross-validation" can partition the dataset strategically so that the performance evaluation does not suffer from data leakage and

overoptimism.[58,59] Handling nonindependence between the training and test sets in general (i.e., without any assumptions about independence in the data) is a hard problem, because we might not know the underlying dependency structure of the task in many cases.[60]

[L3.3] Sampling bias in test distribution. Sampling bias in the choice of test dataset can lead to data leakage. One example of sampling bias is spatial bias, which refers to choosing the test data from a geographic location but making claims about model performance in other geographic locations. Another example is selection bias, which entails choosing a non-representative subset of the dataset for evaluation. For example, Bone et al.[61] highlight that in a study on predicting autism using ML models, excluding the data corresponding to borderline cases of autism leads to leakage because the test set is no longer representative of the general population about which claims are made. In addition, borderline cases of autism are often the trickiest to diagnose, so excluding them from the evaluation set is likely to lead to overoptimistic results. Cases of leakage caused by sampling bias can often be subtle. For example, Zech et al.[62] find that models for pneumonia prediction trained on images from one hospital do not generalize to images from another hospital because of subtle differences in how images are generated in each hospital.

A model may have leakage when the distribution about which the scientific claim is made does not match the distribution from which the evaluation set is drawn. ML models may also suffer from a related but distinct limitation: the lack of generalization when we try to apply a result about one population to another similar but distinct population. Several issues with the generalization of ML models operating under a distribution shift have been highlighted in ML methods research, such as fragility toward adversarial examples,[63] image distortion and texture,[64] and overinterpretation.[65] Robustness to distribution shift is an ongoing area of work in ML methods research. Even slight shifts in the target distribution can cause performance estimates to change drastically.[66] Despite ongoing work to create ML methods that are robust to distribution shift, best practices to deal with distribution shift currently include testing the ML models on the data from the distribution we want to make claims about.[52] In ML-based science, where the aim is to create generalizable knowledge, we should take results that claim to generalize to a different population from the one models were evaluated on with caution.

Other issues identified in our survey

Computational reproducibility issues. Computational reproducibility of a finding refers to sharing the complete code and data needed to reproduce the findings reported in a paper exactly. This is important to enable external researchers to reproduce results and verify their correctness. Five papers in our survey outlined the lack of computational reproducibility in their field.

Data quality issues. Access to good-quality data is essential for creating ML models.[67,68] Issues with the quality of the dataset could affect the results of ML-based science. Ten papers in our survey highlighted data quality issues such as not addressing missing values in the data, the small size of datasets compared with the number of predictors, and the outcome variable being a poor proxy for the phenomenon being studied.

Metric choice issues. A mismatch between the metric used to evaluate performance and the scientific problem of interest leads to issues with performance claims. For example, using accuracy as the evaluation metric with a heavily imbalanced dataset leads to overoptimistic results, because the model can get a high accuracy score by always predicting the majority class. Four papers in our survey highlighted metric choice issues.

Use of standard datasets. Reproducibility issues arose despite the use of standard, widely used datasets, often because of the lack of standard modeling and evaluation procedures such as fixing the train-test split and evaluation metric for the dataset. Seven papers in our survey highlighted that issues arose despite the use of standard datasets.

Model info sheets for detecting and preventing leakage

Our taxonomy of data leakage highlights several failure modes that are prevalent in ML-based science. To detect cases of leakage, we provide a template for a model info sheet to accompany scientific claims using predictive modeling as a supplemental document (supplemental experimental procedures, section S4). The template consists of 21 questions that elicit precise arguments needed to justify the absence of leakage.

Prior work on model cards and reporting standards

Our proposal is inspired by prior work on model cards and checklists, which we now review. Mitchell et al.[40] introduced model cards for reporting details about ML models, with a focus on precisely reporting the intended use cases of ML models. They also addressed fairness and transparency concerns: they require that the performance of ML models on different groups of users (e.g., on the basis of race, gender, and age) is reported and documented transparently. These model cards complement the datasheets introduced by Gebru et al.[69] to document details about datasets in a standard format.

The use of checklists has also been impactful in improving reporting practices in the few fields that have adopted them.[70] Although checklists and model cards provide concrete best practices for reporting standards,[38–40,71] current efforts do not address pitfalls arising because of leakage. Further, even though several scientific fields, especially those related to medicine, have adopted checklists to improve reporting standards, most checklists are developed for specific scientific or research communities instead of ML-based science in general.

Scientific arguments to surface and prevent leakage

When ML models are used to make scientific claims, it is not enough to simply separate the training and test sets and report performance metrics on the test set. Unlike research in ML methods, where a model's performance on a hypothetical task (i.e., one that is not linked to a specific scientific claim) is still of interest to the researcher in some cases,[72] in ML-based science, claims about a model's performance need to be connected to scientific claims using explicit arguments. The burden of proof for ensuring the correctness of these arguments is on the researcher making the scientific claims.[73]

In our model info sheet, we ask researchers to answer 21 questions. These questions help them present three arguments that are essential for determining that scientific results that use ML methods do not suffer from data leakage. Note that most ML-based science papers do not present any of the three

arguments, although they sometimes partially address the first argument (clean train-test separation) by reporting out-of-sample prediction performance. The arguments below are based on our taxonomy of data leakage issues and inform the main sections of the model info sheet.

[L1] Clean train-test separation. The researcher needs to argue why the test set does not interact with training data during any of the pre-processing, modeling, or evaluation steps to ensure a clean train-test separation.

[L2] Each feature in the model is legitimate. The researcher needs to argue why each feature used in their model is legitimate, i.e., a claim made using each feature is of scientific interest. Note that some models might use hundreds of features. In such cases, it is even more important to reason about the correctness of the features used, because the incorrect use of a single feature in the model can cause leakage. That said, the same argument for why a feature is legitimate can often apply to a whole set of features. For example, for a study using individuals' location history as a feature vector, the use of the entire vector can be justified together. Note that we do not ask for the researcher to list each feature used in their model; rather, we ask that the justification provided for the legitimacy of the features used in their model should cover every feature used in their model.

[L3] Test set is drawn from the distribution of scientific interest. If the distribution about which the scientific claims are made is different from the one on which the model is tested, then any claims about the performance of an ML model on the evaluation step fall short. The researcher needs to justify that the test set is drawn from the distribution of scientific interest and there is no selection or sampling bias in the data collection process. This step can help clarify the distribution regarding which scientific claims are being made and detect temporal leakage.

Model info sheets and our theory of change

Model info sheets can influence research practices in two ways: first, researchers who introduce a scientific model alongside a paper can use model info sheets to detect and prevent leakage in their models. These info sheets can be included as supplementary materials with their paper for transparently reporting details about their models. In scientific fields where the use of ML methods is not yet widespread, using transparent reporting practices at an early stage could enable easier adoption and more trust in ML methods. This would also help assuage reviewer concerns about reproducibility.

Second, journal submission guidelines could encourage or require authors to fill out model info sheets if a paper does not transparently report how the model was created. In this case, model info sheets can be used to start a conversation between authors and reviewers about the details of the models introduced in a paper. Current peer-review practices often do not require the authors to disclose any code or data during the review process.[74] Even if the code and data are available to reviewers, reproducing results and spotting errors in code is a time-consuming process that often cannot be carried out under current peer-review practices. Model info sheets offer a middle ground: they could enable a closer scrutiny of methods without making the process onerous for reviewers.

Limitations of model info sheets

Although model info sheets can enable the detection of all types of leakage we identify in our survey, they suffer from limitations owing to the lack of computational reproducibility of results in scientific research, incorrect claims made in model info sheets, and the lack of expertise of authors and reviewers.

First, the claims made in model info sheets cannot be verified in the absence of computational reproducibility. That is, unless the code, data, and computing environment required to reproduce the results in a paper are made available, there is no way to ascertain whether model info sheets are filled out correctly. Ensuring the computational reproducibility of results therefore remains an important goal for improving scientific research standards.

Second, incorrect claims made in model info sheets might provide false assurances to reviewers about the correctness of the claims made in a paper. However, by requiring authors to precisely state details about their modeling process, model info sheets enable incorrect claims to be challenged more directly than in status quo, where details about the modeling process are often left undisclosed.

Filling out and evaluating model info sheets requires some expertise in ML. In fields where both authors and reviewers lack any ML expertise, subtle cases of leakage might slip under the radar despite the use of model info sheets. In such cases, we hope that model info sheets released publicly along with papers will enable discourse within scientific communities on the shortcomings of scientific models.

Finally, we acknowledge that our understanding of leakage may evolve, and model info sheets may need to evolve with it. To that end, we have versioned model info sheets, and plan to update them as we continue to better understand leakage in ML-based science.

A case study of civil war prediction

To understand the impact of data leakage, we undertake a reproducibility study in a field where ML models are believed to vastly outperform older statistical models such as LR for predictive modeling: civil war prediction.

Over the last few years, this field has switched to predictive modeling using complex ML models such as Random Forests and Adaboost instead of LR (see Figure 2), with several papers claiming near-perfect performance of these models for civil war prediction.[75–78] The goal of these papers is to predict civil war in a region and time period using features such as GDP, poverty rates, whether it is a democracy, etc. This is in contrast with the field's earlier focus on understanding and explaining past conflicts. Table S6 gives an overview of the training data used for the papers we considered. For a detailed overview of the recent turn to predictive modeling in this field, see Bara.[79]

Although the literature we reviewed in our survey highlighted the pitfalls in adopting ML methods (Figure 1), we go further than most previous research to investigate whether the claims made in the reviewed studies survive once the errors are corrected.

Systematic search of predictive modeling literature in civil war research

We conducted a systematic search to find relevant literature (detailed in supplemental experimental procedures, section

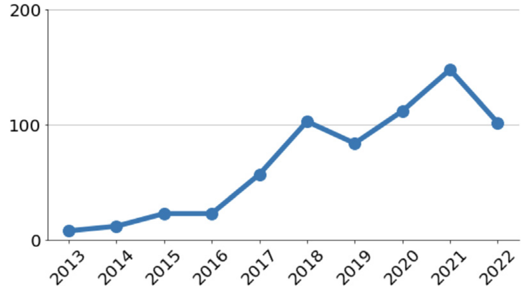

Figure 2. The sharp increase in civil war papers that use ML methods in the last few years

The number of political science papers containing the terms "civil war" and "machine learning" in the dimensions database of academic research.[104]

S2.1). This yielded 124 papers. We narrowed this list to the 12 papers that focused on predicting civil war, evaluated performance using a train-test split, and shared the complete code and data. For these 12, we attempted to identify errors and reproducibility issues from the text and by reviewing the code provided with the papers. When we identified errors, we re-analyzed the data with the errors corrected.

Finding 1: Data leakage causes irreproducible results. We present our results in Figure 3. We found errors in 4 of the 12 papers—exactly the 4 papers that claimed superior performance of complex ML models over baseline LR models for predicting civil war. Each paper suffered from different forms of leakage. All 4 papers were published in top-10 journals in the fields of political science and international relations.[80] When the errors are corrected, complex ML models perform no better than baseline LR models in each case except Wang,[77] where the difference between the area under the curve (AUC) of the complex ML models and LR models drops from 0.14 to 0.01. This is despite the fact that the LR models were not trained to optimize predictive accuracy: they were conceived as explanatory models to understand past conflicts instead of predicting future ones.[47,81,82]

We test our model info sheets on the four civil war prediction papers with errors and find that they would detect each type of leakage we identified in these papers (supplemental experimental procedures, section S3). Note that leakage affects both simple and complex models for civil war prediction. However, because of higher model capacity, complex ML models tend to over-fit to spurious correlations more easily in this case (supplemental experimental procedures, section S2.2).

Beyond reproducibility, our results show that complex ML models are not substantively better at civil war prediction than decades-old LR models. This is consistent with similar sobering findings in other tasks involving predicting social outcomes, such as children's life outcomes[49] and recidivism.[83] Although prior work has found that some fields will benefit from the use of ML methods,[84] our findings suggest the need for tempering the optimism about predictive modeling in the field of civil war prediction and question the use of ML models in this field. We provide a detailed overview of our methodology for correcting the errors and show that our results hold under several robustness checks in the supplemental experimental procedures, section S2.

Finding 2: No significance testing or uncertainty quantification. We found that 9 of the 12 papers for which complete code and data were available included no significance tests or uncertainty quantification for classifier performance comparison (Table S6). Especially when sample sizes are small, significance testing and uncertainty quantification are important steps toward reproducibility.[48,85] As an illustration, we examine this issue in detail in the case of Blair and Sambanis[86] because their test dataset has a particularly small number of instances of civil war onset (only 11). They propose a model of civil war onset that uses theoretically informed features and report that it outperforms other baseline models of civil war onset using the AUC metric on an out-of-sample dataset. We find that the performance of their model is not significantly better than other baseline models for civil war prediction (Z = 0.64, 1.09, 0.42, and 0.67; p = 0.26, 0.14, 0.34, and 0.25 for a one-tailed significance test comparing the smoothed AUC performance of the model proposed in the paper—the escalation model—with other baseline models reported in their paper—*quad*, *goldstein*, *cameo*, and *average*, respectively). We implement the comparison test for smoothed receiver operating characteristic curves detailed by Robin et al.[87] Note that we do not correct for multiple comparisons; such a correction would further reduce the significance of the results. Further, all models have large confidence intervals for their out-of-sample performance. For instance, while the smoothed AUC performance reported by the authors is 0.85, the 95% confidence interval calculated using bootstrapped test set re-sampling is 0.66–0.95.

Lack of standard reporting practices for ML-based science

Our hypothesis for why leakage is prevalent is that current standards for reporting model performance in ML-based science often fall short in addressing leakage. Specifically, checklists and model cards are one way to provide standard best practices for reporting details about ML models.[38–40] However, current efforts do not address issues arising because of leakage. Further, most checklists currently in use are not developed for ML-based science in general but rather for specific scientific or research communities.[4,38] As a result, best practices for model reporting in ML-based science are underspecified.

DISCUSSION

Beyond leakage: Perspectives on enhancing the reproducibility of ML-based science

We found a number of other reproducibility issues in our survey not limited to leakage. Here, we present five diagnoses for reproducibility failures in fields adopting ML methods. Each of our diagnoses is paired with a recommendation to address it.

[D1] Lack of understanding of the limits to prediction. Recent research for predicting social outcomes has shown that even with complex models and large datasets, there are strong limits to predictive performance.[49,83] However, results such as the better-than-human performance of ML models in perception tasks such as image classification[88,89] give the impression of ML models surpassing human performance across tasks, which can confuse researchers about the performance they should realistically expect from ML models.

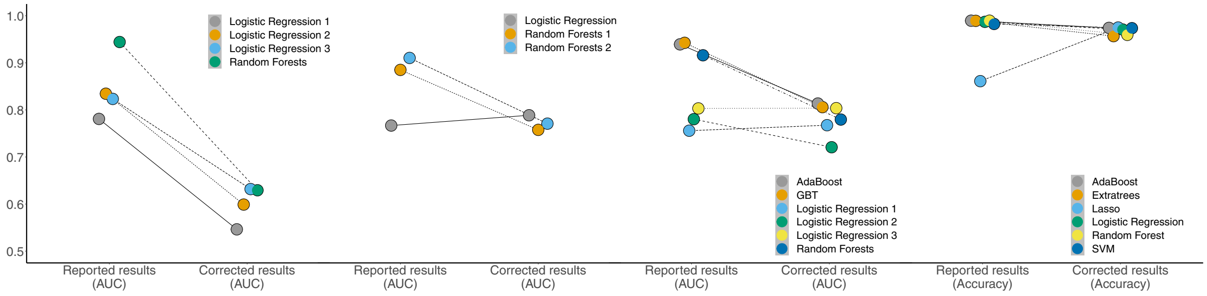

Paper	Muchlinski et al.	Colaresi and Mahmood	Wang	Kaufman et al.
**Claim**	Random Forests model drastically outperforms Logistic regression models	Random Forests models drastically outperform Logistic regression model	Adaboost and Gradient Boosted Trees (GBT) drastically outperform other models	Adaboost outperforms other models
**Error**	**[L1.2] Pre-proc. on train-test** (Incorrect imputation)	**[L1.2] Pre-proc. on train-test** (Incorrect reuse of an imputed dataset)	**[L1.2] Pre-proc. on train-test**. (Incorrect reuse of an imputed dataset) **[L3.1] Temporal leakage** (*k*-fold cross validation with temporal data)	**[L2] Illegitimate features** (Data leakage due to proxy variables) **[L3.1] Temporal leakage** (*k*-fold cross validation with temporal data)
**Impact**	Random Forests perform no better than Logistic Regression	Random Forests perform no better than Logistic Regression	Difference in AUC between Adaboost and Logistic Regression drops from 0.14 to 0.01	Adaboost no longer outperforms Logistic Regression. None of the models outperform a baseline model that predicts the outcome of the previous year
**Discussion**	Impact of the incorrect imputation is severe since 95% of the out-of-sample dataset is missing and is filled in using the incorrect imputation method	Re-use the dataset provided by Muchlinski et al., which uses an incorrect imputation method	Re-use the dataset provided by Muchlinski et al., which uses an incorrect imputation method	Use several proxy variables for the outcome as predictors (e.g., *colwars, cowwars, sdwars*, all proxies for civil war), leading to near perfect accuracy

Figure 3. A comparison of reported and corrected results in civil war prediction papers published in top political science journals
The main findings of each of these papers are invalid due to various forms of data leakage: Muchlinski et al.[75] impute the training and test data together, Colaresi and Mahmood[76] and Wang[77] incorrectly reuse an imputed dataset, and Kaufman et al.[78] use proxies for the target variable that cause data leakage. When we correct these errors, complex ML models (such as Adaboost and Random Forests) do not perform substantively better than decades-old logistic regression models for civil war prediction in each case. Each column in the table outlines the impact of leakage on the results of a paper. The figure above each column shows the difference in performance that results from fixing leakage.

[R1] Understand and communicate limits to prediction. A research agenda that investigates the efficacy of ML models in tasks across scientific fields would increase our understanding of the limits to prediction. This can alleviate the overoptimism that arises from confusing progress in one task (e.g., image classification) with another (e.g., predicting social outcomes). If we can identify upper bounds on the predictive accuracy of tasks (i.e., lower bound of the Bayes Error Rate for a task), then once the achievable accuracy has been reached, we can avoid a futile effort to increase it further and can apply increased skepticism toward results that claim to violate known bounds.

[D2] Hype, overoptimism, and publication biases. The hype about commercial AI applications can spill over into ML-based science, leading to overoptimism about their performance. Non-replicable findings are cited more than replicable ones,[1] which can result in feedback loops of overoptimism in ML-based science. Besides, publication biases that have been documented in several scientific fields[90,91] can also affect ML-based science.[92,93]

[R2] Treat results from ML-based science as tentative. When overoptimism is prevalent in a field, it is important to engage with results emerging from the field critically. Until reproducibility issues in ML-based science are widely addressed and resolved, results from this body of work should be treated with caution. Researchers, journal editors, and policymakers who use scientific research to inform real-world policy decisions should look beyond headline performance numbers when assessing papers.

[D3] Inadequate expertise. The rapid adoption of ML methods in a scientific field can lead to errors. These can be caused by the lack of expertise of domain experts in using ML methods and vice versa.

[R3] Interdisciplinary collaborations and communication of best practices. Literature in the ML community should address the different failure modes that arise during the modeling process. Researchers with expertise in ML methods should clearly communicate best practices in deploying ML for scientific research.[94] Having an interdisciplinary team consisting of researchers with domain expertise and ML expertise can avoid errors.

[D4] Lack of standardization. Several applied ML fields, such as engineering applications and modeling contests, have adopted practices such as standardized train-test splits, evaluation metrics, and modeling tasks to ensure the validity of the modeling and evaluation process.[95,96] However, these have not yet been adopted widely in ML-based science. This leads to subtle errors in the modeling process that can be hard to detect.

[R4] Adopt the common task framework when possible. The common task framework allows us to compare the performance of competing ML models using an agreed-upon training dataset and evaluation metrics, a secret holdout dataset, and a public leaderboard.[97,98] Dataset creation and model evaluation are left to impartial third parties who have the expertise and

incentives to avoid errors. However, one undesirable outcome that has been observed in communities that have adopted the common task framework is a singular focus on optimizing a particular accuracy metric to the exclusion of other scientific and normatively desirable properties of models.[67,85,99]

[D5] Lack of computational reproducibility. The lack of computational reproducibility hinders verification of results by independent researchers. Although computational reproducibility does not mean that the code is error free, it can make the process of finding errors easier, because researchers attempting to reproduce results do not have to spend time getting the code to run.

[R5] Ensure computational reproducibility. Platforms such as CodeOcean,[100] a cloud computing platform that replicates the exact computational environment used to create the original results, can be used to ensure the long-term reproducibility of results. We follow several academic journals and researchers in recommending that future research in fields using ML methods should use similar methods to ensure computational reproducibility.[74,101]

Conclusions

The attractiveness of adopting ML methods in scientific research is in part due to the widespread availability of off-the-shelf tools to create models without expertise in ML methods.[102] However, this laissez-faire approach leads to common pitfalls spreading to all scientific fields that use ML. So far, each research community has independently rediscovered these pitfalls. Without fundamental changes to research and reporting practices, we risk losing public trust because of the severity and prevalence of the reproducibility crisis across disciplines. Our paper is a call for interdisciplinary efforts to address the crisis by developing and driving the adoption of best practices for ML-based science. Model info sheets for detecting and preventing leakage are a first step in that direction.

EXPERIMENTAL PROCEDURES

Resource availability

Lead contact

Further information and requests for resources should be directed to and will be fulfilled by the lead contact, Sayash Kapoor (sayashk@princeton.edu).

Materials availability

This study did not generate new materials.

Data and code availability

The code and data required to reproduce our case study on civil war prediction have been uploaded to a CodeOcean capsule (CodeOcean: https://doi.org/10.24433/CO.4899453.v1).[103] The supplemental experimental procedures (section S2) contains a detailed description of our methods and results from additional robustness checks.

SUPPLEMENTAL INFORMATION

Supplemental information can be found online at https://doi.org/10.1016/j.patter.2023.100804.

ACKNOWLEDGMENTS

We are grateful to Jessica Hullman, Matthew J. Salganik, and Brandon Stewart for their valuable feedback on drafts of this paper. We thank Robert Blair, Aaron Kaufman, David Muchlinski, and Yu Wang for their quick and helpful responses to drafts of this paper. We are especially thankful to Matthew Sun, who reviewed our code and provided helpful suggestions and corrections for ensuring the computational reproducibility of our own results, and to Angelina Wang, Orestis Papakyriakopolous, and Anne Kohlbrenner for their feedback on model info sheets. This material is based upon work supported by the National Science Foundation under grant NSF IIS-1763642.

AUTHOR CONTRIBUTIONS

S.K. and A.N. contributed to all aspects of this paper.

DECLARATION OF INTERESTS

The authors declare no competing interests.

Received: March 3, 2023
Revised: May 18, 2023
Accepted: July 5, 2023
Published: August 4, 2023

REFERENCES

1. Serra-Garcia, M., and Gneezy, U. (2021). Nonreplicable publications are cited more than replicable ones. Sci. Adv. *7*, eabd1705. Publisher: American Association for the Advancement of Science Section: Research Article.
2. (2015). Open Science Collaboration Estimating the reproducibility of psychological science. Science *349*. Publisher: American Association for the Advancement of Science Section: Research Article. https://doi.org/10.1126/science.aac4716.
3. Hullman, J., Kapoor, S., Nanayakkara, P., Gelman, A., and Narayanan, A. (2022). The worst of both worlds: A comparative analysis of errors in learning from data in psychology and machine learning. Preprint at arXiv. https://doi.org/10.48550/arXiv.2203.06498.
4. Pineau, J.; Vincent-Lamarre, P.; Sinha, K.; Larivière, V.; Beygelzimer, A.; d'Alché-Buc, F.; Fox, E.; Larochelle, H. Improving Reproducibility in Machine Learning Research (A Report from the NeurIPS 2019 Reproducibility Program).Preprint at arXiv:https://doi.org/10.48550/arXiv.2003.122062003.12206 [cs, stat] 2020, arXiv: 2003.12206.
5. Erik Gundersen, O. (2021). The fundamental principles of reproducibility. Philosophical Transactions of the Royal Society *379*, 20200210. Publisher: Royal Society.
6. Bell, S.J., and Kampman, O.P. (2021). Perspectives on Machine Learning from Psychology's Reproducibility Crisis.
7. Hofman, J.M., Goldstein, D.G., Sen, S., Poursabzi-Sangdeh, F., Allen, J., Dong, L.L., Fried, B., Gaur, H., Hoq, A., Mbazor, E., et al. (2021). Expanding the scope of reproducibility research through data analysis replications. Organ. Behav. Hum. Decis. Process. *164*, 192–202.
8. Leek, J.T., and Peng, R.D. (2015). Opinion: Reproducible research can still be wrong: Adopting a prevention approach. Proc. Natl. Acad. Sci. USA *112*, 1645–1646. Publisher: National Academy of Sciences Section: Opinion.
9. Nisbet, R., Elder, J., and Miner, G. (2009). Handbook of Statistical Analysis and Data Mining Applications (Elsevier).
10. Kaufman, S., Rosset, S., Perlich, C., and Stitelman, O. (2012). Leakage in data mining: Formulation, detection, and avoidance. ACM Trans. Knowl. Discov. Data *6*, 1–15.
11. Fraser, C. (2016). The Treachery of Leakage. en.
12. Ghani, R., Walsh, J., and Wang, J. (2020). Top 10 Ways Your Machine Learning Models May Have Leakage. en-US. http://www.rayidghani.com/2020/01/24/top-10-ways-your-machine-learning-models-may-have-leakage/).
13. Becker, D. (2018) (Data Leakage), en.
14. Brownlee, J. (2016). Data Leakage in Machine Learning. en-US.
15. Collins-Thompson, K. Data Leakage - Module 4: Supervised Machine Learning - Part 2, en.

16. Bouwmeester, W., Zuithoff, N.P.A., Mallett, S., Geerlings, M.I., Vergouwe, Y., Steyerberg, E.W., Altman, D.G., and Moons, K.G.M. (2012). Reporting and Methods in Clinical Prediction Research: A Systematic Review. PLOS Med. *9*, e1001221. https://doi.org/10.1371/journal.pmed.1001221.

17. Whelan, R., and Garavan, H. (2014). When Optimism Hurts: Inflated Predictions in Psychiatric Neuroimaging. Biol. Psychiatry *75*, 746–748. https://doi.org/10.1016/j.biopsych.2013.05.014.

18. Blagus, R., and Lusa, L. (2015). Joint Use of Over- and under-Sampling Techniques and Cross-Validation for the Development and Assessment of Prediction Models. BMC Bioinformatics *16*, 363. https://doi.org/10.1186/s12859-015-0784-9.

19. Bone, D., Goodwin, M.S., Black, M.P., Lee, C.-C., Audhkhasi, K., and Narayanan, S. (2015). Applying Machine Learning to Facilitate Autism Diagnostics: Pitfalls and Promises. J. Autism Dev. Disord. *45*, 1121–1136. https://doi.org/10.1007/s10803-014-2268-6.

20. Ivanescu, A.E., Li, P., George, B., Brown, A.W., Keith, S.W., Raju, D., and Allison, D.B. (2016). The Importance of Prediction Model Validation and Assessment in Obesity and Nutrition Research. Int. J. Obes. *40*, 887–894. https://doi.org/10.1038/ijo.2015.214.

21. Tu, F., Zhu, J., Zheng, Q., and Zhou, M. (2018). Be Careful of When: An Empirical Study on Time-Related Misuse of Issue Tracking Data. In Proceedings of the 2018 26th ACM Joint Meeting on European Software Engineering Conference and Symposium on the Foundations of Software Engineering (ACM: Lake Buena Vista FL USA), pp. 307–318. https://doi.org/10.1145/3236024.3236054.

22. Alves, V.M., Borba, J., Capuzzi, S.J., Muratov, E., Andrade, C.H., Rusyn, I., and Tropsha, A. (2019). Oy Vey! A Comment on "Machine Learning of Toxicological Big Data Enables Read-Across Structure Activity Relationships Outperforming Animal Test Reproducibility. Toxicol. Sci. *167*, 3–4. https://doi.org/10.1093/toxsci/kfy286.

23. Christodoulou, E., Ma, J., Collins, G.S., Steyerberg, E.W., Verbakel, J.Y., and Van Calster, B. (2019). A Systematic Review Shows No Performance Benefit of Machine Learning over Logistic Regression for Clinical Prediction Models. J. Clin. Epidemiol. *110*, 12–22. https://doi.org/10.1016/j.jclinepi.2019.02.004.

24. Nalepa, J., Myller, M., and Kawulok, M. (2019). Validating Hyperspectral Image Segmentation. IEEE Geosci. Remote Sens. Lett. *16*, 1264–1268. https://doi.org/10.1109/LGRS.2019.2895697.

25. Poulin, P., Jörgens, D., Jodoin, P.-M., and Descoteaux, M. (2019). Tractography and Machine Learning: Current State and Open Challenges. Magn. Reson. Imaging *64*, 37–48. https://doi.org/10.1016/j.mri.2019.04.013.

26. Nakanishi, M., Xu, M., Wang, Y., Chiang, K.-J., Han, J., and Jung, T.-P. (2020). Questionable Classification Accuracy Reported in "Designing a Sum of Squared Correlations Framework for Enhancing SSVEP-Based BCIs. IEEE Trans. Neural Syst. Rehabil. Eng. *28*, 1042–1043. https://doi.org/10.1109/TNSRE.2020.2974272.

27. Oner, M.U., Cheng, Y.-C., Lee, H.K., and Sung, W.-K. (2020). Training Machine Learning Models on Patient Level Data Segregation Is Crucial in Practical Clinical Applications (techreport). https://doi.org/10.1101/2020.04.23.20076406.

28. Poldrack, R.A., Huckins, G., and Varoquaux, G. (2020). Establishment of Best Practices for Evidence for Prediction A Review. JAMA Psychiatry *77*, 534–540. https://doi.org/10.1001/jamapsychiatry.2019.3671.

29. Ahmed, H., Wilbur, R.B., Bharadwaj, H., and Siskind, J.M. (2021). Confounds in the Data—Comments on "Decoding Brain Representations by Multimodal Learning of Neural Activity and Visual Features". IEEE Trans. Pattern Anal. Mach. Intell. 1–1. https://doi.org/10.1109/TPAMI.2021.3121268.

30. Li, R., Johansen, J.S., Ahmed, H., Ilyevsky, T.V., Wilbur, R.B., Bharadwaj, H.M., and Siskind, J.M. (2021). The Perils and Pitfalls of Block Design for EEG Classification Experiments. IEEE Trans. Pattern Anal. Mach. Intell. *43*, 316–333. https://doi.org/10.1109/TPAMI.2020.2973153.

31. Lyu, Y., Li, H., Sayagh, M., (Jack) Jiang, Z.M., and Hassan, A.E. (2021). An Empirical Study of the Impact of Data Splitting Decisions on the Performance of AIOps Solutions. ACM Trans. Software Eng. Method. *30*, 1–38. https://doi.org/10.1145/3447876.

32. Filho, A.C., Batista, A.F.D.M., and dos Santos, H.G. (2021). Data Leakage in Health Outcomes Prediction With Machine Learning. Comment on "Prediction of Incident Hypertension Within the Next Year: Prospective Study Using Statewide Electronic Health Records and Machine Learning. J. Med. Internet Res. *23*, e10969. https://doi.org/10.2196/10969.

33. Roberts, M., Driggs, D., Thorpe, M., Gilbey, J., Yeung, M., Ursprung, S., Aviles-Rivero, A.I., Etmann, C., McCague, C., Beer, L., Weir-McCall, J.R., Teng, Z., Gkrania-Klotsas, E., Rudd, J.H.F., Sala, E., and Schönlieb, C.-B. (2021). Common Pitfalls and Recommendations for Using Machine Learning to Detect and Prognosticate for COVID-19 Using Chest Radiographs and CT Scans. Nat. Mach. Intell. *3*, 199–217. https://doi.org/10.1038/s42256-021-00307-0.

34. Shim, M., Lee, S.-H., and Hwang, H.-J. (2021). Inflated Prediction Accuracy of Neuropsychiatric Biomarkers Caused by Data Leakage in Feature Selection. Sci. Rep. *11*, 7980. https://doi.org/10.1038/s41598-021-87157-3.

35. Vandewiele, G., Dehaene, I., Kovács, G., Sterckx, L., Janssens, O., Ongenae, F., De Backere, F., De Turck, F., Roelens, K., Decruyenaere, J., Van Hoecke, S., and Demeester, T. (2021). Overly Optimistic Prediction Results on Imbalanced Data: A Case Study of Flaws and Benefits When Applying over-Sampling. Artif. Intell. Med. *111*, 101987. https://doi.org/10.1016/j.artmed.2020.101987.

36. Arp, D., Quiring, E., Pendlebury, F., Warnecke, A., Pierazzi, F., Wressnegger, C., Cavallaro, L., and Rieck, K. (2022). Dos and Don'ts of Machine Learning in Computer Security (USENIX Security Symposium).

37. Barnett, E., Onete, D., Salekin, A., and Faraone, S.V. (2022). Genomic Machine Learning Meta-Regression: Insights on Associations of Study Features with Reported Model Performance; techreport. medRxiv. 2022.01.10.22268751.

38. Mongan, J., Moy, L., and Kahn, C.E. (2020). Checklist for Artificial Intelligence in Medical Imaging (CLAIM): A Guide for Authors and Reviewers. Radiology: Artif. Intell. *2*, e200029. Publisher: Radiological Society of North America.

39. Collins, G.S., Reitsma, J.B., Altman, D.G., and Moons, K.G.M. (2015). Transparent reporting of a multivariable prediction model for individual prognosis or diagnosis (TRIPOD): the TRIPOD Statement. BMC Med. *13*, 1.

40. Mitchell, M., Wu, S., Zaldivar, A., Barnes, P., Vasserman, L., Hutchinson, B., Spitzer, E., Raji, I.D., and Gebru, T. (2019). In Proceedings of the Conference on Fairness, Accountability, and Transparency (Association for Computing Machinery), pp. 220–229.

41. Athey, S., and Imbens, G.W. (2019). Machine Learning Methods That Economists Should Know About. Annu. Rev. Econom. *11*, 685–725. https://doi.org/10.1146/annurev-economics-080217-053433.

42. Schrider, D.R., and Kern, A.D. (2018). Supervised Machine Learning for Population Genetics: A New Paradigm. Trends Genet. *34*, 301–312.

43. Valletta, J.J., Torney, C., Kings, M., Thornton, A., and Madden, J. (2017). Applications of machine learning in animal behaviour studies. Anim. Behav. *124*, 203–220.

44. Iniesta, R., Stahl, D., and McGuffin, P. (2016). Machine learning, statistical learning and the future of biological research in psychiatry. Psychol. Med. *46*, 2455–2465. Publisher: Cambridge University Press.

45. Tonidandel, S., King, E.B., and Cortina, J.M. (2018). Big Data Methods: Leveraging Modern Data Analytic Techniques to Build Organizational Science. Organ. Res. Methods *21*, 525–547. Publisher: SAGE Publications Inc.

46. Yarkoni, T., and Westfall, J. (2017). Choosing Prediction Over Explanation in Psychology: Lessons From Machine Learning. Perspect. Psychol. Sci. *12*, 1100–1122. Publisher: SAGE Publications Inc.

47. Hofman, J.M., Watts, D.J., Athey, S., Garip, F., Griffiths, T.L., Kleinberg, J., Margetts, H., Mullainathan, S., Salganik, M.J., Vazire, S., et al. (2021). Integrating explanation and prediction in computational social science. Nature *595*, 181–188. Bandiera_abtest: a Cg_type: Nature Research Journals Number: 7866 Primary_atype: Reviews Publisher: Nature Publishing Group Subject_term: Interdisciplinary studies;Scientific community Subject_term_id: interdisciplinary-studies;scientificcommunity,.

48. McDermott, M.B.A., Wang, S., Marinsek, N., Ranganath, R., Foschini, L., and Ghassemi, M. (2021). Reproducibility in machine learning for health research: Still a ways to go. Sci. Transl. Med. *13*. Publisher: American Association for the Advancement of Science Section: Perspective. https://doi.org/10.1126/scitranslmed.abb1655.

49. Salganik, M.J., et al. (2020). Measuring the predictability of life outcomes with a scientific mass collaboration. Proc. Natl. Acad. Sci. USA *117*, 8398–8403. Publisher: National Academy of Sciences Section: Social Sciences.

50. Stodden, V., Seiler, J., and Ma, Z. (2018). An empirical analysis of journal policy effectiveness for computational reproducibility. Proc. Natl. Acad. Sci. USA *115*, 2584–2589.

51. Seibold, H., Czerny, S., Decke, S., Dieterle, R., Eder, T., Fohr, S., Hahn, N., Hartmann, R., Heindl, C., Kopper, P., et al. (2021). A computational reproducibility study of PLOS ONE articles featuring longitudinal data analyses. PLoS One *16*, e0251194.

52. Geirhos, R., Jacobsen, J.-H., Michaelis, C., Zemel, R., Brendel, W., Bethge, M., and Wichmann, F.A. (2020). Shortcut learning in deep neural networks. Nat. Mach. Intell. *2*, 665–673.

53. Chouldechova, A., Benavides-Prado, D., Fialko, O., and Vaithianathan, R. (2018). In In Proceedings of the 1st Conference on Fairness, Accountability and Transparency, pp. 134–148.

54. Kuhn, M., and Johnson, K. (2013). Applied Predictive Modeling (Springer-Verlag).

55. Filho, A.C., Batista, A.F.D.M., and Santos, H.G.d. (2021). Data Leakage in Health Outcomes Prediction With Machine Learning. Comment on “Prediction of Incident Hypertension Within the Next Year: Prospective Study Using Statewide Electronic Health Records and Machine Learning”. J. Med. Internet Res. *23*, e10969. Company: Journal of Medical Internet Research Distributor: Journal of Medical Internet Research Institution: Journal of Medical Internet Research Label: Journal of Medical Internet Research Publisher: JMIR Publications Inc., Toronto, Canada.

56. Oner, M.U., Cheng, Y.-C., Lee, H.K., and Sung, W.-K. (2020). Training Machine Learning Models on Patient Level Data Segregation Is Crucial in Practical Clinical Applications; Tech. rep., Company (Cold Spring Ha1rbor Laboratory Press Distributor: Cold Spring Harbor Laboratory Press Label: Cold Spring Harbor Laboratory Press Type: article), p. 2020.

57. Whalen, S., Schreiber, J., Noble, W.S., and Pollard, K.S. (2022). Navigating the pitfalls of applying machine learning in genomics. Nat. Rev. Genet. *23*, 169–181.

58. Roberts, D.R., Bahn, V., Ciuti, S., Boyce, M.S., Elith, J., Guillera-Arroita, G., Hauenstein, S., Lahoz-Monfort, J.J., Schröder, B., Thuiller, W., et al. (2017). Cross-validation strategies for data with temporal, spatial, hierarchical, or phylogenetic structure. Ecography *40*, 913–929.

59. Valavi, R., Elith, J., Lahoz-Monfort, J., and Guillera-Arroita, G. (2021). Block Cross-Validation for Species Distribution Modelling.

60. Malik, M.M.A. (2020). Hierarchy of Limitations in Machine Learning. Preprint at arXiv. https://doi.org/10.48550/arXiv.2002.05193.

61. Bone, D., Goodwin, M.S., Black, M.P., Lee, C.-C., Audhkhasi, K., and Narayanan, S. (2015). Applying Machine Learning to Facilitate Autism Diagnostics: Pitfalls and Promises. J. Autism Dev. Disord. *45*, 1121–1136.

62. Zech, J.R., Badgeley, M.A., Liu, M., Costa, A.B., Titano, J.J., and Oermann, E.K. (2018). Variable generalization performance of a deep learning model to detect pneumonia in chest radiographs: A cross-sectional study. PLoS Med. *15*. Publisher: Public Library of Science, e1002683.

63. Szegedy, C., Zaremba, W., Sutskever, I., Bruna, J., Erhan, D., Goodfellow, I., and Fergus, R. (2014). Intriguing properties of neural networks. Proceedings of the International Conference on Learning Representations.

64. Geirhos, R., Rubisch, P., Michaelis, C., Bethge, M., Wichmann, F.A., and Brendel, W. (2018). ImageNet-trained CNNs are biased towards texture; increasing shape bias improves accuracy and robustness. Proceedings of the International Conference on Learning Representations. https://openreview.net/forum?id=Bygh9j09KX.

65. Carter, B., Jain, S., Mueller, J., and Gifford, D. (2021). Overinterpretation reveals image classification model pathologies. Adv. Neural Inf. Process. Syst.

66. Recht, B., Roelofs, R., Schmidt, L., and Shankar, V. (2019). Do ImageNet Classifiers Generalize to ImageNet? In In Proceedings of the 36th International Conference on Machine Learning, pp. 5389–5400. https://proceedings.mlr.press/v97/recht19a.html.

67. Paullada, A.; Raji, I. D.; Bender, E. M.; Denton, E.; Hanna, A. Data and its (dis) contents: A survey of dataset development and use in machine learning research.Preprint at arXiv https://doi.org/10.1016/j.patter.2021.100336preprint arXiv:2012.05345 2020.

68. Scheuerman, M.K., Hanna, A., and Denton, E. (2021). Do Datasets Have Politics? Disciplinary Values in Computer Vision Dataset Development. Proc. ACM Hum. Comput. Interact. *5*, 1–37.

69. Gebru, T., Morgenstern, J., Vecchione, B., Vaughan, J.W., Wallach, H., Iii, H.D., Crawford, K., and Crawford, K. (2021). Datasheets for datasets. Commun. ACM *64*, 86–92.

70. Han, S., Olonisakin, T.F., Pribis, J.P., Zupetic, J., Yoon, J.H., Holleran, K.M., Jeong, K., Shaikh, N., Rubio, D.M., and Lee, J.S. (2017). A checklist is associated with increased quality of reporting preclinical biomedical research: A systematic review. PLoS One *12*, e0183591.

71. Garbin, C., and Marques, O. (2022). Assessing Methods and Tools to Improve Reporting, Increase Transparency, and Reduce Failures in Machine Learning Applications in Health Care. Radiol. Artif. Intell. *4*, e210127.

72. Raji, D., Denton, E., Bender, E.M., Hanna, A., and Paullada, A. (2021). AI and the Everything in the Whole Wide World Benchmark. Proceedings of the Neural Information Processing Systems Track on Datasets and Benchmarks, 1.

73. Lundberg, I., Johnson, R., and Stewart, B.M. (2021). What Is Your Estimand? Defining the Target Quantity Connects Statistical Evidence to Theory. Am. Socio. Rev. *86*, 532–565.

74. Liu, D.M., and Salganik, M.J. (2019). Successes and Struggles with Computational Reproducibility: Lessons from the Fragile Families Challenge. Socius. *5*, 237802311984980.

75. Muchlinski, D., Siroky, D., He, J., and Kocher, M. (2016). Comparing Random Forest with Logistic Regression for Predicting Class-Imbalanced CivilWar Onset Data. Polit. Anal. *24*, 87–103. Publisher: Cambridge University Press.

76. Colaresi, M., and Mahmood, Z. (2017). Do the robot: Lessons from machine learning to improve conflict forecasting. J. Peace Res. *54*, 193–214. Publisher: SAGE Publications Ltd.

77. Wang, Y. Comparing Random Forest with Logistic Regression for Predicting Class-Imbalanced Civil War Onset Data: A Comment. Political Analysis 2019, 27, Publisher: Cambridge University Press, 107–110.

78. Kaufman, A.R., Kraft, P., and Sen, M. (2019). Improving Supreme Court Forecasting Using Boosted Decision Trees. Political Analysis27 (Cambridge University Press), pp. 381–387.

79. Bara, C. (2020). Forecasting civil war and political violence, pp. 177–193. Publication Title: The Politics and Science of Prevision; Routledge.

80. (2020). Scimago Journal and Country Rank. http://archive.today/oUs4K.

81. Ward, M.D., Greenhill, B.D., and Bakke, K.M. (2010). The perils of policy by p-value: Predicting civil conflicts. J. Peace Res. *47*, 363–375. Publisher: SAGE Publications Ltd.

82. Breiman, L. (2001). Statistical Modeling: The Two Cultures (with comments and a rejoinder by the author). Stat. Sci. *16*, 199–231. Publisher: Institute of Mathematical Statistics.

83. Dressel, J., and Farid, H. (2018). The accuracy, fairness, and limits of predicting recidivism. Sci. Adv. *4*, eaao5580.

84. Olson, R.S., Cava, W.L., Mustahsan, Z., Varik, A., and Moore, J.H. (2018). Data-driven advice for applying machine learning to bioinformatics problems. Pacific Symposium on Biocomputing. Pac. Symp. Biocomput. *23*, 192–203.

85. Gorman, K., and Bedrick, S. (2019). We Need to Talk about Standard Splits. In In Proceedings of the 57th Annual Meeting of the Association for Computational Linguistics (Association for Computational Linguistics: Florence, Italy), pp. 2786–2791. https://aclanthology.org/P19-1267/.

86. Blair, R.A., and Sambanis, N. (2020). Forecasting Civil Wars: Theory and Structure in an Age of "Big Data" and Machine Learning. J. Conflict Resolut. *64*, 1885–1915. Publisher: SAGE Publications Inc.

87. Robin, X., Turck, N., Hainard, A., Tiberti, N., Lisacek, F., Sanchez, J.-C., and Müller, M. (2011). pROC: an open-source package for R and S+ to analyze and compare ROC curves. BMC Bioinf. *12*, 77.

88. He, K., Zhang, X., Ren, S., and Sun, J. (2015). Delving Deep into Rectifiers: Surpassing Human-Level Performance on ImageNet Classification. CoRR.

89. Szeliski, R. (2021). Computer Vision: Algorithms and Applications, 2nd ed. https://szeliski.org/Book.

90. Shi, L., and Lin, L. (2019). The trim-and-fill method for publication bias: practical guidelines and recommendations based on a large database of meta-analyses. Medicine *98*, e15987.

91. Gurevitch, J., Koricheva, J., Nakagawa, S., and Stewart, G. (2018). Meta-analysis and the science of research synthesis. Nature *555*, 175–182. Bandiera_abtest: a Cg_type: Nature Research Journals Number: 7695 Primary_atype: Reviews Publisher: Nature Publishing Group Subject_term: Biodiversity;Outcomes research Subject_term_id: biodiversity;outcomes-research.

92. Hofman, J.M., Sharma, A., and Watts, D.J. (2017). Prediction and explanation in social systems. Science *355*, 486–488. Publisher: American Association for the Advancement of Science Section: Essays.

93. Islam, R., Henderson, P., Gomrokchi, M., and Precup, D. (2017). Reproducibility of Benchmarked Deep Reinforcement Learning Tasks for Continuous Control.

94. Lones, M. A. How to avoid machine learning pitfalls: a guide for academic researchers.Preprint at arXiv:2108.02497 [cs] 2021,https://doi.org/10.48550/arXiv.2108.02497 arXiv: 2108.02497.

95. Russakovsky, O., Deng, J., Su, H., Krause, J., Satheesh, S., Ma, S., Huang, Z., Karpathy, A., Khosla, A., Bernstein, M., et al. (2015). ImageNet Large Scale Visual Recognition Challenge. Int. J. Comput. Vis. *115*, 211–252.

96. Koh, P.W., et al. (2021). In In Proceedings of the 38th International Conference on Machine Learning, pp. 5637–5664.

97. Rocca, R., and Yarkoni, T. (2021). Putting Psychology to the Test: Rethinking Model Evaluation Through Benchmarking and Prediction. Advances in Methods and Practices in Psychological Science *4*, 251524592110268.

98. Donoho, D. (2017). 50 Years of Data Science. J. Comput. Graph Stat. *26*, 745–766. Publisher: Taylor & Francis _eprint:. https://doi.org/10.1080/10618600.2017.1384734

99. Marie, B., Fujita, A., and Rubino, R. (2021). Scientific Credibility of Machine Translation Research: A Meta-Evaluation of 769 Papers. In Proceedings of the 59th Annual Meeting of the Association for Computational Linguistics and the 11th International Joint Conference on Natural Language Processing, pp. 7297–7306. https://doi.org/10.48550/arXiv.2106.

100. Clyburne-Sherin, A., Fei, X., and Green, S.A. (2019). Computational reproducibility via containers in social psychology. Meta-Psychology *3*.

101. (2018). Easing the burden of code review. Nat. Methods *15*, 641. Bandiera_abtest: a Cg_type: Nature Research Journals Number: 9 Primary_atype: Editorial Publisher: Nature Publishing Group Subject_term: Computational biology and bioinformatics;Publishing Subject_term_id: computationalbiology-and-bioinformatics;publishing.

102. Hutson, M. (2019). No coding required: Companies make it easier than ever for scientists to use artificial intelligence. Science.

103. Kapoor, S., and Narayanan, A. (2021). Claims of Superior Performance of Machine Learning over Logistic Regression for Civil War Prediction Don't Reproduce. https://www.codeocean.com/, version v1.

104. Hook, D.W., Porter, S.J., and Herzog, C. (2018). Dimensions: Building Context for Search and Evaluation. Frontiers in Research Metrics and Analytics *3*. Publisher: Frontiers. https://doi.org/10.3389/frma.2018.00023.

Patterns, Volume *4*

Supplemental information

Leakage and the reproducibility crisis in machine-learning-based science

Sayash Kapoor and Arvind Narayanan

Supplementary Information: Leakage and the Reproducibility Crisis in ML-based Science

Supplemental Experimental Procedures.

Overview of the Appendix. In Section S1, we justify our choice of the word reproducibility. In Section S2, we provide a detailed description of the methods we used to select papers for our review of civil war prediction and fix reproducibility issues in the papers with errors. In Section S3, we show how model info sheets address each type of leakage identified in our survey. In Section S4, we include a template for the model info sheets.

We include a list of all 124 papers that we considered for our literature review on civil war prediction as supplementary documents with this submission.

S1 Why do we call these reproducibility issues?

We acknowledge that there isn't consensus about the term reproducibility, and there have been a number of recent attempts to define the term and create consensus[1]. One possible definition is computational reproducibility—when the results in a paper can be replicated using the exact code and dataset provided by the authors[2]. We argue that this definition is too narrow because even cases of outright bugs in the code would not be considered irreproducible under this definition. Therefore we advocate for a standard where bugs and other errors in data analysis that change or challenge a paper's findings constitute irreproducibility.

The goal of predictive modeling is to estimate (and improve) the accuracy of predictions that one might make in a real-world scenario. This is true regardless of the specific research question one wishes to study by building a predictive model. In practice one sets up the data analysis to mimic this real-world scenario as closely as possible. There are limits to how well we can do this and consequently, there is always methodological debate on some issues, but there are also some clear rules. If an analysis choice can be shown to lead to incorrect estimates of predictive accuracy, there is usually consensus in the ML community that it is an error. For example, violating the train-test split (or the learn-predict separation) is an error because the test set is intended to provide an accurate estimate of 'out-of-sample' performance—model performance on a dataset that was not used for training[3]. Thus, to define what is an error, we look to this consensus in the ML community (e.g. in textbooks) and offer our own arguments when necessary.

S2 Materials and Methods: Reproducibility issues in civil war prediction

Different researchers might have different aims when comparing the performance on civil war prediction — determining the absolute performance, or comparing the relative performance of different models of civil war prediction. Whether the aim is to determine the relative or absolute performance of models of civil war prediction, data leakage causes a deeper issue in the findings of each of the 4 papers with errors that leads to inaccurate estimates of both relative and absolute out-of-sample performance.

In correcting the papers with errors[4–7], our aim is to report out-of-sample performance of the various models of civil war prediction after correcting the data leakage, while keeping all other factors as close to the original implementation as possible. Fixing the errors allows a more accurate estimate of out-of-sample performance.

At the same time, we caution that just because our corrected results offer a more accurate estimate of out-of-sample performance doesn't mean that we endorse all other methodological choices made in the papers. For example, to correct the results reported by Muchlinski et al.[4], we use imputation on an out-of-sample dataset that has 95% missing values. While an imputation model created only using the training data avoids data leakage, it does not mean that using a dataset with 95% missing values to measure out-of-sample performance is desirable.

S2.1 Paper selection for review

To find relevant papers on civil war prediction for our review, we used the search results from a dataset of academic literature[8] for papers with the terms *'civil' AND 'war' AND ('prediction' OR 'predicting' OR 'forecast')* in their title or abstract, as well as papers that were cited in a recent review of the field[9]. To keep the number of papers tractable, we limited ourselves to those that were published in the last 5 years, specifically, papers published between 1st January 2016 and 14th May 2021. This yielded 124 papers. We narrowed this list to the 15 papers that were focused on predicting civil war and evaluated performance using a train-test split. Of the 15 papers that meet our inclusion criteria, 12 share the complete code and data. For these 12, we attempted to identify errors and reproducibility issues from the text and through reviewing the code provided with the papers. When we identified errors, we re-analyzed the data with the errors corrected. We now address the reproducibility issues we found in each paper in detail.

S2.2 Muchlinski et al.[4]

Imputation is commonly used to fill in missing values in datasets[10]. Imputing the training and test datasets together refers to using data from the training as well as the test datasets to create an imputation model that fills in all missing values in the dataset. This is an erroneous imputation method for the predictive modeling paradigm, since it can lead to data leakage, which results in incorrect, over-optimistic performance claims. This pitfall is well known in the predictive modeling community — discussed in ML textbooks[3], blogs[11] and popular online forums[12].

Muchlinski et al.[4] claim that a Random Forests model vastly outperforms logistic regression models in terms of out-of-sample performance using the AUC metric[13]. However, since they impute the training and test datasets together, their results suffer from data leakage. The impact of leakage is especially severe because of the level of missingness in their out-of-sample test dataset: over 95% of the values are missing (which is not reported in the paper), and 70 of the 90 variables used in their model are missing for *all* instances in the out-of-sample test set.[1] When their imputation method is corrected, their Random Forests model performs no better than the logistic regression models that they compared against.

We focus on reproducing the out-of-sample results reported by Muchlinski et al.[4]. Table S1 provides the comparisons between the results reported in Muchlinski et al.[4], our reproductions of their reported (incorrect) results, as well as the corrected version of their results. Muchlinski et al.[4] received two critiques of the methods used in their paper[6,14]. [2]. In response, they published a reply with clarifications and revised code addressing both critiques[16]. We use the revised version of their code. We find that the error in their imputation methods exists in the revised code as well as the original code, and was not identified by the previous critiques. Muchlinski et al.[4] re-use the dataset from Hegre and Sambanis[17] when training their models, and provide a separate out-of-sample test set for evaluation. To address missing values, they use a Random Forests based imputation method in R called *rfImpute*. However, the training and test sets are imputed together, which leads to a data leakage. This results in overoptimistic performance claims. Below, we detail the steps we take to correct their results, provide a visualization of the data leakage, and provide a simulation showcasing how the data leakage can result in overoptimistic claims of performance.

Correcting the data imputation. To correct this error, we use the *mice* package in R which uses multiple imputation for imputing missing data. This is because the *mice* package allows us to specify which rows in the dataset are a part of the test set and it does not use those rows for creating the imputation

[1]While leakage is particularly serious in predictive modeling, a dataset with 95% of values missing is problematic even for explanatory modeling.

[2]Hofman et al.[15] also outline the shortcomings in the initial code released by Muchlinski et al.[4].

model, whereas *rfImpute* — the original method used to impute the missing data in the original results by Muchlinski et al.[4] — does not have this feature. The authors imputed the training set together with the out-of-sample test set using *rfImpute*, which led to data leakage. Table S1 provides the comparisons between the results reported in Muchlinski et al.[4], our reproductions of their reported (incorrect) results, as well as the corrected version of their results.

Using multiple imputation fills in missing values without regarding the underlying variable's original distribution. For example, using multiple imputation fills in different missing values for the variable representing the percentage of rough terrain in a country in different years[18], whereas this particular variable (percentage of rough terrain) is constant over time. However, when multiple imputation is used with a train-test split, there is still no leakage between the training and test sets, since the imputation model only uses data from the training set to fill in missing values in the test set.

Why can't we use *rfImpute* in the corrected results? Instead of using the *mice* package, another way to impute the data correctly, i.e., without data leakage, would be to run the imputation using *rfImpute* on the training and test data separately — creating two separate imputation models — one for the training data and one for the test data. We could not use this imputation method because 70 of the 90 variables used in Muchlinski et al.[4]'s model as features do not have *any* values in the out-of-sample test data provided — i.e. they are missing for *all* observations in the out-of-sample dataset — and *rfImpute* requires at least some values for each variable to not be missing. In other words, the *mice* package allows us to train an imputation model on the training set and use it to fill in missing values in the test set.

Subtle differences between explanatory and predictive modeling. In the explanatory modeling paradigm, the aim is to draw inferences from data, as opposed to optimizing and evaluating out-of-sample predictive performance. In this case, data imputation would be considered a part of the data pre-processing step, even though it is still important to keep in mind the various assumptions being made in this process Schafer[19]. Contrarily, in the predictive modeling paradigm, the imputation is a part of the modeling step[3] because the aim of the modeling exercise is to validate performance on an out-of-sample test set, which the model does not have access to during the training. In this case, imputing the training and test datasets together leads to leaking information from the test set to the training set and thus the performance evaluation on the purportedly "out-of-sample" test set would be an over-estimate.

What is the precise mechanism by which the leakage occurs in Muchlinski et al.[4]? When Muchlinski et al.[4] impute the missing values in the out-of-sample test set, the imputation model has access to the entire training data as well as the labels of the target variables in the test data — they also include the target variable in the list of variables which the imputation model treats as independent variables when carrying out the imputation. The model therefore uses correlations between the target variable and independent variables in the training dataset and uses them to fill in the missing values in the test dataset — i.e. the model uses the labels of the target variables in the test data and correlations from the training data to fill in missing values. This leads to the test dataset having similar correlations between the target and independent variables as the ones present in the training data. Further, the missing data is filled in in such a way that it favors ML models such as Random Forests over logistic regression models, as we show in the visualization below.

Visualizing the leakage. We can visually observe an instance of data leakage in Figure S1. We focus on the distribution of the feature *agexp*, which represents the proportion of agricultural exports in the GDP of a country. We choose this feature because in the Muchlinski et al. paper, this feature had the highest gini index for the random forests model — which means that it was an important feature for the model. While we only visualize one feature here, similar results hold across multiple features used in the model. Below, we reconstruct the process by which the data leakage was generated — following the exact steps Muchlinski et al.[4] used to create and evaluate the dataset:

- Figure S1a represents the distribution of the *agexp* variable for war and peace data points in the original dataset by Hegre and Sambanis[17], ignoring missing values.

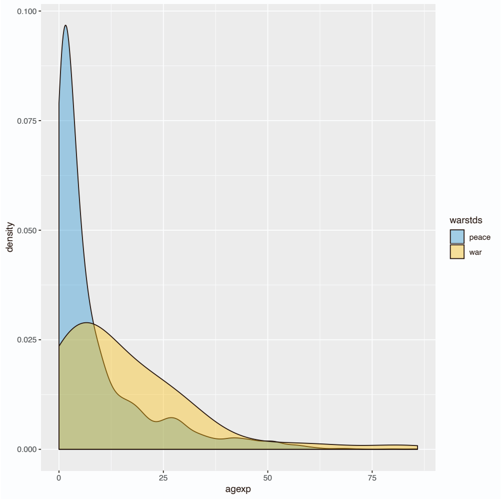

(a) Distribution of the *agexp* variable for peace and war data points for the original Hegre et al. dataset, ignoring missing values

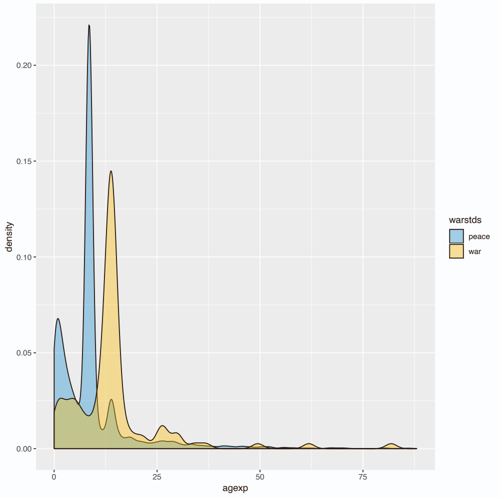

(b) Distribution of the *agexp* variable for peace and war data points for the imputed Hegre et al. dataset used by Muchlinski et al. for training

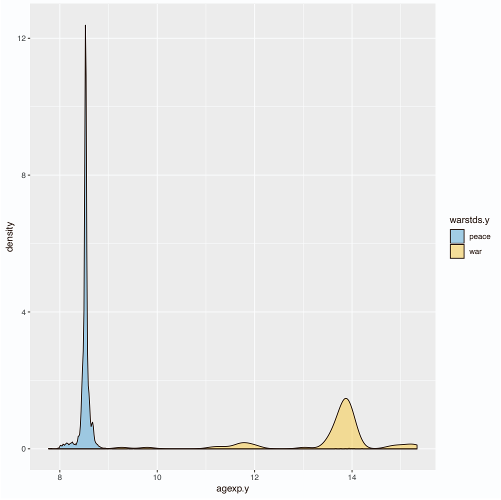

(c) Distribution of the *agexp* variable for peace and war data points only for the data points that were added during imputation (i.e. the data points that were missing in the original dataset)

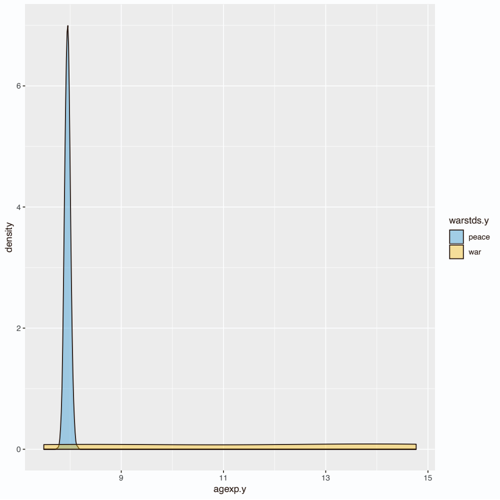

(d) Distribution of the *agexp* variable for peace and war data points for the out-of-sample test set

Figure S1: Distribution of the *agexp* variable for peace and war data points for different imputation steps in Muchlinski et al.[4]. Note that the distribution of *peace* instances in the test set (D) has a peak that is close to the distribution in the imputed training set (B, C) — which allows the random forests model to learn the small range of values where *peace* data points are concentrated. While we report results for the *agexp* variable, similar trends appear across independent variables in the dataset.

- Figure S1b shows the same distribution after including the imputed values of *agexp*. In particular, we see two peaks in the dataset for war and peace data points alike, one due to war instances and one due to peace instances.
- If we look only at the data points that were imputed using the *rfImpute* method (Figure S1c), we see that the distribution of the imputed data points for war and peace are completely separated, in contrast to the original distribution where there was a significant overlap between the distributions.
- Finally, Figure S1d shows the effect of imputing this already-imputed dataset with the out-of-sample test set — we see that the out-of-sample dataset only has the peak for peace datapoints, whereas the distribution for war is almost uniform.

Further, the random forests model can learn the peak for the *agexp* variable in the *peace* instances from the training dataset after imputation, since the peak for the training and test sets is similar. It can distinguish between war and peace datapoints much more easily compared to a logistic regression model that only uses one parameter per feature — logistic regression models are monotonic functions of the independent variables and therefore cannot learn that a variable only lies within a small range for a given label. This highlights the reason behind Random Forests outperforming logistic regression in this setting — imputing the training and test datasets together leads to variable values being artifically concentrated within a very small range for both the training and test datasets — and further, being neatly separated across *war* and *peace* instances. The impact of the imputation becomes even clearer when we consider that the out-of-sample test dataset provided by Muchlinski et al.[4] has over 95% of the data missing, and 70 out of 90 variables are missing for all instances in the out-of-sample dataset.

A simulation showcasing the impact of missingness on performance estimates in the presence of leakage. We can observe a visual example of how data leakage affects performance evaluation in Figure S2. We describe the simulation below:

- there are two variables — the target variable *onset* and the independent variable *gdp*.
- *onset* is a binary variable. *gdp* is drawn from a normal distribution and depends on *onset* as follows:

$$gdp = N(0, 1) + onset.$$

- We generate 1000 samples with *onset=0* and 1000 samples with *onset=1* to create the dataset.
- We randomly split the data into training (50%) and test (50%) sets, and create a random forests model that is trained on the training set and evaluated on the test set.
- To observe the impact of imputing the training and test sets together, we randomly delete a certain percentage of values of *gdp*, and impute it using the imputation method used in Muchlinski et al.[4].
- We vary the proportion of missing values from 0% to 95% in increments of 5% and plot the accuracy of the random forests classifier on the test set.
- We run the entire process 100 times and report the mean and 95% CI of the accuracy in Figure S2; the 95% CI is too small to be seen in the Figure.

We find that imputing the training and test sets together leads to an increasing improvement in the purportedly "out-of-sample" accuracy of the model. Estimates of model performance in this case are artificially high. This example also highlights the impact of the high percentage of missing values — since the out-of-sample test set used by Muchlinski et al.[4] contains over 95% missing values, the impact of imputing the training and test sets together is very high.

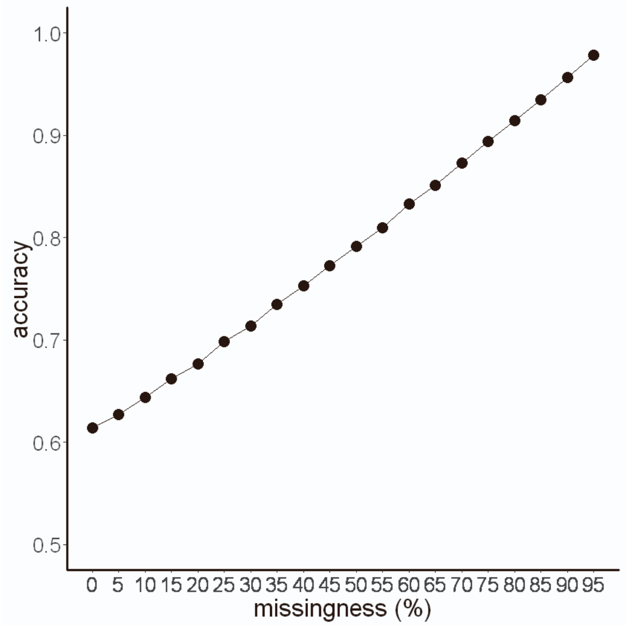

Figure S2: Results of a simulation that showcase how imputing the training and test sets together leads to overoptimistic estimates of model performance. The 95% Confidence Intervals are too small to be seen.

Algorithm	Reported	Reported results (reproduced)	Corrected results
Fearon and Laitin	0.69	0.78	0.54
Collier and Hoeffler	0.90	0.83	0.57
Hegre and Sambanis	0.83	0.82	0.68
Muchlinski et al.	0.94	0.95	0.64

Table S1: Original and corrected results in Muchlinski et al.[4]. While there are differences between the reported results and our reproduction of the reported results, especially for the Fearon and Laitin as well as the Collier and Hoeffler models, the relative order of the model performance for both results is the same.

S2.3 Colaresi and Mahmood[5]

Colaresi and Mahmood[5] report that ML models vastly outperform logistic regression for predicting civil war onset. However, they re-use the imputed version of the dataset in Hegre and Sambanis[17] which is provided by Muchlinski et al.[4]. They use the imputed dataset both for training and testing via a train-test split; they do not use the out-of-sample test set provided by Muchlinski et al. This means that the results in Colaresi and Mahmood[5] are subject to exactly the same pitfall as in Muchlinski et al.[4], albeit with a slightly different dataset. Correcting the imputation method dramatically reduces the performance of the ML models proposed.

We focus on reproducing the final round of results reported in the paper Colaresi and Mahmood[5], which consists of a comparison of 3 models of civil war onset — the Random Forests model proposed in Muchlinski et al.[4], the Random Forests model proposed in Colaresi and Mahmood[5] as well as the logistic regression model proposed in Fearon and Laitin[20]. Their dataset has 17.4% values missing, and the test set has 19% values missing. The proportion of missing values in individual variables can be even higher — for example, the *agexp*, which represents the proportion of agricultural exports in the GDP of a country, is missing for 54.3% of the rows in the test set. In our corrected results, we use the original dataset from Hegre and Sambanis[17] and impute the training and test data separately using the *rfImpute* function. The test set consists of data from the years after 1988. One of the independent variables, *milper*, is missing for all instances in the test set of Colaresi and Mahmood[5] so we exclude this variable from our models. Table S2 provides the comparisons between the results reported in Colaresi and Mahmood[5], our reproductions of their

Algorithm	Reported	Reported results (reproduced)	Corrected results
Fearon and Laitin	0.77	0.77	0.79
Muchlinski et al.	0.89	0.89	0.73
Colaresi and Mahmood	0.91	0.91	0.75

Table S2: Original results from Colaresi and Mahmood[5] and our corrected results.

reported (incorrect) results, as well as the corrected version of their results.

Colaresi and Mahmood[5] and Wang[6] reuse the dataset released by Muchlinski et al.[4]. This is the imputed version of the dataset released by Hegre and Sambanis[17]. However, for 777 rows in the imputed dataset released by Muchlinski et al.[4], the original dataset by Hegre and Sambanis[17] has a missing target variable (i.e. the variable representing civil war onset is missing) whereas the imputed version of the dataset (i.e. the dataset released by Muchlinski et al.[4]) has a value of *peace* for the target variable representing civil war onset. Since Muchlinski et al.[4] do not share the code that they use for imputing the Hegre and Sambanis[17] dataset, it is unclear how the missing values in the target variable were imputed in the dataset, especially since the imputation method they use — *rfImpute* — requires non-missing values in the target variable. Still, the number of instances of civil war onset (i.e. instances where the variable representing civil war onset has the value *war*) in the Hegre and Sambanis[17] dataset as well as the Muchlinski et al.[4] dataset are the same.

S2.4 Wang[6]

Similar to Colaresi and Mahmood[5], Wang[6] report that ML models vastly outperform logistic regression for predicting civil war onset. However, they too re-use the imputed version of the dataset in Hegre and Sambanis[17] provided by Muchlinski et al.[4]. They use the imputed dataset both for training and testing via k-fold cross-validation; they do not use the out-of-sample test set provided by Muchlinski et al. Correcting the imputation method dramatically reduces the performance of the ML models proposed.

We focus on reproducing the results of the nested cross-validation implementation reported by Wang[6]. Wang[6] reuses the imputed dataset provided by Muchlinski et al.[4], instead of using the original dataset provided by Hegre and Sambanis[17] and imputing the training and test sets separately. The dataset has 17.4% values missing. The proportion of missing values in individual variables can be even higher — for example, the *agexp*, which represents the proportion of agricultural exports in the GDP of a country, is missing for 49.8% of the rows in the data set. In our corrected results, we use the original dataset from Hegre and Sambanis[17] and impute the training and test data separately using the *rfImpute* function within each cross validation fold. This ensures that there is no data leakage between the training and test sets in each fold. Table S3 provides the comparisons between the results reported in Wang[6], our reproductions of their reported (incorrect) results, as well as the corrected version of their results.

We also conduct an additional robustness analysis in which we use a separate out-of-sample test set instead of $k-$fold cross validation, since using $k-$fold cross validation with temporal data can also lead to leakage across the train-test split. To maintain comparability between the original and corrected results by testing on the same instances of civil war, we continue to use $k-$fold cross-validation in the corrected results in Figure 2. We report the results after making this change in Table S3. We use the same train-test split as Colaresi and Mahmood[5] — $year < 1988$ as training data and the rest as test data — for the out-of-sample test set. The test set consists of data from the years after 1988. One of the independent variables, *milper*, is missing for all instances in the test set of Colaresi and Mahmood[5] so we exclude this variable from our models.

Note that the imputation method that should be used depends on the exact model deployment scenario, and should mimic it as closely as possible for accurate performance estimates. For example, in some model deployment settings samples for prediction come in one at a time and in some cases they come in batches. In the former setting, imputing the entire test set together may result in overoptimistic performance evaluations as well, since the deployed model doesn't have access to a batch of samples. Our results may thus offer an upper bound on the performance of civil war prediction models in the case of Colaresi and Mahmood[5] and Wang[6].

Algorithm	Reported	Reported (reproduced)	k-fold CV (corrected)	Out-of-sample (corrected)
Fearon and Laitin	0.76	0.76	0.77	0.78
Collier and Hoeffler	0.78	0.78	0.72	0.77
Hegre and Sambanis	0.80	0.80	0.81	0.80
Muchlinski et al.	0.92	0.92	0.78	0.73
AdaBoost	0.94*	0.94	0.82	0.77
GBT	0.94*	0.94	0.81	0.75

Table S3: Original and corrected results in the Wang[6]. We find that using an out-of-sample test set further favors logistic regression models over ML models. The metric for all results is AUC. *These results were not reported using nested cross-validation in Wang[6]. In our reproduction of these reported results, we use nested cross-validation, which ensures that we do not get over-estimates of performance.

S2.5 Kaufman, Kraft, and Sen[7]

We focus on reproducing the results on civil war prediction in Kaufman, Kraft, and Sen[7]. There are several issues in the paper's results. We outline each issue below and provide a comparison of various scenarios in Table S4 that highlight the precise cause of the performance difference between the original and corrected results, and visualize the robustness of our corrected results. We find that even though there are several issues in Kaufman, Kraft, and Sen[7], the main difference in performance between the original results they report and our corrected results is due to data leakage.

Data leakage due to proxy variables. The dataset used by Kaufman, Kraft, and Sen[7] has several variables that, if used as independent variables in models of civil war prediction, could cause data leakage, since they are proxies of the outcome variable. Table S5 lists the variables in the Fearon and Laitin[20] dataset that cause leakage. The first 4 rows outline variables that could be affected by civil wars, as outlined in Fearon and Laitin[20]. Therefore, following Fearon and Laitin[20], we use lagged versions of these variables in our correction. The other variables in Table S5 are either direct proxies of outcomes of interest or are missing for all instances for civil war.

Parameter selection for the Lasso model. Kaufman, Kraft, and Sen[7] use an incorrect parameter selection technique when creating their Lasso model that leads to the model always predicting *peace* (i.e. all coefficients of the variables in the model are always zero). We correct this using a standard technique for parameter selection. Instead of choosing model parameters such that the model always predicts *peace*, we use the *cv.glmnet* function in R to choose a suitable value for model parameters based on the training data.

Using $k-$fold cross-validation with temporal data. $k-$fold cross-validation shuffles the dataset before it is divided into training and test datasets. When the dataset contains temporal data, the training dataset could contain data from a later date than the test dataset because of being shuffled. To maintain comparability between the original and corrected results by testing on the same instances of civil war, we continue to use $k-$fold cross-validation in the corrected results in Figure 2. To evaluate out-of-sample performance without using cross-validation, we use a separate train-test split instead of $k-$fold cross-validation and report the difference in results for this scenario in the row *Corrected (out-of-sample)* in Table S4. We find that there is no substantial difference between the results when using the out-of-sample test set and $k-$fold cross-validation — in each case, none of the models outperforms a baseline that predicts the outcome of the previous year. We use the same train-test split as Colaresi and Mahmood[5] — *year* $<$ *1988* as training data and the rest as test data.

Replacing missing values with zeros. Kaufman, Kraft, and Sen[7] replace missing values in their dataset with zeros, instead of imputing the missing data or removing the rows with missing values. This is a methodologically unsound way of dealing with missing data: for example, the models would not be able to discern whether a variable has a value of zero because of missing data or because it was the true value of the variable for that instance. This risks getting underestimates of performance, as opposed to overoptimistic

performance claims. As a robustness check, we impute the training and test data separately in each cross-validation fold using the *rfImpute* function in R and report the results in the *Corrected (imputation)* row of Table S4. We find that the choice of imputation method does not cause a difference in performance, perhaps because only 0.6% of the values of variables are missing in the dataset.

Choice of cut-offs for calculating accuracy. Instead of calculating model cutoffs based on the best cutoff in the training set, Kaufman et al. use the distribution of model scores to decide the cutoffs for calculating accuracy. We include robustness results when we change the cutoff selection procedure to choosing the best cutoffs for the training set in the *Corrected (cutoff choice)* row of Table S5. We find that the choice of cutoff does not impact the main claim — the performance of the best model is still worse than a baseline that predicts the outcome of the previous year.

Weak Baseline. Kaufman, Kraft, and Sen[7] compare their results against a baseline model that always predicts *peace*. We find that a baseline that predicts *war* if the outcome of the target variable was civil war in the previous year and predicts *peace* otherwise is a stronger baseline (Accuracy: 97.5% vs. 86.1%; χ^2=633.7, $p = 7.836e\text{-}140$ using McNemar's test as detailed in Dietterich[21]), and report results against this stronger baseline in Table S4.

Confusion about the target variable. Kaufman, Kraft, and Sen[7] use ongoing civil war instead of civil war onset as the target variable in their models. While their abstract mentions that the prediction task they attempt is civil war onset prediction, they switch to using the term *civil war incidence* in later sections, without formally defining this term. To attempt to determine what they mean by this term, we looked at the papers they cite; one of them has the term *civil war incidence* in the title Collier and Hoeffler[22], and defines civil war incidence as 'observations [that] experienced a start of a civil war'. At the same time, in the introduction, they state that they are 'predicting whether civil war occurs in a country in a given year' — which refers to ongoing civil war instead of civil war onset. This might confuse a reader about the specific prediction task they undertake.

Scenario	ADT	RF	SVM	ERF	Lasso	LR	Baseline	Stronger Baseline
Reported	0.990	0.989	0.983	0.990	0.862	0.987	0.861	0.000
Reported (reproduction)	0.990	0.990	0.983	0.989	0.861	0.987	0.861	0.000
Corrected	0.974	0.959	0.974	0.957	0.975	0.972	0.861	0.975
Corrected (out-of-sample)	0.966	0.936	0.962	0.927	0.966	0.963	0.796	0.966
Corrected (imputation)	0.974	0.959	0.974	0.957	0.975	0.975	0.861	0.975
Corrected (cutoff choice)	0.974	0.972	0.966	0.967	0.975	0.971	0.861	0.975

Table S4: Results for the various scenarios in Kaufman, Kraft, and Sen[7]. We report results up to 3 significant figures in this table because the small difference in performance between AdaBoost and logistic regression that is ascribed signifance in Kaufman, Kraft, and Sen[7] can only be observed in the third decimal point. The first 2 values of 'Stronger Baseline' are reported as 0 because this baseline was not included in the results of Kaufman, Kraft, and Sen[7].

S2.6 Blair and Sambanis[23]

Blair and Sambanis[23] state that their *escalation* model outperforms other models across a variety of settings. However, they do not test the performance evaluations to see if the difference is statistically significant. We find that there is no significant difference between the smoothed AUC values of the *escalation* model's performance and other models they compare it to when we use a test for significance. Further, we provide a visualization of the 95% confidence intervals of specificities and sensitivities in the smoothed ROC curve they report for their model (*escalation*) as well as for a baseline model (*cameo*) — and find that the 95% confidence intervals are large (see Figure S3).

Variable name	Reason for leakage	Variable definition in data documentation
pop	affected by target variable	population; in 1000s
lpop	affected by target variable	log of population
polity2	affected by target variable	revised polity score
gdpen	affected by target variable	gdp/pop based on pwt5.6; wdi2001;cow energy data
onset	codes civil war onset	1 for civil war onset
ethonset	codes civil war onset	1 if onset = 1 and ethwar ~= 0
durest	NA if onset = 0	estimated war duration
aim	NA if onset = 0	1 = rebels aim at center; 3 = aim at exit or autonomy; 2 = mixed or ambig.
ended	NA if onset = 0	war ends = 1; 0 = ongoing
ethwar	NA if onset = 0	0 = not ethnic; 1 = ambig/mixed; 2 = ethnic
emponset	codes civil war onset	onset coded for data with empires
sdwars	codes ongoing civil war	Number of Sambanis/Doyle civ wars in progress
sdonset	codes civil war onset	onset of Sambanis/Doyle war
colwars	codes ongoing civil war	Number of Collier/Hoeffler wars in progress
colonset	codes civil war onset	onset of Collier/Hoeffler war
cowwars	codes ongoing civil war	Number of COW civ wars in progress
cowonset	codes civil war onset	onset of COW civ war

Table S5: This table highlights the variables included as independent variables in Kaufman, Kraft, and Sen[7] which cause a data leakage. In the original use of the dataset, Fearon and Laitin[20] include lagged versions of the first 4 variables in the list as independent variables in their model to avoid leakage. Following their use of lagged versions of these variables, we do the same in our correction to avoid leakage. The other variables are proxies for the outcomes of interest and hence we remove them from the models to avoid data leakage.

Uncertainty quantification, p-values and Z-values for tests of statistical significance.

- We report p-values and Z values for a one-tailed significance test comparing the smoothed AUC performance of the *escalation* model with other baseline models reported in their paper — *quad, goldstein, cameo* and *average* respectively. Note that we do not correct for multiple comparisons; such a correction would further reduce the significance of the results. We implement the comparison test for smoothed ROC curves detailed in Robin et al.[24].
  - 1 month forecasts: $Z = 0.64, 1.09, 0.42, 0.67$; $p = 0.26, 0.14, 0.34, 0.25$
  - 6 months forecasts: $Z = 0.41, 0.08, 0.70, 0.69$; $p = 0.34, 0.47, 0.24, 0.25$
- The 95% confidence intervals for the 1 month models are:
  - *escalation*: 0.66-0.95
  - *quad*: 0.63-0.95
  - *goldstein*: 0.62-0.93
  - *cameo*: 0.65-0.95
  - *average*: 0.65-0.95
- The 95% confidence intervals for the 6 month models are:
  - *escalation*: 0.64-0.93
  - *quad*: 0.60-0.90
  - *goldstein*: 0.68-0.93
  - *cameo*: 0.58-0.92
  - *average*: 0.60-0.92

While a small p-value is used to reject the null hypothesis (in this case — that the out-of-sample performance does not differ between the models being compared), a singular focus on a test for statistical significance at a pre-defined threshold can be harmful (see, for example Imbens[25]). Blair and Sambanis do report performance evaluations for a variety of different model specifications. However, the purpose of such

robustness checks is to determine whether model performance sensitive to the parameter choices; it is unclear whether it helps deal with issues arising from sampling variance. At any rate, Blair and Sambanis's results turn out to be highly sensitive to another modeling choice: the fact that they compute the AUC metric on the smoothed ROC curve instead of the empirical curve that their model produces. Smoothing refers to a transformation of the ROC curve to make the predicted probabilities for the war and peace instances normally distributed instead of using the empirical ROC curve (see Robin et al.[24]). This issue was pointed out by Beger, Morgan, and Ward[26] and completely changes their original results; Blair and Sambanis[27] discuss it in their rebuttal.

S2.7 Overview of papers in Table S6

Table S6 provides the list of 12 papers included in our review, showing information about whether they report confidence intervals, conduct tests of statistical significance when comparing classifier performance, which metrics they report, the number of rows and the number of positive instances (i.e. instances of war/conflict) in the test set, and whether their main claim relies on out-of-sample evaluation of classifier performance. We detail information about the numbers we report in Table S6 below.

- **Hegre et al.[28]**: We report the number of rows and number of positive instances of civil war incidence for the dates between 2001 and 2013 in the UCDP dataset, i.e. all years for which out-of-sample estimates are provided. We report the out-of-sample AUC performance difference for the Major conflict setting. Out-of-sample evaluation results are not included in the main text of the paper, hence we report that the paper's main claim does not rely on out-of-sample evaluations.

- **Muchlinski et al.[4]**: We report the number of rows and number of positive instances of civil war onset for the dates after 2000 in the out-of-sample dataset provided by Muchlinski et al. We report the out-of-sample AUC performance difference between the Random Forests and the best logistic regression setting. Out-of-sample evaluation results are used to justify the performance improvement of using Random Forests models, hence we report that the paper's main claim relies on out-of-sample evaluations.

- **Chiba and Gleditsch[29]**: We report the total number of instances and the number of positive instances of governmental onsets in the years 2013-14 (the test set dates). We report the difference between the territorial onset AUC's reported in the paper. Note that while Chiba and Gleditsch[29] do report small number of data points that are used in one of their settings, they do not address how to estimate variance or perform tests of statistical significance. Out-of-sample evaluation results are not used as the main evidence of better performance in the main text of the paper, hence we report that the paper's main claim does not rely on out-of-sample evaluations.

- **Colaresi and Mahmood[5]**: We report the number of rows and onsets of civil war after the year 1988 (the test set dates). We report the out-of-sample AUC difference between the two random forests models compared in the paper. Out-of-sample evaluation results are used to justify the performance improvement of using an iterative method for model improvement, hence we report that the paper's main claim relies on out-of-sample evaluations.

- **Hirose, Imai, and Lyall[30]**: We report the number of locations included in the out-of-sample results. Since the paper does not attempt binary classification, we do not report the number of positive instances in this case. We report the out-of-sample performance gain of adding relative ISAF support to the baseline model in the IED attack setting of the paper. Out-of-sample evaluation results are used as important evidence of better model performance in the main text of the paper, hence we report that the paper's main claim relies on out-of-sample evaluations.

- **Schutte[31]**: We report the number of rows in the entire dataset, since the paper uses k-fold cross validation and therefore all instances are used for testing. Since the paper does not attempt binary classification, we do not report the number of positive instances in this case. We report the out-of-sample normalized MAE difference between the population model and the best performing model compared in the paper. Out-of-sample evaluation results are used as important evidence of better

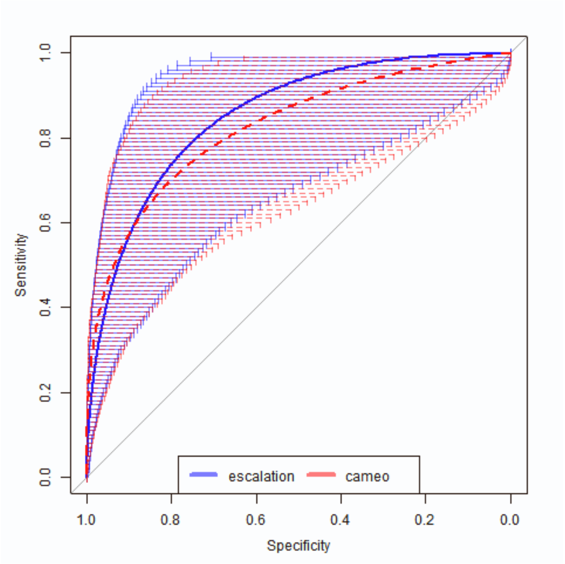

(a) Visualizing the 95% confidence intervals of the specificities for the 1 month forecast in the smoothed ROC curve reported in Blair and Sambanis[23].

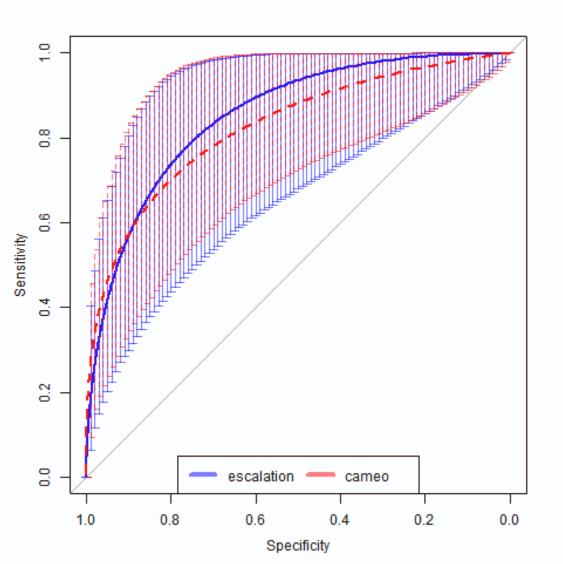

(b) Visualizing the 95% confidence intervals of the sensitivities for the 1 month forecast in the smoothed ROC curve reported in Blair and Sambanis[23].

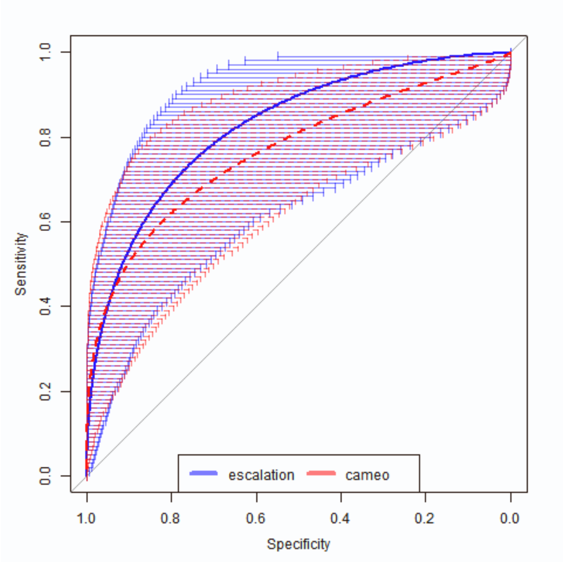

(c) Visualizing the 95% confidence intervals of the specificities for the 6 month forecast in the smoothed ROC curve reported in Blair and Sambanis[23].

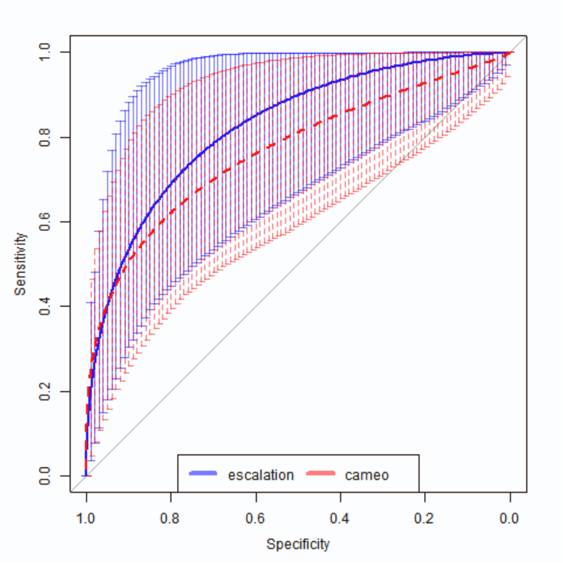

(d) Visualizing the 95% confidence intervals of the sensitivities for the 6 month forecast in the smoothed ROC curve reported in Blair and Sambanis[23].

Figure S3: The wide confidence intervals for sensitivities and specificities reported in Blair and Sambanis. Here, we visualize the *escalation* and *cameo* models for the 1 month and 6 month forecast in the base specification (reported in Figure 1 of their paper).

model performance in the main text of the paper, hence we report that the paper's main claim relies on out-of-sample evaluations.

- **Hegre, Hultman, and Nygård[32]**: We report the number of rows and number of positive instances of civil war incidence for the dates between 2001 and 2013 in the UCDP dataset, i.e. all years for which out-of-sample estimates are provided. We report the out-of-sample AUC performance difference for

the Major conflict setting. Out-of-sample evaluation results are not used as the primary evidence of better model performance in the main text of the paper, hence we report that the paper's main claim does not rely on out-of-sample evaluations.

- **Hegre et al.**[33]: We report the number instances with state based conflict in the ViEWS Monthly Outcomes at PRIO-Grid Level data between 2015 and 2017 — the years for which the out-of-sample results are reported in the paper. We report the out-of-sample AUC performance difference for the state-based conflict setting. Out-of-sample evaluation results are used as the primary evidence of better model performance in the main text of the paper, hence we report that the paper's main claim relies on out-of-sample evaluations.
- **Kaufman, Kraft, and Sen**[7]: We report the total number of rows and all instances of civil war incidence in the dataset used by Kaufman et al., since they use k-fold cross validation and therefore all instances are used for testing. We report the out-of-sample accuracy difference between the Adaboost and logistic regression settings. Out-of-sample evaluation results are used as the primary evidence of better model performance in the main text of the paper, hence we report that the paper's main claim relies on out-of-sample evaluations.
- **Wang**[6]: We report the total number of rows and onsets of civil war used in the dataset used by Wang since they use k-fold cross validation and therefore all instances are used for testing. We report the out-of-sample AUC performance difference between the Adaboost and logistic regression models. Out-of-sample evaluation results are used as the primary evidence of better model performance in the main text of the paper, hence we report that the paper's main claim relies on out-of-sample evaluations.
- **Blair and Sambanis**[23]: We report the number of rows and onsets of civil war after the year 2007 (the test set dates). We report the out-of-sample AUC performance difference between the escalation and cameo models for the one-month base setting. Out-of-sample evaluation results are used as the primary evidence of better model performance in the main text of the paper, hence we report that the paper's main claim relies on out-of-sample evaluations.
- **Hegre, Nygård, and Landsverk**[34]: We report the number of rows and number of positive instances for civil war onset the dates between 2001 and 2018, i.e. all years for which out-of-sample estimates are provided. We don't report the out-of-sample performance difference because the paper does not perform comparisons between models. Out-of-sample evaluation results are used as the primary evidence of model performance in the main text of the paper, hence we report that the paper's main claim relies on out-of-sample evaluations.

S3 Model info sheets can detect and prevent leakage in ML-based science

We include a template for model info sheets in the next section (Section S4). Here, we detail how model info sheets would address each type of leakage that we found in our survey, as well as the types of leakage we found in our case study of civil war prediction.

- **L1.1 No test set.** Model info sheets require an explanation of how the train and test set is split during all steps in the modeling process (Q9-17 of model info sheets).
- **L1.2 Pre-processing on training and test set.** Details of how the train and test set are separated during the preprocessing selection step need to be included in the model info sheet (Q12-13). In our civil war prediction case study, this would address leakage due to incorrect imputation[4–6].
- **L1.3 Feature selection on training and test set.** Details of how the train and test set are separated during the feature selection step need to be included in the model info sheet (Q14-15).
- **L1.4 Duplicates in datasets.** Model info sheets require details of whether there are duplicates in the dataset, and if so, how they are handled (Q10).

Paper	CI?	Stat. sig test?	Metric(s)	Num. rows in test set	Num. positive test set instances	Main Claim OOS?	OOS performance delta
Hegre et al.[28]	No	No	AUC, Brier score	2197	321	No	0.006
Muchlinski et al.[4]	No	No	AUC, F1 score	896	19	Yes	0.04
Chiba and Gleditsch[29]	No	No	AUC, Brier score	4176	15	No	0.03
Colaresi and Mahmood[5]	No	No	AUC, Precision, Recall	1778	29	Yes	0.02
Hirose, Imai, and Lyall[30]	No	*	MAE, RMSE	14,606	—	Yes	0.16
Schutte[31]	No	No	MAE	3744	—	Yes	0.09
Hegre, Hultman, and Nygård[32]	No	No	AUC	2197	321	No	0.02
Hegre et al.[33]	No	No	AUC, Brier score, AUPR, Accuracy, F1 score, cost-based threshold	384,372	1848	Yes	0.01
Kaufman, Kraft, and Sen[7]	No	No	Accuracy	6610	918	Yes	0.03
Wang[6]	Yes	No	AUC, Precision, Recall	6363	116	Yes	0.12
Blair and Sambanis[23]	No	No	AUC, Precision, Recall	15,744	11	Yes	0.03
Hegre, Nygård, and Landsverk[34]	Yes	No	AUC, AUPR, TPR/FPR	3042	79	Yes	—

Table S6: A list of papers for which code and dataset were available, showing information about whether they report confidence intervals, conduct tests of statistical significance when comparing classifier performance, which metrics they report, the number of rows and the number of positive instances (i.e. instances of war or conflict or onset thereof) in the test set, and whether their main claim relies on out-of-sample evaluation of classifier performance. AUC = Area Under ROC, MAE = Mean Absolute Error, RMSE = Root Mean Squared Error, AUPR = Area Under Precision-Recall Curve, TPR = True Positive Rate, FPR = False Positive Rate, OOS performance delta = the performance difference for the most salient performance comparison reported in the paper (details in Section S2.7). *Hirose et al. state that the out-of-sample performance is significantly better in the Supplement of their paper, but we could not find the figure they cite as evidence of this claim in their Supplement.

- **L2 Model uses features that are not legitimate.** For each feature used in the model, researchers need to argue why the feature is legitimate to be used for the modeling task at hand (Q21). This addresses the leakage due to the use of proxy variables in Kaufman, Kraft, and Sen[7].
- **L3.1 Temporal leakage.** In case the claim is about predicting future outcomes of interest based on ML methods, researchers need to provide an explanation for why the time windows used in the training and test set are separate, and why data in the test set is always a later timestamp compared to the data in the training set (Q20). This addresses the temporal leakage in Wang, Kaufman, Kraft, and Sen[6,7].
- **L3.2 Dependencies in training and test data.** Researchers need to reason about the dependencies that may exist in their dataset and outline how dependencies across training and test sets are addressed (Q11).
- **L3.3 Sampling bias in test distribution.** Researchers need to reason about the presence of selection bias in their dataset and outline how the rows included for data analysis were selected, and how the test set matches the distribution about which the scientific claims are made (Q18-19).

S4 Model Info Sheets Template

About model info sheets

Completing this model info sheet requires the researcher to provide precise arguments to justify that predictive models used for making scientific claims do not suffer from leakage. It is inspired by the model cards introduced by Mitchell et al.[1]

Model info sheets are intended to accompany the paper or report that introduces the model: for instance, as an appendix or supplemental material. For feedback or questions, contact: sayashk@princeton.edu

The model info sheet starts on the next page. After filling it out, save it starting from that page. To cite the paper that introduces the model info sheets, use the bibliography file available at reproducible.cs.princeton.edu/citation.bib

[1] Margaret Mitchell, Simone Wu, Andrew Zaldivar, Parker Barnes, Lucy Vasserman, Ben Hutchinson, Elena Spitzer, Inioluwa Deborah Raji, and Timnit Gebru. "Model cards for model reporting." In *Proceedings of the conference on fairness, accountability, and transparency*, 2019.

Model Info Sheet

Section 1: Information about paper or report

1) Author(s): Names of the authors of the paper or report

2) Title of the paper or report which introduces the model

3) DOI or permanent link to the paper or report (for example, link to arxiv.org webpage)

4) License: Under which license(s) are the data and/or model shared?

5) Email address of the corresponding author

Section 2: Scientific claim(s) of interest

6) Does your paper make a generalizable claim based on the ML model? If yes, what is the scientific claim? For example, "Our ML model can be used to diagnose Covid-19 using chest radiographs of adult patients".

If there are multiple claims, list each claim in a new line, along with a claim number.

7) Is the scientific claim made about a distribution or population from which you can sample? If yes: (a) what is the population or distribution about which the scientific claim is being made? (b) What is the sample used for the study? For example, "(a) Population: adult patients with symptoms of Covid-19. (b) Sample: We use a random sample of adult patients who present at a U.S. based hospital between April 2020 and June 2020".

If there are multiple scientific claims, list your answer for each claim in a new line, corresponding to their claim number in Q6.

Note: *A difference between the population and the set from which the sample is drawn could highlight potential generalizability failures, which are related to but distinct from leakage.*

8) Does the scientific claim only apply to certain subsets of the distribution mentioned in Q6? For example, "Our model works on chest radiographs of U.S.-based adult patients and might not generalize to radiographs taken in other places or using different machines."

If there are multiple claims, list your answer for each claim in a new line, corresponding to their claim number in Q6.

Section 3: Train-test split is maintained across all steps in creating the model

9) Train-test split type: How was the dataset split into train and test sets? (For example, cross-validation; separate train and test sets).

If your model does not have a separate test set, it could suffer from leakage due to overfitting

10) Are there duplicates in the dataset? If yes, explain how duplicates are handled to ensure the train-test split.

If duplicates from the training set are included in the test set, your model could suffer from leakage. The higher the percentage of duplicates in the test set, the more severe the leakage.

11) In case the dataset has dependencies (e.g., multiple rows of data from the same patient), describe how the dependencies were addressed (for example, using block-cross validation).

If dependencies across the train-test split are not addressed, your model could suffer from leakage. The higher the number of rows in the test set with dependencies, the more severe the leakage.

12) List all the pre-processing steps used in creating your model. For example, imputing missing data, normalizing feature values, selecting a subset of rows from the dataset for building the model.

13) How was the train-test split observed during each pre-processing step? If applicable, use a separate line for each step mentioned in Q12.

If the train-test split is not maintained during all pre-processing steps, your model could suffer from leakage.

14) List all the modeling steps used in creating your model. For example, feature selection, parameter tuning, model selection.

15) How was the train-test split observed during each modeling step? If applicable, use a separate line for each step mentioned in Q14.

If the train-test split is not maintained during all modeling steps, your model could suffer from leakage.

16) List all the evaluation steps used in evaluating model performance. For example, cross-validation, out-of-sample testing.

17) How was the train-test split observed during each evaluation step? If applicable, use a separate line for each step mentioned in Q16.

If the train-test split is not maintained during all evaluation steps, your model could suffer from leakage.

Section 4: Test set is drawn from the distribution of scientific interest.

18) Why is your test set representative of the population or distribution about which you are making your scientific claims?

If the test set distribution is different from the scientific claim of interest (listed in Q7), your model could suffer from leakage.

19) Explain the process for selecting the test set and why this does not introduce selection bias in the learning process.

Selection bias (for example, only choosing data from a given geographic location but expecting your model's performance to generalize to all locations) can lead to leakage.

20) In case your model is used to predict a future outcome of interest using past data, detail how data in the training set is always from a date earlier than the data in the test set.

In predictions about future outcomes of interest, using data from the future to predict in the training set the past in the test set is a form of leakage. Data in the training set should always have timestamps of an earlier time than those in the test set to avoid leakage.

Section 5: Each feature used in the model is legitimate for the task

21) List the features used in the model, alongside an argument for their legitimacy. A legitimate feature is one that would be available when the model is used in the real world and is not a proxy of the outcome being predicted. You can also include this list in an appendix and reference the relevant section of your Appendix here.

For example, “Patient age: We include this feature in our ML model for hypertension diagnosis since patient age is easily available in a clinical setting”.

An example of a feature that should not be included (for illustration only; you do not need to include these in your model info sheet): “Anti-hypertensive drugs: We do not include the use of anti-hypertensive drugs as a feature in our ML model for hypertension diagnosis since that information is only available after diagnosis and would not be available when a new patient presents with symptoms of hypertension.”

Note: *You do not need to list each feature used in your model here. However, you must provide an argument for the legitimacy of each feature included in your model to ensure that your model does not suffer from leakage due to illegitimate features. For example, “our model only uses data from the previous year as features. For instance, to predict civil war in 2017, we only use lagged features from the year 2016. Since these features are always available in advance of when we want to make predictions using our model, none of these features can lead to leakage.”*

References

[1] Engineering National Academies of Sciences. *Reproducibility and Replicability in Science*. en. May 2019. ISBN: 978-0-309-48616-3. DOI: 10.17226/25303. URL: https://www.nap.edu/catalog/25303/reproducibility-and-replicability-in-science (visited on 06/08/2021).

[2] David M. Liu and Matthew J. Salganik. "Successes and Struggles with Computational Reproducibility: Lessons from the Fragile Families Challenge". In: *Socius* 5 (2019), p. 2378023119849803. DOI: 10.1177/2378023119849803. eprint: https://doi.org/10.1177/2378023119849803. URL: https://doi.org/10.1177/2378023119849803.

[3] Max Kuhn and Kjell Johnson. *Applied Predictive Modeling*. en. New York: Springer-Verlag, 2013. ISBN: 978-1-4614-6848-6. DOI: 10.1007/978-1-4614-6849-3. URL: https://www.springer.com/gp/book/9781461468486 (visited on 05/17/2021).

[4] David Muchlinski et al. "Comparing Random Forest with Logistic Regression for Predicting Class-Imbalanced Civil War Onset Data". en. In: *Political Analysis* 24.1 (2016). Publisher: Cambridge University Press, pp. 87–103. ISSN: 1047-1987, 1476-4989. DOI: 10.1093/pan/mpv024. URL: https://www.cambridge.org/core/journals/political-analysis/article/abs/comparing-random-forest-with-logistic-regression-for-predicting-classimbalanced-civil-war-onset-data/109E1511378A38BB4B41F721E6017FB1 (visited on 05/16/2021).

[5] Michael Colaresi and Zuhaib Mahmood. "Do the robot: Lessons from machine learning to improve conflict forecasting". en. In: *Journal of Peace Research* 54.2 (Mar. 2017). Publisher: SAGE Publications Ltd, pp. 193–214. ISSN: 0022-3433. DOI: 10.1177/0022343316682065. URL: https://doi.org/10.1177/0022343316682065 (visited on 05/16/2021).

[6] Yu Wang. "Comparing Random Forest with Logistic Regression for Predicting Class-Imbalanced Civil War Onset Data: A Comment". en. In: *Political Analysis* 27.1 (Jan. 2019). Publisher: Cambridge University Press, pp. 107–110. ISSN: 1047-1987, 1476-4989. DOI: 10.1017/pan.2018.40. URL: https://www.cambridge.org/core/journals/political-analysis/article/comparing-random-forest-with-logistic-regression-for-predicting-classimbalanced-civil-war-onset-data-a-comment/B62CC1DA390C58435004D4C5D56DBF71 (visited on 05/16/2021).

[7] Aaron Russell Kaufman, Peter Kraft, and Maya Sen. "Improving Supreme Court Forecasting Using Boosted Decision Trees". en. In: *Political Analysis* 27.3 (July 2019). Publisher: Cambridge University Press, pp. 381–387. ISSN: 1047-1987, 1476-4989. DOI: 10.1017/pan.2018.59. URL: https://www.cambridge.org/core/journals/political-analysis/article/improving-supreme-court-forecasting-using-boosted-decision-trees/166AA006B8DA7C87F1B17291B0BB8B63 (visited on 05/16/2021).

[8] Daniel W. Hook, Simon J. Porter, and Christian Herzog. "Dimensions: Building Context for Search and Evaluation". English. In: *Frontiers in Research Metrics and Analytics* 3 (2018). Publisher: Frontiers. ISSN: 2504-0537. DOI: 10.3389/frma.2018.00023. URL: https://www.frontiersin.org/articles/10.3389/frma.2018.00023/full (visited on 05/13/2021).

[9] Corinne Bara. *Forecasting civil war and political violence*. en. Pages: 177-193 Publication Title: The Politics and Science of Prevision. Routledge, May 2020. ISBN: 978-1-00-302242-8. DOI: 10.4324/9781003022428-14. URL: https://www.taylorfrancis.com/https://www.taylorfrancis.com/chapters/oa-edit/10.4324/9781003022428-14/forecasting-civil-war-political-violence-corinne-bara (visited on 05/13/2021).

[10] A. Rogier T. Donders et al. "Review: A gentle introduction to imputation of missing values". en. In: *Journal of Clinical Epidemiology* 59.10 (Oct. 2006), pp. 1087–1091. ISSN: 0895-4356. DOI: 10.1016/j.jclinepi.2006.01.014. URL: https://www.sciencedirect.com/science/article/pii/S0895435606001971 (visited on 05/16/2021).

[11] Rayid Ghani, Joe Walsh, and Joan Wang. *Top 10 ways your Machine Learning models may have leakage (URL: http://www.rayidghani.com/2020/01/24/top-10-ways-your-machine-learning-models-may-have-leakage/)*. en-US. Jan. 2020. URL: http://www.rayidghani.com/2020/01/24/top-10-ways-your-machine-learning-models-may-have-leakage/ (visited on 05/16/2021).

[12] *Imputation before or after splitting into train and test?* URL: https://stats.stackexchange.com/questions/95083/imputation-before-or-after-splitting-into-train-and-test (visited on 05/16/2021).

[13] Tom Fawcett. "An introduction to ROC analysis". en. In: *Pattern Recognition Letters*. ROC Analysis in Pattern Recognition 27.8 (June 2006), pp. 861–874. ISSN: 0167-8655. DOI: 10.1016/j.patrec.2005.10.010. URL: https://www.sciencedirect.com/science/article/pii/S016786550500303X (visited on 05/16/2021).

[14] Marcel Neunhoeffer and Sebastian Sternberg. "How Cross-Validation Can Go Wrong and What to Do About It". en. In: *Political Analysis* 27.1 (Jan. 2019). Publisher: Cambridge University Press, pp. 101–106. ISSN: 1047-1987, 1476-4989. DOI: 10.1017/pan.2018.39. URL: https://www.cambridge.org/core/journals/political-analysis/article/how-crossvalidation-can-go-wrong-and-what-to-do-about-it/CA8C4B470E27C99892AB978CE0A3AE29 (visited on 05/31/2021).

[15] Jake M. Hofman et al. "Expanding the scope of reproducibility research through data analysis replications". en. In: *Organizational Behavior and Human Decision Processes* 164 (May 2021), pp. 192–202. ISSN: 0749-5978. DOI: 10.1016/j.obhdp.2020.11.003. URL: https://www.sciencedirect.com/science/article/pii/S0749597820304076 (visited on 07/13/2022).

[16] David Alan Muchlinski et al. "Seeing the Forest through the Trees". en. In: *Political Analysis* 27.1 (Jan. 2019). Publisher: Cambridge University Press, pp. 111–113. ISSN: 1047-1987, 1476-4989. DOI: 10.1017/pan.2018.45. URL: https://www.cambridge.org/core/journals/political-analysis/article/seeing-the-forest-through-the-trees/E717D15F10CC4F979EDC35C0CB9B55C1 (visited on 05/31/2021).

[17] Håvard Hegre and Nicholas Sambanis. "Sensitivity Analysis of Empirical Results on Civil War Onset:" en. In: *Journal of Conflict Resolution* (2006). Publisher: Sage PublicationsSage CA: Thousand Oaks, CA. DOI: 10.1177/0022002706289303. URL: https://journals.sagepub.com/doi/suppl/10.1177/0022002706289303 (visited on 06/02/2021).

[18] Andreas Beger. *@andybeega (Andreas Beger): This is great. One thing I'd add is that for the @DMuchlinski et al data...* http://archive.today/VV9nC. 2021. URL: http://archive.today/VV9nC (visited on 08/05/2021).

[19] Joseph L Schafer. "Multiple imputation: a primer". en. In: *Statistical Methods in Medical Research* 8.1 (Feb. 1999). Publisher: SAGE Publications Ltd STM, pp. 3–15. ISSN: 0962-2802. DOI: 10.1177/096228029900800102. URL: https://doi.org/10.1177/096228029900800102 (visited on 07/18/2021).

[20] James D. Fearon and David D. Laitin. "Ethnicity, Insurgency, and Civil War". In: *The American Political Science Review* 97.1 (2003). Publisher: [American Political Science Association, Cambridge University Press], pp. 75–90. ISSN: 0003-0554. URL: https://www.jstor.org/stable/3118222 (visited on 05/16/2021).

[21] Thomas G Dietterich. "Approximate statistical tests for comparing supervised classification learning algorithms". In: *Neural computation* 10.7 (1998), pp. 1895–1923.

[22] Paul Collier and Anke Hoeffler. "On the Incidence of Civil War in Africa". en. In: *Journal of Conflict Resolution* 46.1 (Feb. 2002). Publisher: SAGE Publications Inc, pp. 13–28. ISSN: 0022-0027. DOI: 10.1177/0022002702046001002. URL: https://doi.org/10.1177/0022002702046001002 (visited on 06/21/2021).

[23] Robert A. Blair and Nicholas Sambanis. "Forecasting Civil Wars: Theory and Structure in an Age of "Big Data" and Machine Learning". en. In: *Journal of Conflict Resolution* 64.10 (Nov. 2020). Publisher: SAGE Publications Inc, pp. 1885–1915. ISSN: 0022-0027. DOI: 10.1177/0022002720918923. URL: https://doi.org/10.1177/0022002720918923 (visited on 05/16/2021).

[24] Xavier Robin et al. "pROC: an open-source package for R and S+ to analyze and compare ROC curves". In: *BMC Bioinformatics* 12.1 (Mar. 2011), p. 77. ISSN: 1471-2105. DOI: 10.1186/1471-2105-12-77. URL: https://doi.org/10.1186/1471-2105-12-77 (visited on 07/24/2021).

[25] Guido W. Imbens. "Statistical Significance, p -Values, and the Reporting of Uncertainty". en. In: *Journal of Economic Perspectives* 35.3 (Aug. 2021), pp. 157–174. ISSN: 0895-3309. DOI: 10.1257/jep.35.3.157. URL: https://pubs.aeaweb.org/doi/10.1257/jep.35.3.157 (visited on 08/05/2021).

[26] Andreas Beger, Richard K. Morgan, and Michael D. Ward. "Reassessing the Role of Theory and Machine Learning in Forecasting Civil Conflict". en. In: *Journal of Conflict Resolution* (July 2021). Publisher: SAGE Publications Inc, p. 0022002720982358. ISSN: 0022-0027. DOI: 10.1177/0022002720982358. URL: https://doi.org/10.1177/0022002720982358 (visited on 07/26/2021).

[27] Robert A. Blair and Nicholas Sambanis. "Is Theory Useful for Conflict Prediction? A Response to Beger, Morgan, and Ward". en. In: *Journal of Conflict Resolution* (July 2021). Publisher: SAGE Publications Inc, p. 00220027211026748. ISSN: 0022-0027. DOI: 10.1177/00220027211026748. URL: https://doi.org/10.1177/00220027211026748 (visited on 07/26/2021).

[28] H\aavard Hegre et al. "Forecasting civil conflict along the shared socioeconomic pathways". en. In: *Environmental Research Letters* 11.5 (Apr. 2016). Publisher: IOP Publishing, p. 054002. ISSN: 1748-9326. DOI: 10.1088/1748-9326/11/5/054002. URL: https://doi.org/10.1088/1748-9326/11/5/054002 (visited on 06/26/2021).

[29] Daina Chiba and Kristian Skrede Gleditsch. "The shape of things to come? Expanding the inequality and grievance model for civil war forecasts with event data". en. In: *Journal of Peace Research* 54.2 (Mar. 2017). Publisher: SAGE Publications Ltd, pp. 275–297. ISSN: 0022-3433. DOI: 10.1177/0022343316684192. URL: https://doi.org/10.1177/0022343316684192 (visited on 06/26/2021).

[30] Kentaro Hirose, Kosuke Imai, and Jason Lyall. "Can civilian attitudes predict insurgent violence? Ideology and insurgent tactical choice in civil war". en. In: *Journal of Peace Research* 54.1 (Jan. 2017). Publisher: SAGE Publications Ltd, pp. 47–63. ISSN: 0022-3433. DOI: 10.1177/0022343316675909. URL: https://doi.org/10.1177/0022343316675909 (visited on 06/26/2021).

[31] Sebastian Schutte. "Regions at Risk: Predicting Conflict Zones in African Insurgencies*". en. In: *Political Science Research and Methods* 5.3 (July 2017). Publisher: Cambridge University Press, pp. 447–465. ISSN: 2049-8470, 2049-8489. DOI: 10.1017/psrm.2015.84. URL: https://www.cambridge.org/core/journals/political-science-research-and-methods/article/abs/regions-at-risk-predicting-conflict-zones-in-african-insurgencies/4DCDBA2BCC8B4E3D5057A2C37DDB2BD6 (visited on 06/26/2021).

[32] Håvard Hegre, Lisa Hultman, and Håvard Mokleiv Nygård. "Evaluating the Conflict-Reducing Effect of UN Peacekeeping Operations". In: *The Journal of Politics* 81.1 (Jan. 2019). Publisher: The University of Chicago Press, pp. 215–232. ISSN: 0022-3816. DOI: 10.1086/700203. URL: https://www.journals.uchicago.edu/doi/10.1086/700203 (visited on 06/26/2021).

[33] Håvard Hegre et al. "ViEWS: A political violence early-warning system:" en. In: *Journal of Peace Research* (Feb. 2019). Publisher: SAGE PublicationsSage UK: London, England. DOI: 10.1177/0022343319823860. URL: https://journals.sagepub.com/doi/suppl/10.1177/0022343319823860 (visited on 01/08/2021).

[34] Håvard Hegre, Håvard Mokleiv Nygård, and Peder Landsverk. "Can We Predict Armed Conflict? How the First 9 Years of Published Forecasts Stand Up to Reality". In: *International Studies Quarterly* sqaa094 (Jan. 2021). ISSN: 0020-8833. DOI: 10.1093/isq/sqaa094. URL: https://doi.org/10.1093/isq/sqaa094 (visited on 06/26/2021).
